# M_6_L_12_ Nanospheres with Multiple C_70_ Binding Sites for ^1^O_2_ Formation in Organic and Aqueous Media

**DOI:** 10.1021/jacs.2c05507

**Published:** 2022-08-17

**Authors:** Eduard O. Bobylev, David A. Poole, Bas de Bruin, Joost N.H. Reek

**Affiliations:** † van ’t Hoff Institute for Molecular Sciences, University of Amsterdam, Science Park 904, 1098 XH Amsterdam The Netherlands

## Abstract

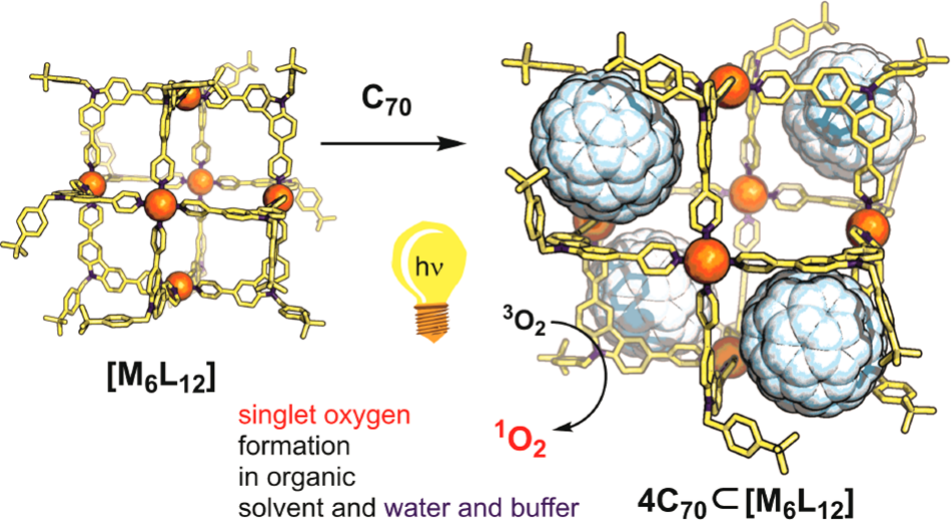

Singlet oxygen is a potent oxidant with major applications in organic synthesis and medicinal treatment. An efficient way to produce singlet oxygen is the photochemical generation by fullerenes which exhibit ideal thermal and photochemical stability. In this contribution we describe readily accessible M_6_L_12_ nanospheres with unique binding sites for fullerenes located at the windows of the nanospheres. Up to four C_70_ can be associated with a single nanosphere, presenting an efficient method for fullerene extraction and application. Depending on the functionality located on the outside of the sphere, they act as vehicles for ^1^O_2_ generation in organic or in aqueous media using white LED light. Excellent productivity in ^1^O_2_ generation and consecutive oxidation of ^1^O_2_ acceptors using C_70_⊂[Pd_6_L_12_], C_60_⊂[Pd_6_L_12_] or fullerene soot extract was observed. The methodological design principles allow preparation and application of highly effective multifullerene binding spheres.

## Introduction

**Figure 1:**
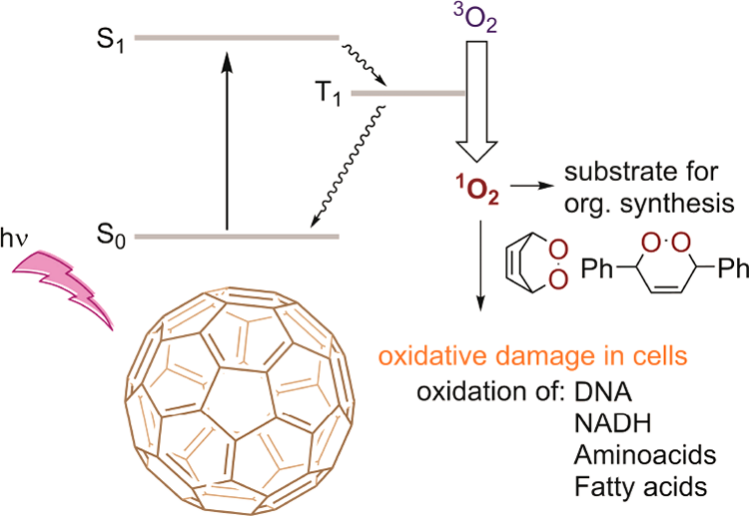
Schematic picture of the mechanism of photochemical generation of singlet oxygen by fullerene.

**Figure 2:**
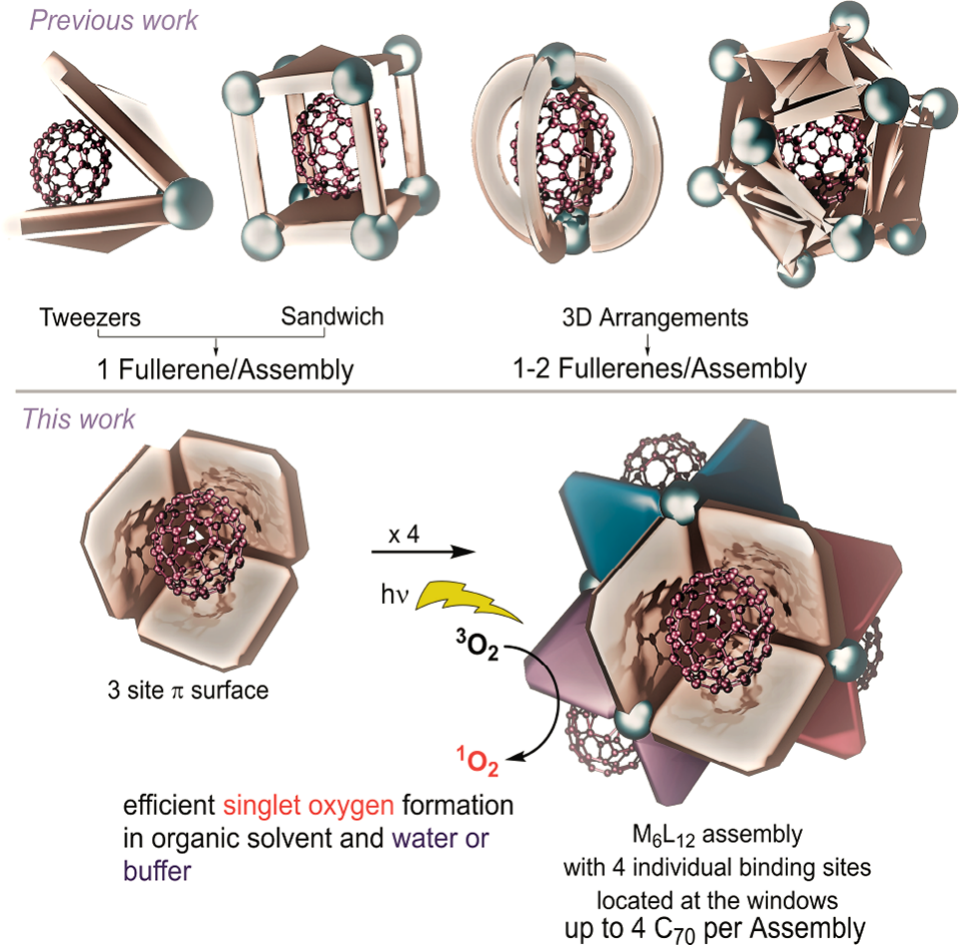
Illustration of fullerene binding hosts based on coordination driven self-assembly (top). Design strategy for a multiple-fullerene binding assembly (bottom).

Singlet oxygen, an electronically excited form of oxygen, has numerous applications in synthetic chemistry,^1,2^ purification,^3^ and pharmacology^4−6^ due to its strong oxidizing properties.^7^ During the last decades, many synthetic protocols were developed based on the reactivity of ^1^O_2_ with C–H bonds, C=C double bonds, aromatic systems, and heteroatoms (Figure 1).^1^ Singlet oxygen finds major application in the clinical photodynamic therapy treatment (PDT) of tumors in which oxidative stress caused by ^1^O_2_ leads to cell damage or cell death (Figure 1).^6^ Generation of singlet oxygen can be achieved via different methods. Apart from stoichiometric chemical reactions, photochemical excitation of an endogenous photosensitizer and transfer of its excitation energy describe one of the most common methods of ^1^O_2_ generation (Figure 1). Classically, organic dyes, such as rose bengal or methylene blue, are applied as a photosensitizer.^8,9^ Clinical trials using these photosensitizers in PDT are currently pursued;^10^ however, these conventional dyes are prone to chemical, photoinduced or enzymatic degradation, limiting their application *in vivo* and lowering their overall efficiency in synthetic chemistry.^11^ These major challenges related to PDT can be circumvented using fullerenes, which exhibit ideal stability and good absorbance in the visible light, for photochemical generation of ^1^O_2_ (Figure 1).^12,13^ However, application of fullerenes for *in vivo*^1^O_2_ generation or for oxidation reactions for synthetic purposes is often hampered by their poor solubility in most solvents, including water. Therefore, there is an interest in structures that bind fullerene to allow fullerene application in a wide variety of media. Common design features of supramolecular structures that bind fullerene include the use of π surfaces that allow good interaction with the aromatic surface of the fullerene. With that in mind, coordination-based self-assemblies with fullerene binding capability can be separated into three different design types (Figure 2). Tweezers are a relatively simple, yet effective structure for fullerene binding.^14−17^ These tweezers typically consist of two aromatic surfaces which are connected by either coordination chemistry or by covalent bonds.

A second type are those with a sandwich type arrangement. Such a structure has two π surfaces, on top and at the bottom, that are connected to one another through different types of linkers.^18−20^ The third type consists of three-dimensional cages, capsules, or barrels that surround the fullerene on all sites allowing multiple π-interactions to facilitate binding.^21−29^ Most reported spheres bind only 1 fullerene, and only a few examples have been reported in which the host binds multiple fullerene guests.^30−34^ Binding multiple fullerenes to a single sphere can not only enhance the fullerene extraction efficiency of the spheres, but can also lead to useful electronic and spectroscopic properties for catalytic applications or preparation of functional materials (such as electron storage devices).^33,35^

To further boost the widespread application of fullerenes, easily accessible and robust structures that effectively bind fullerenes are highly desirable. Here, we present a straightforward strategy to prepare cubic M_6_L_12_ nanospheres that have four independent binding sites for fullerene, which can be readily prepared from commercial materials (Figure 2). We introduce a new design in which fullerene binding occurs at the windows of the self-assembled structure, leading to efficient binding under various conditions. Depending on the structure of the applied building blocks, high binding affinities for fullerenes are realized, leading to novel materials which bear up to four C_70_ bound to a single nanosphere. The application of functionalized building blocks used for the self-assembly result in nanospheres with various *exo*-functionalization, enabling the binding of fullerene in various organic solvents and even in water. An exploration of their ability to produce ^1^O_2_ in a variety of media and subsequent oxidation of ^1^O_2_ acceptors revealed high productivity using C_70_⊂[M_6_L_12_] or materials in which fullerenes were directly extracted from fullerene soot using [M_6_L_12_]. The availability of the herein reported nanospheres together with their ability in ^1^O_2_ generation in various media allow for a more efficient, sustainable application in organic synthesis. The general design principles provide a useful strategy for the construction of novel water-soluble fullerene-binding cages, which are potentially suitable for PDT. With the general simple design principles, we hope to inspire further development of multiple-fullerene binding structures and their widespread applications.

## Results and Discussion

**Figure 3:**
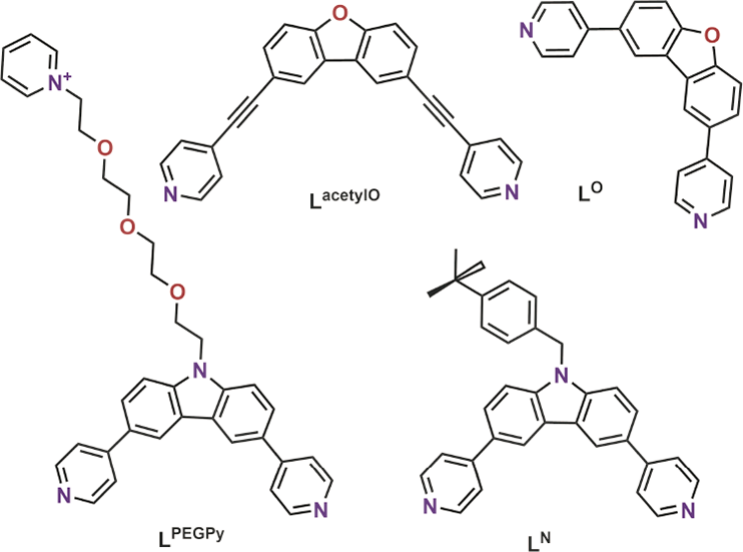
Structure of the herein investigated ditopic ligand building blocks used for the preparation of Pd_6_L_12_ nanospheres.

Inspired by a fullerene binding system developed by Mukherjee and Stang^36^ and by square shaped Pd_6_L_12_ nanospheres developed by Fujita,^37^ we designed four different building blocks with similar dibenzofuran/carbazole cores. Two of the building blocks **L**^**acetylO**^ and **L**^**O**^ were chosen in order to study the influence of the rigidity and sphere size on fullerene binding properties (Figure 3). Both are easily obtained in a one-step procedure via Sonogashira or Suzuki cross-coupling from 2,6-dibromo-dibenzofurane in excellent yields (section S1). Two other types of building blocks were derived from carbazole **L**^**N**^ and **L**^**PEGPy**^ (Figure 3). Both building blocks (**L**^**N**^ and **L**^**PEGPy**^) are more electron rich, allowing stronger interactions with fullerene.^38−40^**L**^**N**^ has an extra benzene moiety to potentially increase the π–π interactions between the host and the guest and to provide better solubility in organic solvents. **L**^**PEGPy**^ has a hydrophilic group attached to the ligand, making it suitable for the preparation of water-soluble nanospheres. All herein presented ligands have a dihedral angle of ∼90° between the pyridine donors and should therefore form Pd_6_L_12_ spheres upon coordination with palladium, as has been shown before for **L**^**acetylO**^ and **L**^**O**^.^38,42^

**Figure 4:**
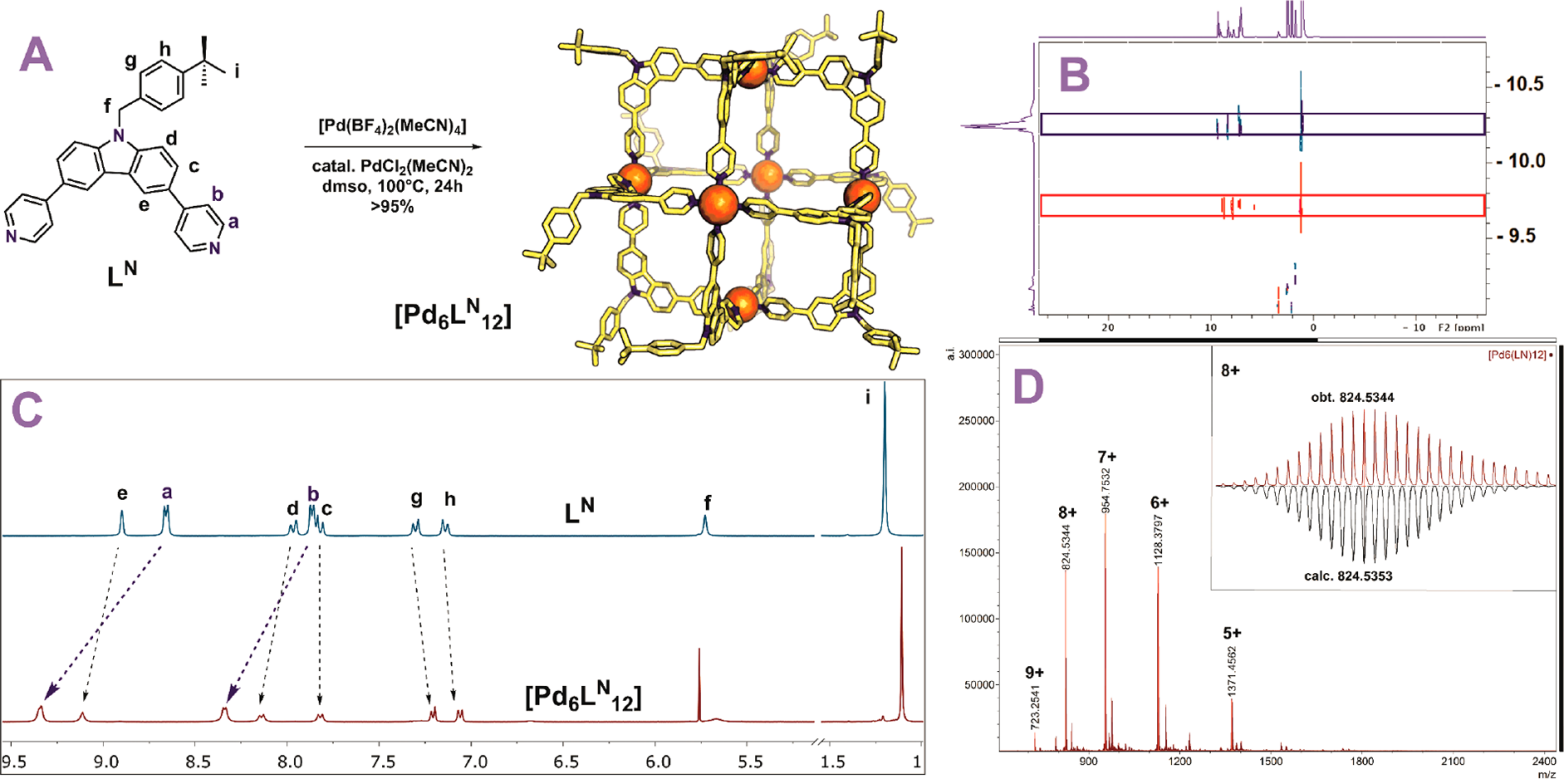
Characterization of Pd_6_**L**^**N**^_12_. (A) Reaction conditions for formation of nanospheres. Molecular structure of the displayed sphere was minimized at the PM3 level. Carbon is displayed in yellow, nitrogen in blue, palladium as orange spheres. (B) Overlayed DOSY NMR of the [Pd_6_**L**^**N**^_12_] sphere (blue) and the building block (red). (C) ^1^H NMR spectra of [Pd_6_**L**^**N**^_12_] sphere and the corresponding building block. (D) ESI-MS spectrum of [Pd_6_**L**^**N**^_12_].

Sphere formation was performed by mixing 1 equiv of **L**^**N**^ with 0.6 equiv [Pd(BF_4_)_2_(MeCN)_4_] and 5 mol % PdCl_2_(MeCN)_2_ as catalyst in dimethyl sulfoxide (DMSO) at 100 °C for 24 h according to a previously reported procedure^41^ (Figure 4A). After this period, one clear set of protons was observed in the ^1^H NMR spectrum of this solution, implying the formation of a highly symmetrical structure (Figure 4C). A downfield shift of the pyridyl protons was observed in accordance to coordination to palladium (signal a and b, Figure 4C). Diffusion ordered NMR (DOSY) displayed one signal corresponding to a hydrodynamic radius of 2 nm in line with the formation of [Pd_6_**L**^**N**^_12_] nanosphere (Figure 4B). ESI-MS analysis supported the formation of the desired [Pd_6_**L**^**N**^_12_] sphere, as it displayed only signals corresponding to different charged states of [Pd_6_**L**^**N**^_12_]^*x*+^ for *x* = 5–9 (Figure 4D).

All other spheres [Pd_6_**L**^**acetylO**^_12_], [Pd_6_**L**^**O**^_12_], and [Pd_6_**L**^**PEGPy**^_12_] were obtained by identical experimental procedures to [Pd_6_**L**^**N**^_12_], featuring all characteristic spectroscopic features similar to [Pd_6_**L**^**N**^_12_] (section S2). All spheres were obtained in excellent yields (>95%, based on ^1^H NMR and MS analysis, section S2) and used as such for subsequent investigations.

### Fullerene Binding Studies

**Figure 5:**
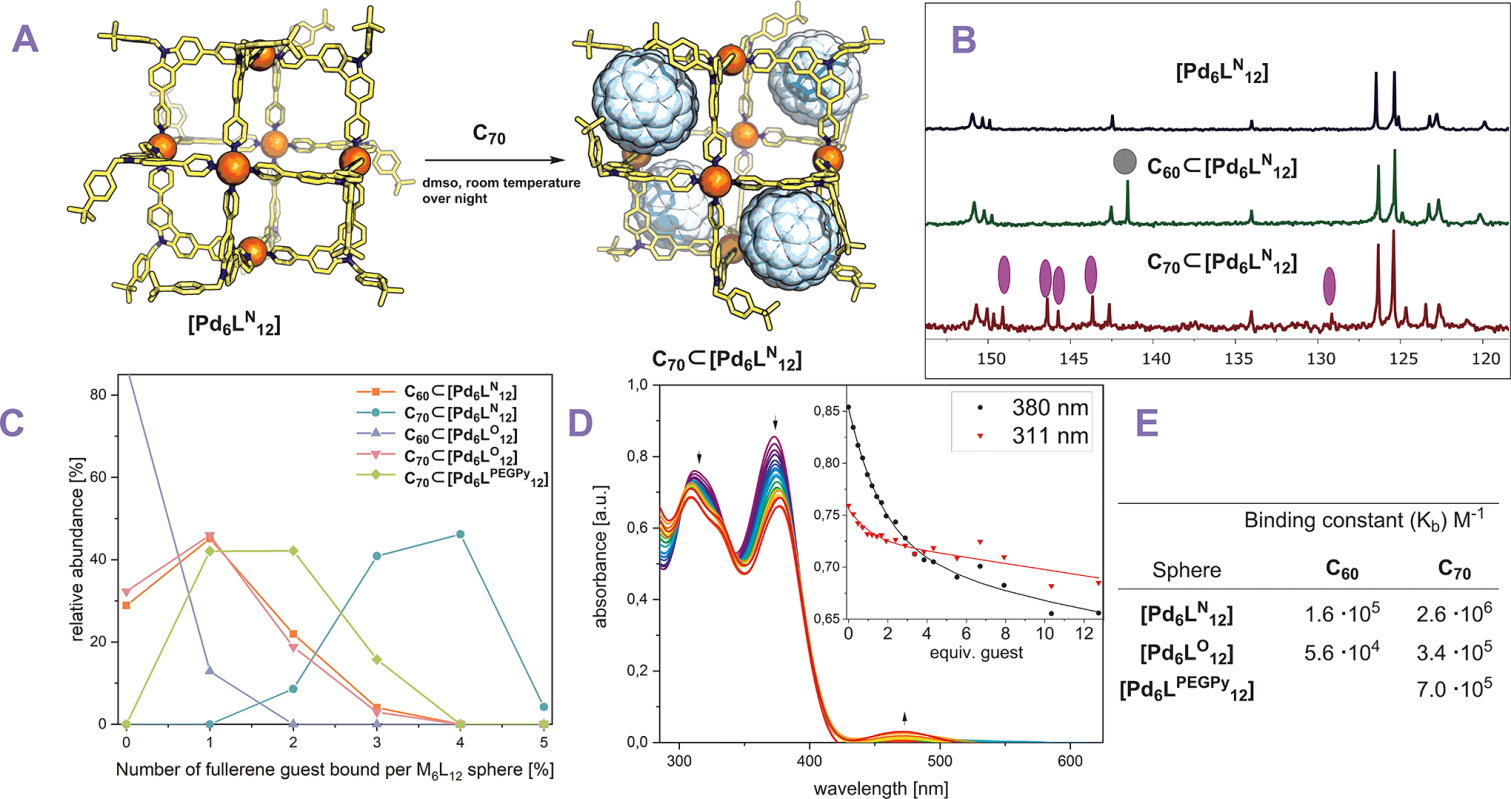
Fullerene binding assay of Pd_6_L_12_ nanospheres. (A) Reaction conditions for formation of host–guest complexes. Molecular structure of the displayed assembly was minimized at the PM3 level. Carbon is displayed in yellow, nitrogen in blue, palladium as orange spheres, and fullerene C_70_ as white spheres. (B) ^13^C NMR spectra of [Pd_6_**L**^**N**^_12_] nanosphere and the corresponding fullerene adducts. (C) Distribution of fullerenes bound to different types of nanospheres based on ESI-MS analysis. (D) Example of an UV–vis titration of C_70_ to a solution of [Pd_6_**L**^**N**^_12_]. Inset: 1:2, H/G binding fit on changes of two different wavelengths. (E) Binding constant of fullerene to different types of spheres obtained by UV–vis titrations.

Fullerene binding experiments were performed by the addition of solid fullerene to DMSO solutions containing the sphere (Figure 5A). The resulting suspensions were stirred at room temperature overnight, filtered, and analyzed by different analytical techniques. Because fullerenes have negligible solubility in DMSO, the presence of characteristic spectroscopic features related to fullerenes can be attributed to binding.

After a mixture of solid C_60_ and a solution of [Pd_6_**L**^**acetylO**^_12_] nanospheres was stirred, no color change of the solution was observed. ^1^H- and ^13^C NMR did not display any difference in the spectra and MS analysis of the solution displayed only signals corresponding to the free [Pd_6_**L**^**acetylO**^_12_] nanosphere. Apparently, there is no strong interaction between [Pd_6_**L**^**acetylO**^_12_] and fullerene C_60_. Mixing C_70_ and [Pd_6_**L**^**acetylO**^_12_] also did not change the spectroscopic features, indicating no binding of C_70_ either.

Interestingly, mixing solid C_70_ with a solution of [Pd_6_**L**^**O**^_12_], which is the nanosphere based on a ditopic ligand with only aromatic rings, leads to a color change of the solution from colorless to red-brown. In line with this, an additional absorption was observed in the UV–vis spectrum between 400 and 500 nm, which is characteristic for C_70_ (Figure S33). ^13^C NMR displayed one new set of signals which can be attributed to C_70_, indicating the presence of C_70_ in solution, as a result of binding to [Pd_6_**L**^**O**^_12_] (Figure S26). ESI-MS analysis of solutions containing [Pd_6_**L**^**O**^_12_] and C_70_ displayed a range of signals corresponding to host–guest complexes (Figure S31). The most dominant species was attributed to (C_70_)_1_⊂[Pd_6_**L**^**O**^_12_] with a distribution around this main species (Figure 5C). For C_60_ and [Pd_6_**L**^**O**^_12_], a slight color change was observed (with a weak absorption above 350 nm). ^13^C NMR showed the presence of C_60_ in solution (Figure S26). Furthermore, ESI-MS analysis displayed a range of signals corresponding to C_60_⊂[Pd_6_**L**^**O**^_12_] (Figure S28). Compared to a reaction mixture with C_70_ and [Pd_6_**L**^**O**^_12_], the spectroscopic features and the peaks in the MS spectra attributed to C_60_ bound to [Pd_6_**L**^**O**^_12_] were less intense, indicating a weaker affinity of the sphere for fullerene C_60_ than for C_70_. On the basis of these initial results that suggest stronger binding of fullerenes to nanospheres based on ligand building blocks containing aromatic rings only, that is, the absence of the acetylene bridge between the aromatic units in the building block (**L**^**acetylO**^), we next investigated the binding to the nanosphere based on the carbazole building block without any acetylene linkers.

Stirring a mixture of solid C_70_ and a DMSO solution of [Pd_6_**L**^**N**^_12_] resulted in a color change from light yellow to dark brown/red. An additional absorption between 400 and 500 nm appeared in the UV–vis spectrum indicative of C_70_ binding (Figure S50). ^13^C NMR displayed all signals corresponding to the [Pd_6_**L**^**N**^_12_] nanosphere and signals which can be attributed to C_70_ (Figure 5B). ESI-MS analysis of the solution displayed multiple species with (C_70_)_4_⊂[Pd_6_**L**^**N**^_12_] giving the most pronounced signal with a distribution around this stoichiometry (Figure S46 and Figure 5C). Interestingly, the highest peaks in the MS spectra are those of the host–guest complex with a stoichiometry of 1:4, with only small peaks corresponding to (C_70_)_5_⊂[Pd_6_**L**^**N**^_12_]. The nanosphere has in total eight pockets which are available for fullerene binding (Figure 4A, discussion on MS distribution can be found in the Supporting Information, section S8). However, fullerene binding to a pocket withdraws electron density from the adjacent aromatic linkers of the nanosphere and possibly bends the linker framework toward the bound fullerene. As a result, the empty pockets adjacent to those that bind a fullerene may therefore bind with lower affinity. Therefore, while the sphere consists of eight binding pockets, it contains only four independent binding pockets (Figure 5A). Our MS experiments show that four binding pockets are occupied by C_70_ in the [Pd_6_**L**^**N**^_12_] nanosphere (as displayed in Figure 5A, the found 1:5 will be discussed later). Interestingly, also mixtures of C_60_ and [Pd_6_**L**^**N**^_12_] displayed a color change to brown. ^13^C NMR displayed a signal which can be attributed to C_60_ (Figure 5B). Also, ESI-MS analysis of the solution displayed multiple species with (C_60_)_1_⊂[Pd_6_**L**^**N**^_12_] being the most present species (Figure S43 and Figure 5C). The lower amount of C_60_ associated with [Pd_6_**L**^**N**^_12_] (according to ESI-MS analysis, Figure 5C) than C_70_ indicates a stronger binding for C_70_ over C_60_.

Similar studies using the [Pd_6_**L**^**PEGPy**^_12_] nanosphere showed that a mixture of host–guest complexes formed, with a different number of C_70_ bound to the sphere, as judged by the MS data (Figure S56). The species with 1 or 2 C_70_ per nanosphere were dominant as indicated by ESI-MS distribution analysis (Figure 5C). The average number of fullerenes C_70_ bound to a single [Pd_6_**L**^**PEGPy**^_12_] sphere is 1.5 C_70_ and is in between the average number of C_70_ bound to [Pd_6_**L**^**N**^_12_] (3.5 C_70_) and to [Pd_6_**L**^**O**^_12_] (1 C_70_) (Figure 5C). These experiments suggest that both the higher electron density and extra aromatic rings on the ditopic ligand building block (**L**^**N**^) located at the building block contribute significantly to better binding of the fullerene guest.

Next to qualitative analysis of the fullerene-sphere host guest complexes using MS analysis, their binding constants were determined by UV–vis titrations (Figure 5E; for details and elaborate discussion see section S3). Due to the solubility limitation of fullerenes in DMSO, stock solutions of fullerene in toluene were used for these titrations. While the binding may be affected by the presence of toluene, the binding constants obtained provide a relative binding affinity and a lower limit of the binding constant. Upon addition of the C_70_ (or C_60_) fullerene (in toluene) to a solution of the sphere (in DMSO), changes in the UV–vis spectra are observed. The main absorption corresponding to the spheres (374 nm for [Pd_6_**L**^**N**^_12_]/[Pd_6_**L**^**PEGPy**^_12_] and 320 nm for [Pd_6_**L**^**O**^_12_]) decreased, whereas signals associated with the fullerene increased (Figure 5D). As discussed previously, Pd_6_L_12_ nanospheres are multivalent receptors for fullerenes with four independent binding pockets (Figure 5A). As a starting point, we fitted the obtained binding curves of the titration of C_70_ to [Pd_6_**L**^**N**^_12_] using a noncooperative 1:4 or 1:3 model. This gave a binding curve with a large error (20%), a sigmoidal shaped curve and large covariances (Figures S19–S21), implying that a noncooperative 1:4 model is not a good description of the system under diluted UV–vis conditions.^42^ As the binding in the presence of toluene as cosolvent may be weaker, we anticipated low contributions of the third and fourth binding at the low concentrations typically used for UV–vis. When we fitted the binding curve in a noncooperative 1:2 model in order to determine the binding strength between C_70_ and [Pd_6_**L**^**N**^_12_], a better fit was obtained with a lower error (6%) and lower covariances (Figure S23, for elaborate discussion see section S3). Therefore, we employed a 1:2 binding model instead of the 1:4 model for a rough estimation of all binding constants. All binding constants were obtained in good accuracy (error <10%). In agreement with our MS distribution analysis, [Pd_6_**L**^**N**^_12_] showed the highest binding constant for C_70_ (2.6 ± 0.16 × 10^6^ M^–1^) and [Pd_6_**L**^**O**^_12_] binds C_70_ the weakest (3.4 ± 0.19 × 10^5^ M^–1^) (Figure 5E). In line with our MS data, [Pd_6_**L**^**PEGPy**^_12_] displayed a binding constant for C_70_ in between that found for [Pd_6_**L**^**O**^_12_] and [Pd_6_**L**^**N**^_12_] (7.0 ± 0.32 × 10^5^ M^–1^). The same trend was found for the binding C_60_ 1.6 ± 0.05 × 10^5^ M^–1^ for [Pd_6_**L**^**N**^_12_] and 5.6 ± 0.31 × 10^4^ M^–1^ found for [Pd_6_L^O^_12_]. In summary, dibenzofurane and carbazole moieties as part of sphere forming building blocks generate nanospheres that allow fullerene binding. Fully aromatic building blocks show better binding than elongated (acetylene linked) ones. Their binding ability can easily be improved by increasing the electron density of the aromatic group at the building block (carbazole > dibenzofurane). The binding can be further increased by the introduction of extra aromatic moieties on the carbazole nitrogen (**L**^**N**^ > **L**^**PEGPy**^).

### Computational Investigation of Binding

**Figure 6:**
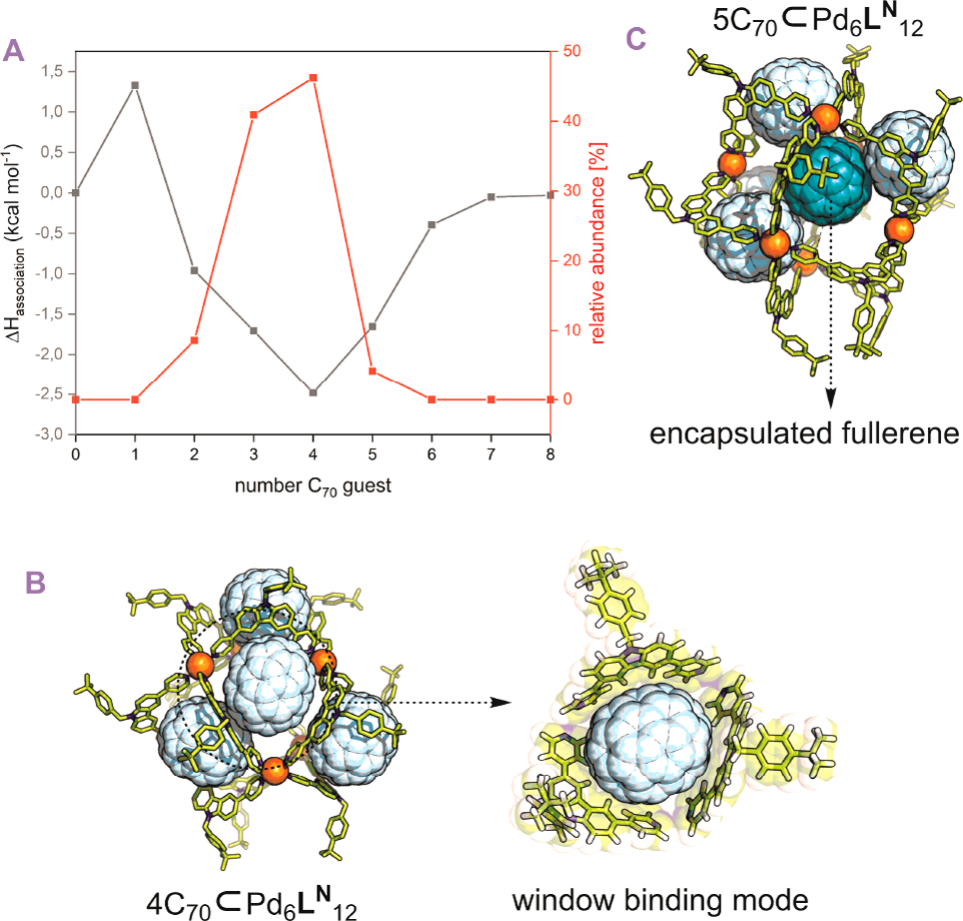
Computational investigation on C_70_ binding of [Pd_6_**L**^**N**^_12_] using molecular dynamics (MD). (A) Display of averaged total association enthalpies for different amount of C_70_ bound to a single sphere and the obtained distribution of C_70_ associated with [Pd_6_**L**^**N**^_12_] using the MS analysis. (B) Optimized structure of four C_70_ associated with a single sphere, displaying the window binding motif. (C) Optimized structure of 5 C_70_ associated with a single sphere, displaying the creation of a hydrophobic interior binding site for the fifth C_70_.

To get further structural insights into the binding stoichiometry of C_70_ to [Pd_6_**L**^**N**^_12_], we studied the complex *in silico* using molecular dynamics (MD). Our MD models were parametrized following our previously developed protocols.^43^ Model environments were constructed to feature Pd_6_**L**^**N**^_12_ and 0–8 C_70_ positioned randomly within the cage using ProFit.^44^ These structures were annealed in explicitly solvated MD simulations (2000 molecules DMSO, 12 molecules BF_4_^–^) for 50 ns at 300 K. Annealed structures were then optimized, and association enthalpies (Δ*H*) were estimated by a MMGBSA approach (a technique for estimating the energy of association from energy differences due to host/guest interaction) (Figure 6A, black trace).^45^ These simulations showed that C_70_ bound preferentially in the windows of [Pd_6_**L**^**N**^_12_] (Figure 6B) due to the fitting size. While the first C_70_ binding is enthalpically unfavorable (Δ*H*^1^ = 1.30 kcal·mol^–1^), associations of up 2–6 C_70_ guests is enthalpically favored with an optimum of four guest molecules per cage (Δ*H*_4_ = −2.48 kcal·mol^–1^) in line with our HRMS results (Figure 6A, red trace). This preference for multiple guest binding (2–6 C_70_) arises from favorable guest–guest interactions (π–π stacking) within the capsule. When 3–4 fullerenes are associated with the windows of a sphere, a π-rich binding site is created on the interior space of the sphere, facilitating the further association of a fifth C_70_ (Figure 6C, Figure S45). We anticipate this π-rich environment may facilitate the encapsulation of guest substrate molecules as a biomimetic active site, benefiting photocatalytic applications (see discussion S10). These calculations provide a good explanation why we observe mostly a 4:1 complex by ESI-MS from samples in which the fullerene was extracted using nanosphere solutions in DMSO. As the binding constants were obtained from titration experiments carried out in toluene–DMSO mixtures, quantitative comparison of these data is difficult.

### Photocatalytic Formation of ^1^O_2_

Although fullerenes have ideal photostability and efficiency in ^1^O_2_ generation, their broad applicability in singlet oxygen generation is limited due to their limited solubility (Table 1, right). Typically, only rather apolar solvents such as benzene and chloroform allow for sufficient concentrations of fullerene. Therefore, substrates which do not dissolve in these rather apolar solvents cannot be efficiently oxidized using fullerene-mediated photogenerated ^1^O_2_. To extend the application of fullerenes to water and polar solvents, which are generally suitable for many organic compounds and materials, fullerene-binding spheres can act as vehicles which allow solubility in these solvents. Given the strong binding between the fully aromatic spheres [Pd_6_**L**^**N**^_12_], [Pd_6_**L**^**O**^_12_], and [Pd_6_**L**^**PEGPy**^_12_] with fullerenes, their application in singlet oxygen generation in different solvents and consecutive oxidation of model substrates was studied. First, the conversion of anthracene (which is a well-known aromatic singlet oxygen acceptor) was studied (Table 1, entry 1–5). In the absence of any photosensitizer, irradiation of a solution containing anthracene with white LED light showed no conversion, showing that there is no background reaction. We started the photocatalytic ^1^O_2_ based reactions using fullerene C_70_ (as it has a higher visible light absorbance than C_60_) bound to various cages in different solvents. The solvent compatibility using C_70_⊂[Pd_6_**L**^**N**^_12_] was explored and compared to experiments in which the free C_70_ was used (Table 1). As expected, in benzene and chloroform both free C_70_ and using C_70_⊂[Pd_6_**L**^**N**^_12_] acted as good photocatalyst, and there was hardly any difference in conversion (entry 1 and 2). In contrast, the C_70_⊂[Pd_6_**L**^**N**^_12_] system showed to be an excellent candidate for ^1^O_2_ generation and consecutive oxidation of anthracene in more polar organic solvents, including acetone, acetonitrile, and dimethylformamide, and in these solvents the free C_70_ resulted typically in low yields. Because free C_70_ displayed good activity in apolar solvents, the enhanced activity of the sphere-fullerene complex in comparison to free C_70_ in polar solvents can mainly be attributed to the enhanced solubility (discussion on other effects can be found in section S10). Reactions in polar solvents using C_70_⊂[Pd_6_**L**^**N**^_12_] system resulted in high yields and turn over number (TON > 2000, Table 1, entries 3–5).

**Table 1:**
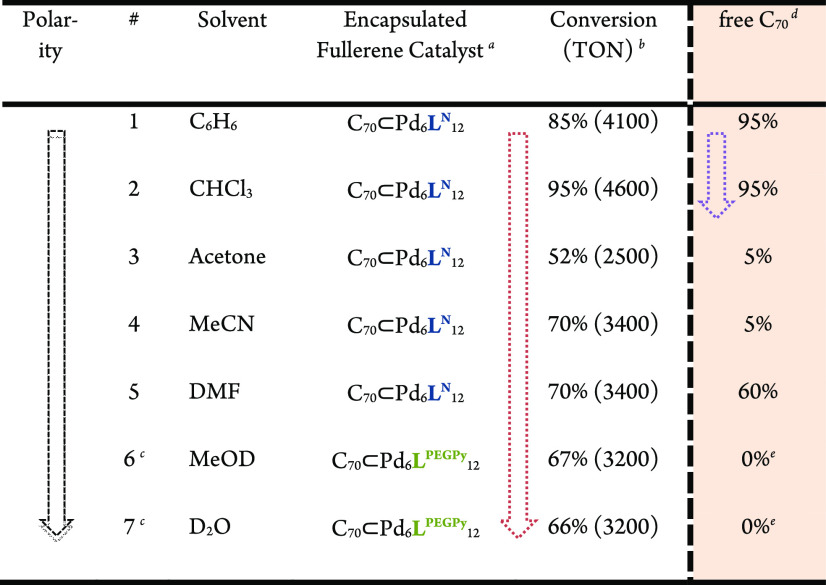
Oxidation of Organic ^1^O_2_ Acceptors by Light Induced Singlet Oxygen Formation in Different Media

a Standard condition: sphere, 4.16 nmol, substrate 20 μmol in 1 mL solvent, 4 h, room temperature; reactions performed in quartz containers located 2 cm away from a white LED light source.

b Conversion and turnover number (TON) based on nanosphere amount was determined by ^1^H NMR using mesitylene as internal standard.

c *N*-(*tert*-Butoxycarbonyl)-l-methionine (20 μmol) was used as substrate.

d C_70_ 16.6 nmol dissolved in 10 μL of toluene and 1 mL of cosolvent (described in the table).

e Free C_70_ was added as a solid.

After demonstrating that the C_70_ containing the [Pd_6_**L**^**N**^_12_] nanosphere displays a high productivity in light driven ^1^O_2_ in organic medium, we further expanded the scope by introducing the nanosphere–fullerene assemblies into more polar and aqueous media. For the application in water, the solubility of the nanosphere and the host–guest complex was achieved by using hydrophilic side chains attached to the outside of the sphere [Pd_6_**L**^**PEGPy**^_12_]. Boc-methionine, a well-known ^1^O_2_ acceptor was applied as the substrate in these polar solvents since anthracene is insufficiently soluble in water and polar solvents. C_70_⊂[Pd_6_**L**^**PEGPy**^_12_] showed good productivity in aqueous media (TON = 3200, Table 1, entry 7, solubility assessment S11), making it a suitable candidate for ^1^O_2_ generation in water, enhancing significantly the applicability scope of fullerenes. As free C_70_ does not dissolve, experiments using free C_70_ as catalysts resulted in no conversion at all.

### Substrate Scope and Soot Extract Photocatalytic ^1^O_2_ Formation

After having established the solvent compatibility of the C_70_⊂sphere complex, acetonitrile was chosen as standard solvent for productivity and scope investigation of all developed systems. Interestingly, under these conditions the [Pd_6_**L**^**N**^_12_] nanospheres themselves showed some catalytic productivity in the peroxidation of anthracene. Whereas [Pd_6_**L**^**O**^_12_] showed a marginal productivity (TON = 20), which is attributed to the background reaction, [Pd_6_**L**^**N**^_12_] showed to be a good photocatalyst for the peroxidation of anthracene (TON = 630). We attribute the catalytic productivity of [Pd_6_**L**^**N**^_12_] to the weak absorption of the nanosphere above 400 nm (Figure 5D), which is an excitation wavelength of the carbazole unit of the building block, which is part of the nanosphere (for comparison with other systems, see for example, refs (46−48)). [Pd_6_**L**^**O**^_12_] has no absorption above 400 nm, making the sphere itself less effective in ^1^O_2_ generation using white LED light.

In line with the better absorbance of C_70_ than C_60_ in the visible light, higher productivity was obtained using C_70_⊂[Pd_6_**L**^**N**^_12_] than C_60_⊂[Pd_6_**L**^**N**^_12_] (Table 2, entries 4 and 5). Also directly extracted fullerene from fullerene soot (as produced by arc vaporization)^49^ using [Pd_6_**L**^**N**^_12_] was applied in catalysis. Fullerene directly extracted from fullerene soot is economically preferred due to its large availability (for the procedure, see experimental section). The MS-analysis of C_60_/C_70_^soot^⊂[Pd_6_**L**^**N**^_12_] displayed a range of fullerene-sphere adducts (Figure S48). The major species were attributed to C_60_⊂[Pd_6_**L**^**N**^_12_], C_70_⊂[Pd_6_**L**^**N**^_12_], and to mixtures of both fullerenes associated with the nanosphere (see section S5 for details of soot extraction). The C_60_/C_70_^soot^⊂[Pd_6_**L**^**N**^_12_] complex displayed good catalytic productivity (TON = 1400), exceeding the performance of pure C_60_⊂[Pd_6_**L**^**N**^_12_]. Since C_70_ outperforms C_60_, the soot extract which is a mixture of both fullerenes outperforms pure C_60_⊂[Pd_6_**L**^**N**^_12_], but performs less well than pure C_70_⊂[Pd_6_**L**^**N**^_12_]. Interestingly, the C_60_/C_70_^soot^⊂[Pd_6_**L**^**N**^_12_] composite yields a different product than all other applied catalysts (Table 2, entry 6).

**Table 2:**
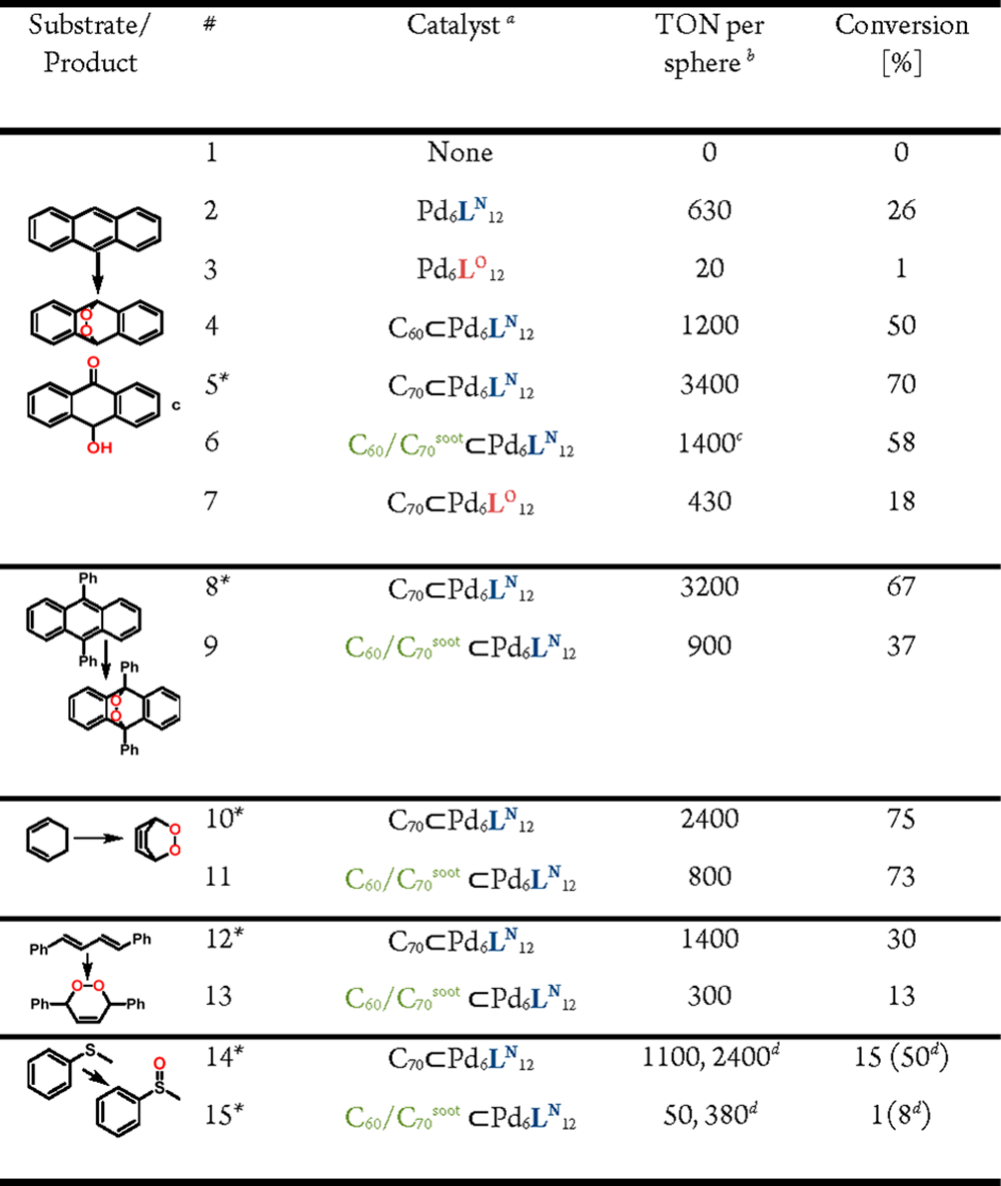
Oxidation of Organic Substrates by Light Induced Singlet Oxygen Formation

a Standard condition: Sphere 4.16 nmol, Substrate 10 μmol (* 20 μmol of substrate was used instead as full conversion was reached with 10 μmol)) in 1 mL MeCN-*d*_3_, 4 h, room temperature; reactions performed in quartz containers located 2 cm away from a white LED light source.

b Turnover number (TON) based on nanosphere amount was determined by ^1^H NMR using mesitylene as internal standard.

c 10-hydroxyanthracen-9(10H)-one was identified as the main product.

The dibenzofurane based structures C_70_⊂[Pd_6_**L**^**O**^_12_] performed less well than all other applied catalysts. This can be attributed to a lack of visible absorption of **L**^**O**^ and the lower amount of C_70_ bound to [Pd_6_**L**^**O**^_12_] (Table 2, entry 7).

Both C_70_⊂[Pd_6_**L**^**N**^_12_] and C_60_/C_70_^soot^⊂[Pd_6_**L**^**N**^_12_] were studied in a small scope of substrates. For diphenyl anthracene, C_60_/C_70_^soot^⊂[Pd_6_**L**^**N**^_12_] and C_70_⊂[Pd_6_**L**^**N**^_12_] displayed a similar activity as found for anthracene (Table 2, entries 8 and 9). Cyclohexadiene was converted slightly less efficiently, and it resulted in the formation of different products. The major species was identified as the expected peroxo species and minor amounts of aldehydes were formed as judged by ^1^H NMR spectra (Figure S69). Acyclic alkenes were oxidized by both C_70_⊂[Pd_6_**L**^**N**^_12_] and C_60_/C_70_^soot^⊂[Pd_6_**L**^**N**^_12_] with lower productivity compared to the previous substrates. Interestingly, also the challenging oxidation of thioanisole was possible using C_70_⊂[Pd_6_**L**^**N**^_12_] or C_60_/C_70_^soot^⊂[Pd_6_**L**^**N**^_12_] with good conversion after 8 h, whereas in the absence of nanospheres no conversion of the product was observed. In short, the fullerene containing Pd_6_L_12_ nanospheres are readily available and yield systems that are effective in ^1^O_2_ generation for the application in oxidation of aromatics, cyclic- and acyclic dienes, and thioethers with turnovers of 300–3400. The spheres can be easily separated from the desired products by either column chromatography or precipitation, making them useful candidates for application in organic synthesis.

### Catalytic Formation of ^1^O_2_ in Water and Buffer

**Scheme 1:**
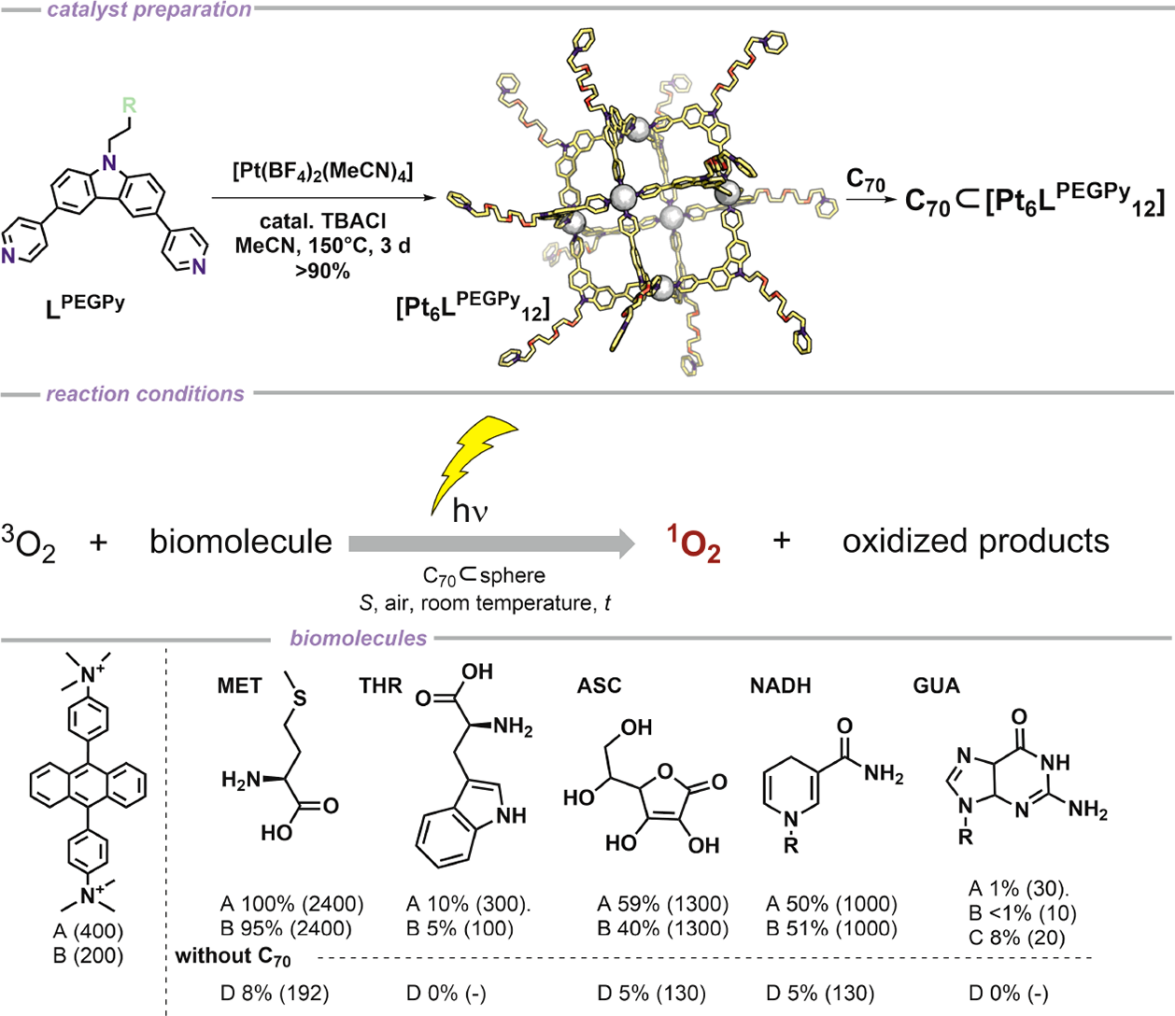
Oxidation of Biomolecules by Light Induced Singlet Oxygen Formation in Aqueous Media Top: [Pt_6_**L**^**PEGPy**^_12_] sphere formation procedure and C_70_ incorporation. Bottom: Standard reaction condition for oxidation of biomolecules using C_70_⊂[Pt_6_**L**^**PEGPy**^_12_]: (A) C_70_⊂[Pt_6_**L**^**PEGPy**^_12_] 4.16 nmol, substrate 10 μmol in 1 mL of D_2_O and 5 μL of DMSO (0.5%), 4 h, room temperature, white 11W LED. Deviation from standard reaction conditions: (B) reaction performed in 0.5 mL of D_2_O and 0.5 mL of (1 N) PBS_aq_; (C) C_70_⊂[Pt_6_**L**^**PEGPy**^_12_] 41.6 nmol. Turnover number (TON) and conversion were determined by ^1^H NMR using maleic acid as internal standard. (D) Empty [Pt_6_**L**^**PEGPy**^_12_] 4.16 nmol used as a catalyst in 0.5 mL of D_2_O and 0.5 mL of (1 N) PBS_aq_.

As mentioned in the introduction, a major ^1^O_2_ application field is photodynamic therapy. After supporting the effectiveness of our design to bind efficiently multiple fullerenes and displaying activity in ^1^O_2_ production using palladium-based spheres in organic or aqueous medium, we further expanded the applicability scope by introducing the nanospheres into biologically relevant conditions. For the application in biological medium, solubility in aqueous buffer and good stability against biologically relevant molecules (such as chloride, amines, and acids) is required. As demonstrated before, palladium based nanospheres were shown to be not sufficiently stable under these circumstances.^50^ Generally, the platinum counterparts of M_*n*_L_2*n*_ nanospheres exhibit improved stability under biologically relevant conditions and have fluorescent properties.^50−53^ Platinum-based sphere formation was performed according to reported protocols.^41,50,53,54^ The nanosphere was prepared by mixing 0.6 equiv [Pt(BF_4_)_2_(MeCN)_4_], 7 mol % TBACl as catalyst, and 1 equiv **L**^**PEGPy**^ in acetonitrile at 150 °C for 72 h (Scheme 1). After this period a clear downfield shift of the pyridine protons together with a lower diffusion coefficient in DOSY NMR supported the formation of the desired [Pt_6_**L**^**PEGPy**^_12_] nanosphere (Figures S90–S93). A detailed analysis of the recorded MS spectrum revealed a good selectivity for the formation of [Pt_6_**L**^**PEGPy**^_12_]. The major species found in the MS analysis were attributed to different charge states of [Pt_6_**L**^**PEGPy**^_12_]^*x*+^ for *x* = 4–12 (Figure S93). Minor amounts of [Pt_5_**L**^**PEGPy**^_10_] were also detected, giving an overall 90% selectivity for the desired [Pt_6_**L**^**PEGPy**^_12_] nanosphere based on MS analysis (for quantification see ref (54)). The nanosphere showed similar binding features for C_70_ as the corresponding palladium counterpart, making it a suitable candidate for further investigation (Figure S95).

First, the stability of [Pd_6_**L**^**PEGPy**^_12_] and [Pt_6_**L**^**PEGPy**^_12_] was briefly studied. In agreement with previous reports, [Pd_6_**L**^**PEGPy**^_12_] decomposed quickly after being exposed to NaCl_aq_ as evidenced by formation of a precipitate and the disappearance of the sphere associated signals in ^1^H NMR. [Pt_6_**L**^**PEGPy**^_12_] remained in solution after 10 h at 37 °C. No precipitate was formed, and all signals associated with the nanosphere remained the same in the ^1^H NMR spectrum during the course of the experiment (Figure S97). [Pt_6_**L**^**PEGPy**^_12_] displays emission around 450 nm when excited at 380 nm (Figure S98). In agreement to previous investigations on fullerene binding self-assemblies, the fluorescence is quenched to a certain degree when fullerene is bound (Figure S98). With the promising fluorescence and stability of [Pt_6_**L**^**PEGPy**^_12_], its application in ^1^O_2_ generation in aqueous media was studied. We decided to investigate the ^1^O_2_ formation ability by employing a variety of well-known ^1^O_2_ quenchers which can be found in living cells under two different conditions. Previous investigations into ^1^O_2_ reactivity in living cells identified the major absorbents being proteins (40%), ascorbate (15%), water (7%), and NADPH (1%).^55^ Therefore, we studied some of the most common quenchers using our ^1^O_2_ generating assembly. We investigated the productivity of C_70_⊂[Pt_6_**L**^**PEGPy**^_12_] using a white 11 W LED as source in (A) D_2_O, (B) PBS buffered water (Scheme 1).

The generation of ^1^O_2_ in aqueous medium and in buffered solution upon irradiation with white LED light is supported by the oxidation of substituted anthracene (Scheme 1). Next, different types of amino acids, as model substrates for proteins, were applied as substrates for light driven oxidation. C_70_⊂Pt_6_**L**^**PEGPy**^_12_ promotes oxidation of methionine and tryptophan in D_2_O using white LED light (Scheme 1, condition A, TON = 100–2400). Similar results are also obtained when PBS buffered water is used as the reaction medium (Scheme 1, condition B). Because oxidation is not promoted when no fullerene-carrier is applied, this observation supports a good stability of complexes in the presence of chloride and amines. In comparison to the fullerene loaded nanosphere, control experiments with the empty [Pt_6_**L**^**PEGPy**^_12_] nanosphere show also formation of ^1^O_2_ but to a much lesser extent (Scheme 1; condition D, for example, 8% for methionine in the absence of fullerene (D) and 95% in the presence (B)). Common biologically relevant reductants such as NADH and ascorbate are oxidized successfully with similar turnovers (around 1000) using either of the two reaction conditions. Finally, also guanosine was briefly studied. Using white light in water (condition A) or in PBS (condition B) affords little conversion of the starting material (∼1%). Increasing the catalyst loading by 10-fold (condition C) increases the conversion accordingly to 8%. Whereas the conversions are not very high, it is important to mention that the individual nanospheres reach a turnover of 10–30 in the oxidation of guanosine, which makes them potentially efficient candidates for damaging DNA.

Although a brief study into biologically relevant application, we showed that C_70_⊂[Pt_6_**L**^**PEGPy**^_12_] is productive in ^1^O_2_ generation in aqueous and buffered solutions. Different types of prominent ^1^O_2_ acceptors such as amino acids and reducing agents were oxidized in PBS using white LED light, making [Pt_6_**L**^**PEGPy**^_12_] a potential vehicle for fullerene application for further investigations into the biomedicinal fields.

## Conclusion

We introduced new M_6_L_12_ nanospheres that can bind fullerenes to the windows of these cubic self-assembled structures. This is a new design principle for fullerene binding as previous structures allowed binding to the interior space. Utilizing the window space allowed the M_6_L_12_ spheres to carry up to four fullerenes. The M_6_L_12_ nanospheres rely on a simple ligand design and are readily available from commercial materials. The Pd_6_L_12_ nanospheres were shown to bind fullerene after extraction from soot. The fullerenes containing Pd_6_L_12_ nanospheres are productive in light driven ^1^O_2_ formation which can be used for the oxidation of a variety of organic compounds in organic solvents of different polarity. Exploiting the easy derivatization of the building blocks used for Pd_6_L_12_ nanospheres formation, allowed the preparation of a water-soluble fullerene-containing nanospheres. This [Pd_6_**L**^**PEGPy**^_12_] nanosphere is active in the generation of ^1^O_2_ in water, and as such can be used in catalytic oxidation. The biological relevance of the application of C_70_⊂[Pt_6_**L**^**PEGPy**^_12_] in ^1^O_2_ generation in aqueous and buffered solutions is briefly demonstrated by the light driven oxidation of some amino acids and reducing agents in PBS using white LED light. This makes [Pt_6_**L**^**PEGPy**^_12_] a potential vehicle for fullerene application in the biomedicinal fields, which deserves further investigation. The general design principle and the ease of derivatization of the building blocks for cage formation provide a strong basis for the design of systems suitable for PDT, an avenue that is currently be further explored. This work provides new design strategies for the development of efficient and active fullerene binding coordination-based M_6_L_12_ nanospheres.

## Supporting Information Available

NMR spectra for all compounds, mass spectrometry, synthetic and additional experimental details and methods (PDF)
