# Supplementary material for: M6L12 Nanospheres with Multiple C70 Binding Sites for 1O2 Formation in Organic and Aqueous Media
Source: J Am Chem Soc. 2022 Aug 17;144(34):15633–42. doi: 10.1021/jacs.2c05507 (PMC9437924; doi:10.1021/jacs.2c05507)
Supplement: Supplementary file 1 — ja2c05507_si_001.pdf [file ja2c05507_si_001.pdf]

Supporting information for

M_6L_{12} nanospheres with multiple C_{70} binding sites for $^1O_2$ formation in organic and aqueous media

Eduard. O. Bobylev,[†] D. A. Poole III,[†] Bas de Bruin[†] and Joost N. H. Reek[†]

[†] van 't Hoff Institute for Molecular Sciences, University of Amsterdam, Science Park 904, 1098 XH Amsterdam, the Netherlands.

List of content:

S1:	Synthesis of building blocks	3
S2:	Synthesis of spheres	6
S3:	Fullerene binding, general considerations	12
S4:	Fullerene Binding $Pd_6L^O{}_{12}$	17
S5:	Fullerene Binding $Pd_6L^N{}_{12}$	24
S6:	Fullerene Binding $Pd_6L^{PEGPy}{}_{12}$	34
S7:	Catalytic $^1O_2$ formation	37
S8:	MS analysis evaluation	41
S9:	Fullerene localization	44
S10:	Catalytic performance discussion	48
S11:	Water-solubility	52
S12:	Catalysis in water and buffered water	53
S12:	References	62

Materials and methods

General procedures: All synthetic procedures were carried out under a nitrogen atmosphere using standard Schlenk techniques. All commercially available chemicals were used as received without further purification. Solvents used for synthesis were dried, distilled and degassed with the most suitable method. Column chromatography was performed open to air using solvents as received.

Cryospray-ionization MS (CSI-MS): Mass spectra were collected on a HR-ToF Bruker Daltonik GmbH (Bremen, Germany) Impact II, an ESI-ToF MS capable of resolution of at least 40000 FWHM, which was coupled to a Bruker cryo-spray unit. Detection was in positive-ion mode and the source voltage was between 4 and 6 kV. The sample was introduced with a syringe pump at a flow rate of 18 ul/hr. The drying gas (N_2) was held at 40°C and the spray gas was held at 60°C. The machine was calibrated prior to every experiment via direct infusion of a TFA-Na solution, which provided a m/z range of singly charged peaks up to 3500 Da in both ion modes. Software acquisition Compass 2.0 for Otof series. Software processing m- mass.

Electrochemistry: Cyclic voltammetry was performed on 1 mM solution of analyte using 0.1 M tetrabutylammonium hexafluorophosphate as supporting electrolyte. The voltammograms were recorded using a PG-STAT302N potentiostat at glassy carbon disk electrode (1 mm diameter). A platinum coil was used as auxiliary electrode and a leak free silver electrode (inner compartment 3 M KCl/Ag) as reference electrode. The experiments were performed in dry degassed MeCN.

UV/VIS: Measurements were performed on a Shimadzu UV-2600, 240V IVDD UV-VIS spectrophotometer.

Catalytic $^1O_2$ formation: All reactions were performed in open quarz tubes located 2 cm away from the white LED light source (Zahner, TLS3, 100 mW cm^{-2}), with the spectral output given below (adopted from [1]):

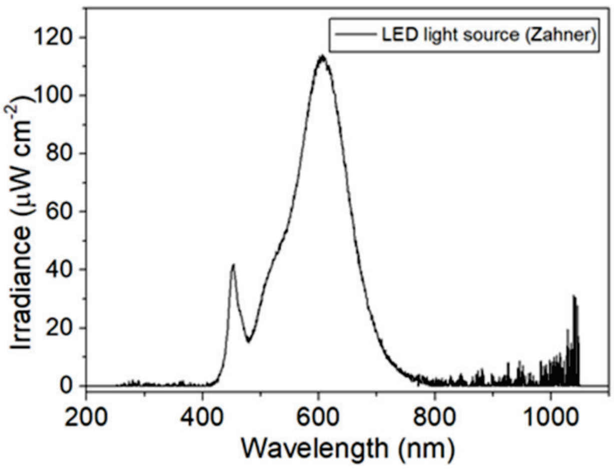

Fluorescence: Spectra were recorded on a Fluorolog Jobin Yvon-SPEX together with their corresponding UV/Vis spectra (Shimadzu UV-2700 Spectrometer).

Synthesis of building blocks (S1)

L^O was synthesized according to literature.[2]

Synthesis of $\mathbf{L^{acetylO}}$

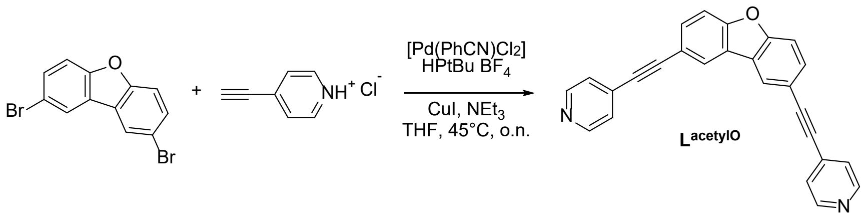

Scheme S1. Synthetic route for the building block $\mathbf{L^{acetylO}}$.

The building block was synthesized according to a slightly modified literature procedure: HP^tBuBF_4 (54 mg, 0.18 mmol, 0.12 eq.) and $Pd(PhCN)_2Cl_2$ (35 mg, 0.09 mmol, 0.06 eq.) were dissolved in a mixture of degassed THF (20 mL) and trimethylamine (20 mL). The solution was stirred 20 min at room temperature before 2,6-dibromo-dibenzofurane (0.5 g, 1.53 mmol, 1 eq.) and 4-ethynylpyridine hydrochloride (0.54 g, 3.83 mmol, 2.5 eq.) were added. The solution was gently heated up to 45°C and copper(I) iodide (12 mg, 0.06 mmol) was added. This mixture was stirred at 45 °C for 24 h under nitrogen atmosphere. The reaction mixture was poured into 400 mL ethylacetate, filtrated over a pad of sand and evaporated in *vacuo*. The crude product was purified by column chromatography on silica gel (DCM/MeOH = 99/1) to give $\mathbf{L^{acetylO}}$ (0.27g, 48%) as a white powder. ^{1}H NMR (300 MHz, Chloroform-*d*) δ 8.67 (s, 4H), 8.23 – 8.10 (m, 2H), 7.76 – 7.65 (m, 2H), 7.59 (d, *J* = 8.5 Hz, 2H), 7.52 – 7.38 (m, 4H).

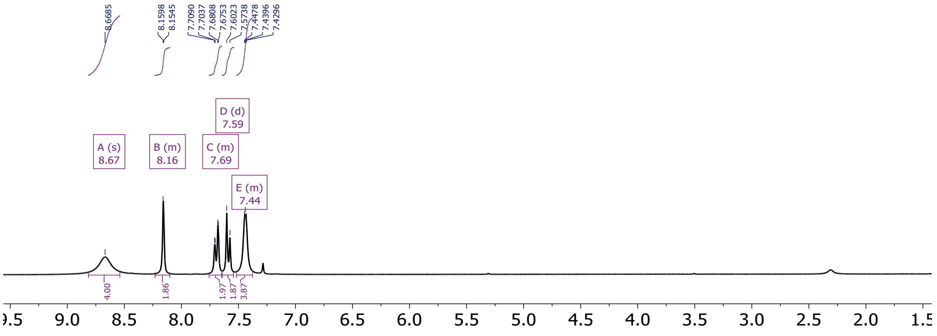

Figure S1. $\mathbf{L^{acetylO}}$ building block, ^{1}H NMR in $CDCl_3$.

Synthesis of $\mathbf{L^N}$

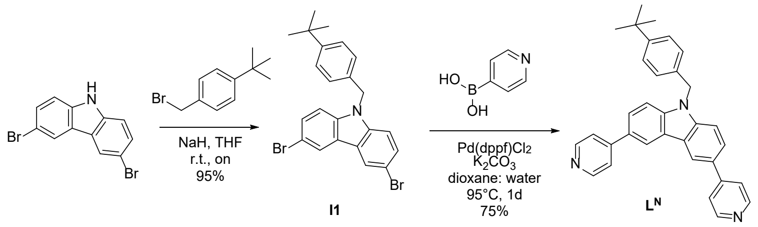

Scheme S2. Synthetic route for the rigid building block $\mathbf{L^N}$.

I1: To a solution of dibromo carbazole (2 g, 6.2 mmol, 1 eq.) in 20 mL dry THF, NaH (60% in paraffin oil, 370 mg, 9.2 mmol, 1.5 eq.) was added slowly. The resulting suspension was stirred for 1 h at room temperature before 4-tert-buthylbenzyl bromide (1.4 g, 6.2 mmol, 1 eq.) was added. The solution was stirred over night at room temperature, poured into water and the product extracted into EtOAc (4x 100 mL). The crude white solid material (2.8 g, 95%) was sufficiently pure for the next step as judged by ^{1}H-NMR. ^{1}H NMR (300 MHz, Chloroform-*d*) δ 8.19 (d, *J* = 1.9 Hz, 2H), 7.54 (dd, *J* = 8.7, 1.9 Hz, 2H), 7.34 – 7.23 (m, 4H), 7.07 – 6.98 (m, 2H), 5.46 (s, 2H), 1.28 (s, 9H).

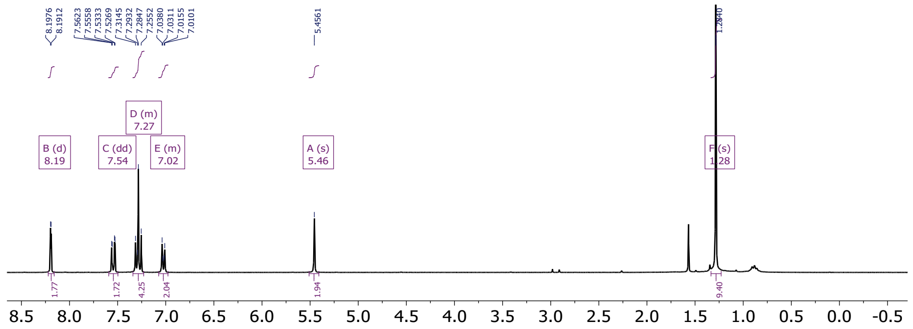

Figure S2. I1, ^{1}H NMR in $CDCl_3$.

L^N: **L^N** was synthesized in a similar fashion to **L^O**: **I1** (1.5 g, 3 mmol, 1eq.), 4-boronic acid pyridine (1.5 g, 12.22 mmol, 4 eq.) and K_2CO_3 (9 g, 60 mmol, 20. eq.) were suspended in 80 mL dioxane and 16 mL water. Nitrogen was bubbled into the solution for 20 min before Pd(dppf)Cl_2 (224 mg, 0.3 mmol, 0.1 eq.) was added. The mixture was heated to 95°C for 48h. After cooling the mixture to room temperature, the volatiles were removed under reduced pressure. The product was extracted into 300 mL EtOAc and the organic phase was washed with 3x1M $NaOH_{aq}$. The organic phase was dried with Na_2SO_4 and the volatiles were removed under reduced pressure. The crude material was purified by column chromatography (SiO_2, 3%MeOH: DCM) to afford **L^N** (1.1 g, 75%) as a white solid. ^{1}H NMR (300 MHz, DMSO-d_6) δ 8.90 (d, *J* = 1.8 Hz, 2H), 8.66 (d, *J* = 5.5 Hz, 4H), 7.97 (dd, *J* = 8.6, 1.8 Hz, 2H), 7.90 – 7.83 (m, 4H), 7.82 (d, *J* = 8.7 Hz, 2H), 7.30 (d, *J* = 8.0 Hz, 2H), 7.14 (d, *J* = 8.0 Hz, 2H), 5.73 (s, 2H), 1.20 (s, 9H). ^{13}C NMR (75 MHz, DMSO) δ 150.61, 150.26, 148.10, 141.77, 134.91, 128.89, 126.94, 125.87, 125.58, 123.64, 121.45, 119.92, 111.09, 45.98, 40.82, 40.55, 40.27, 39.99, 39.71, 39.43, 39.16, 34.64, 31.50. HR-ESI-MS, calculated for $C_{33}H_{30}N_3$ 468.2435, obtained 468.2356.

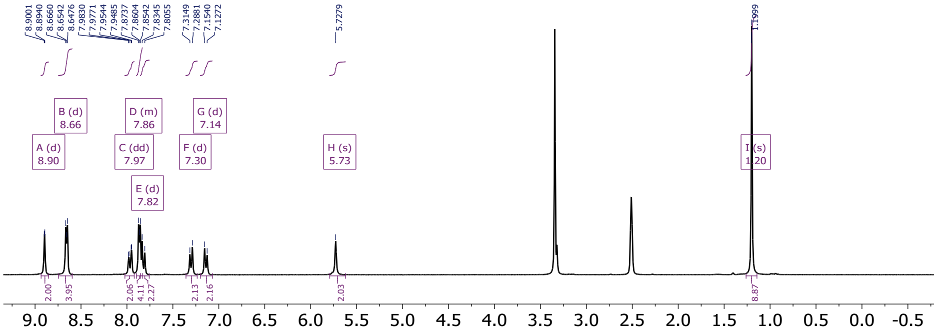

Figure S3. L^N building block, ^{1}H NMR in dmso-d_6.

<u>Synthesis of **L^{PEGPy}**</u>

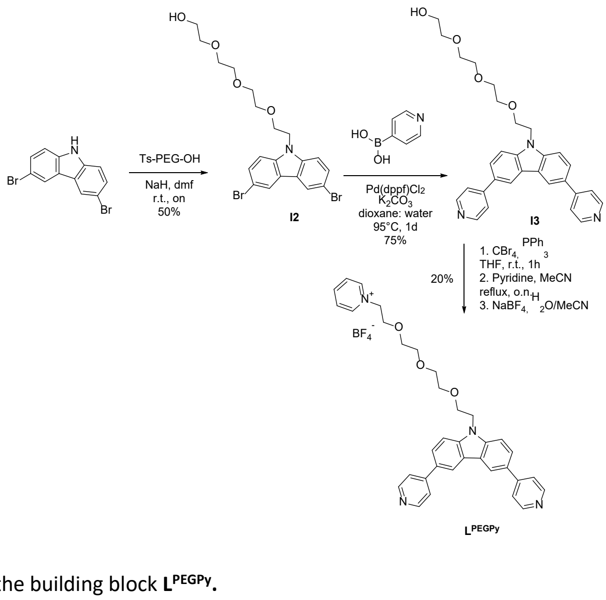

Scheme S3. Synthetic route for the building block **L^{PEGPy}.**

I2: To a solution of dibromo carbazole (2 g, 6.2 mmol, 1 eq.) in 15 mL dry dmf, NaH (60% in paraffin oil, 370 mg, 9.2 mmol, 1.5 eq.) was added slowly. The resulting suspension was stirred for 1 h at room temperature before tosylPEG (2.2 g, 6.2 mmol, 1 eq.) was added. The solution was stirred over night at room temperature, poured into water and the product extracted into EtOAc (4x 100 mL). The crude material was purified by column chromatography (SiO_2, 1%MeOH: DCM) to afford **I2** (1.5 g, 50%) as a colorless oil. ^{1}H NMR (300 MHz, Chloroform-*d*) δ 8.14 (d, *J* = 1.9 Hz, 2H), 7.56 (dd, *J* = 8.7, 1.9 Hz, 2H), 7.37 (d, *J* = 8.7 Hz, 2H), 4.47 (t, *J* = 5.7 Hz, 2H), 3.85 (t, *J* = 5.7 Hz, 2H), 3.76 – 3.69 (m, 2H), 3.69 – 3.45 (m, 12H). ^{13}C NMR (75 MHz, $CDCl_3$) δ 162.55, 139.57, 129.02, 123.51, 123.11, 112.18, 110.84, 77.45, 77.03, 76.60, 72.38, 70.93, 70.60, 70.52, 70.20, 69.32, 61.69, 43.52, 36.51.

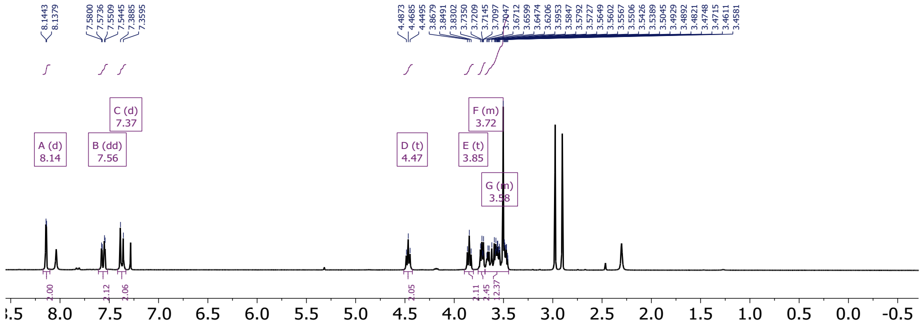

Figure S4. I2, ^{1}H NMR in $CDCl_3$.

I3: **I3** was synthesized in a similar fashion to **L**$^{\mathbf{N}}$ and **L**$^{\mathbf{O}}$: **I2** (1.5 g, 3 mmol, 1eq.), 4-boronic acid pyridine (1.5 g, 12.22 mmol, 4 eq.) and K_2CO_3 (9 g, 60 mmol, 20. eq.) were suspended in 80 mL dioxane and 16 mL water. Nitrogen was bubbled into the solution for 20 min before Pd(dppf)Cl_2 (224 mg, 0.3 mmol, 0.1 eq.) was added. The mixture was heated to 95°C for 48h. After cooling the mixture to room temperature, the volatiles were removed under reduced pressure. The product was extracted into 300 mL EtOAc and the organic phase was washed with 3x1M $NaOH_{aq}$. The organic phase was dried with Na_2SO_4 and the volatiles were removed under reduced pressure. The crude material was purified by column chromatography (SiO_2, 3%MeOH: DCM) to afford **I3** (1.1 g, 75%) as an off-white solid. ^{1}H NMR (300 MHz, Chloroform-*d*) δ 8.74 (s, 4H), 8.47 (d, *J* = 1.7 Hz, 2H), 7.83 (dd, *J* = 8.5, 1.7 Hz, 2H), 7.71 (d, *J* = 5.0 Hz, 4H), 7.64 (d, *J* = 8.5 Hz, 2H), 4.61 (t, *J* = 5.7 Hz, 2H), 3.95 (t, *J* = 5.7 Hz, 2H), 3.77 – 3.48 (m, 15H). ^{13}C NMR (75 MHz, $CDCl_3$) δ 149.79, 149.26, 141.77, 129.48, 125.32, 123.58, 121.96, 119.05, 110.12, 100.21, 77.45, 77.03, 76.60, 72.40, 70.97, 70.65, 70.56, 70.24, 69.40, 67.09, 61.67, 60.39, 43.63, 21.06, 14.21.

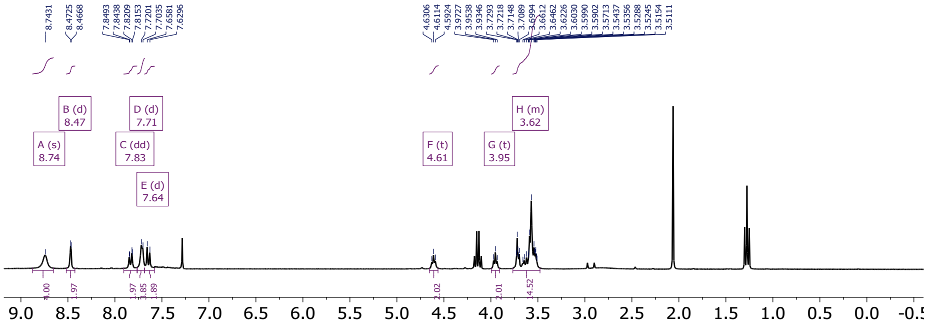

Figure S5. I3, ^{1}H NMR in $CDCl_3$.

L$^{\mathbf{PEGPy}}$: To a solution of **I3** (1.7 g, 3 mmol) in 40 mL dry THF, CBr_4 (1.25 g, 3.9 mmol, 1.3 eq.) was added. Then, PPh_3 (1 g, 3.9 mmol, 1.3 eq.) was added slowly in four portions. The resulting solution was stirred for 1h at room temperature. The volatiles were evaporated under reduced pressure. The brominated intermediate was suspended in EtOAc (100 mL) and $NaHCO_{3aq}$ (100 mL). The product was extracted into EtOAc (3 x 100 mL). All volatiles were removed under reduced pressure to yield the brominated intermediate. Due to limited stability, the intermediate was used for the next step without further purification. The intermediate was dissolved in 50 mL dry MeCN and 15 mL pyridine were added before the solution was refluxed overnight. The volatiles were removed under reduced pressure. The crude material was dissolved in 20 mL MeCN and $NaBF_4$ (10g in 20 mL H_2O) was added. The solvent was removed under reduced pressure. The solid crude material was extracted with EtOAc under sonication (3 x 50 mL). The volatiles were removed under reduced pressure and the crude material was purified by column chromatography (SiO_2, 1%MeOH: DCM to 25%MeOH: DCM) to afford **L**$^{\mathbf{PEGPy}}$ (1.1 g, 75%) as an orange oil. ^{1}H NMR (300 MHz, DMSO-d_6) δ 8.92 – 8.86 (m, 2H), 8.84 (d, *J* = 1.8 Hz, 2H), 8.71 – 8.63 (m, 4H), 8.52 – 8.43 (m, 1H), 8.07 – 8.01 (m, 1H), 8.01 – 7.93 (m, 2H), 7.92 – 7.84 (m, 4H), 7.79 (d, *J* = 8.6 Hz, 2H), 4.65 (dt, *J* = 9.6, 5.0 Hz, 4H), 4.10 (q, *J* = 5.3 Hz, 2H), 3.86 (t, *J* = 5.2 Hz, 2H), 3.78 (t, *J* = 4.9 Hz, 2H), 3.45 (dd, *J* = 5.9, 3.6 Hz, 2H), 3.42 – 3.23 (m, 11H), 3.18 (d, *J* = 4.6 Hz, 4H). ^{13}C NMR (75 MHz, DMSO) δ 150.61, 148.10, 145.98, 145.48, 141.91, 128.58, 128.01,

125.23, 123.46, 121.39, 119.68, 111.20, 70.48, 70.06, 69.95, 69.92, 69.35, 68.92, 60.63, 49.06, 43.46, 40.83, 40.55, 40.27, 39.99, 39.84, 39.71, 39.43, 39.16. HR-ESI-MS, calculated for $C_{35}H_{35}N_4O_3$ 559.2704, obtained 559.3056.

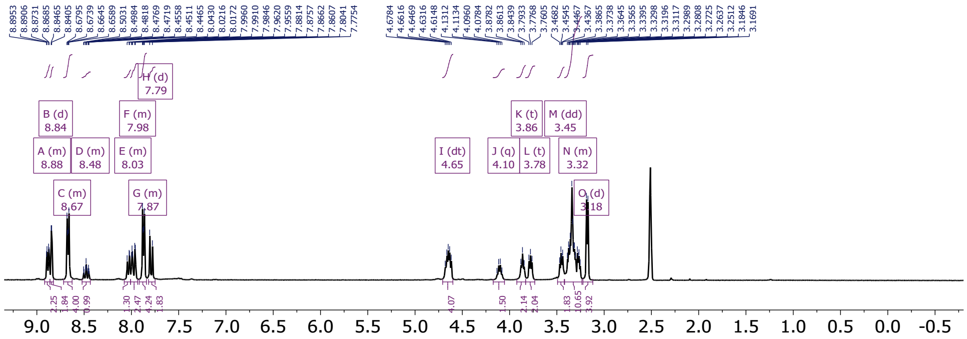

Figure S6. L^{PEGPy} building block, ^1H NMR in dmso-d_6.

Sphere Synthesis (S2)

$[Pd_6(\mathbf{L^O})_{12}]^{12+}(BF_4^-)_{12}$ was synthesized according to literature.[2]

$[Pd_6(\mathbf{L^{acetylO}})_{12}]^{12+}(BF_4^-)_{12}$

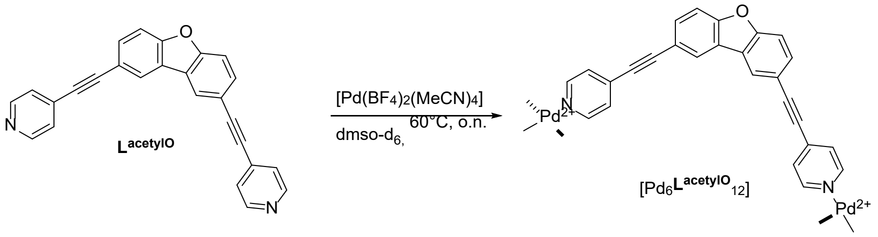

Scheme S4. Synthetic route for $Pd_6(\mathbf{L^{acetylO}})_{12}$.

The self-assembly was prepared according to literature procedure. All spectroscopic features were in line with reported values. Briefly: To a solution of $\mathbf{L^{acetylO}}$ (3.7 mg, 10 µmol, 1 eq.) in 1 ml dmso-d_6, $[Pd(BF_4)_2(MeCN)_4]$ (2.44 mg, 5.5 µmol, 0.55 eq.) was added and the resulting solution stirred at 60°C overnight to yield a stock solution of the desired cage in dmso (>95% as judged by NMR and MS). ^1H NMR (300 MHz, DMSO-d_6) δ 9.25 (d, J = 6.1 Hz, 4H), 8.57 (d, J = 1.4 Hz, 2H), 7.90 (d, J = 7.2 Hz, 6H), 7.82 (d, J = 9.1 Hz, 2H).

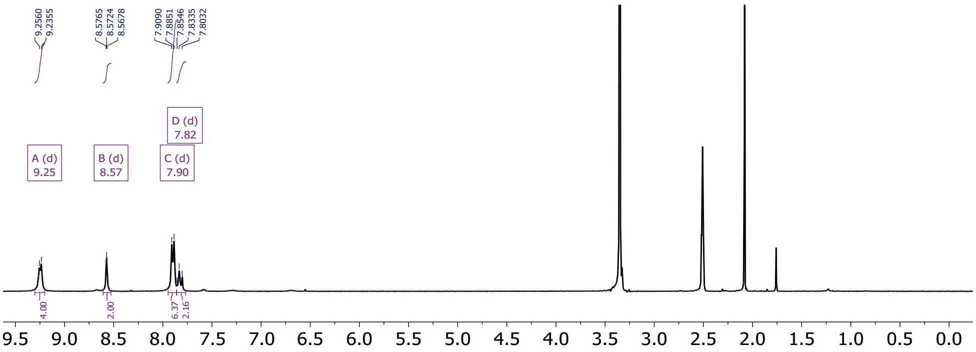

Figure S7. $[Pd_6(\mathbf{L^{acetylO}})_{12}]$ assembly, ^1H NMR in dmso-d_6.

$[Pd_6(\mathbf{L^N})_{12}]^{12+}(BF_4^-)_{12}$

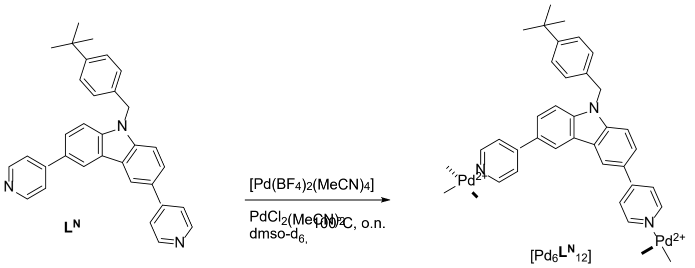

Scheme S5. Synthetic route for $Pd_6(\mathbf{L^N})_{12}$.

To a solution of $\mathbf{L^N}$ (4.76 mg, 10 µmol, 1 eq.) in 1 mL dmso-d$_6$, $[Pd(BF_4)_2(MeCN)_4]$ (2.44 mg, 5.5 µmol, 0.55 eq.) and $PdCl_2MeCN_2$ (0.1 mg) were added. The solution was then stirred at 100°C for 1d to yield a stock solution of the desired cage in dmso (>95% as judged by NMR and MS). ^{1}H NMR (400 MHz, DMSO-d_6) δ 9.33 (s, 4H), 9.10 (s, 3H), 8.33 (d, J = 6.1 Hz, 6H), 8.13 (d, J = 8.4 Hz, 3H), 7.81 (d, J = 8.6 Hz, 3H), 7.20 (d, J = 8.2 Hz, 3H), 7.05 (d, J = 8.3 Hz, 3H), 5.66 (s, 3H), 1.10 (s, 12H). ^{13}C NMR (101 MHz, DMSO) δ 171.88, 151.42, 150.81, 150.38, 142.93, 134.49, 126.94, 125.83, 125.58, 123.69, 123.27, 120.36, 118.54, 111.68, 99.99, 55.38, 34.58, 31.50, 31.40, 22.98, 1.62.

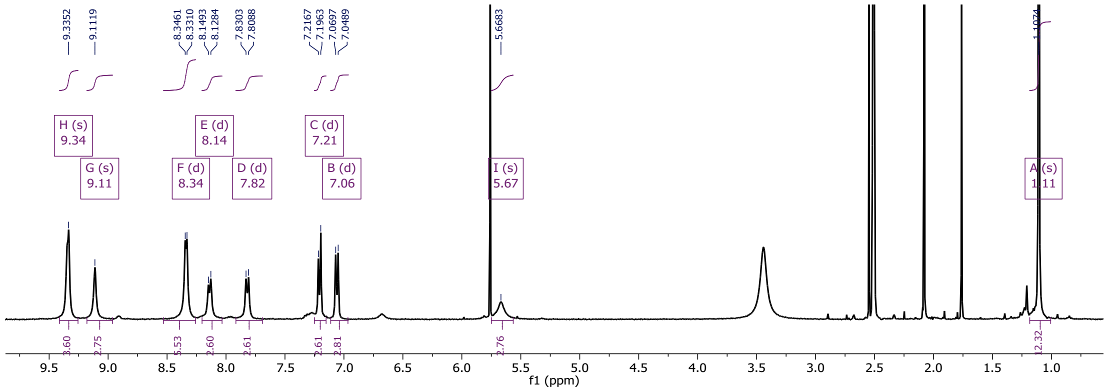

Figure S8. $[Pd_6(\mathbf{L^N})_{12}]$ assembly, ^{1}H NMR in dmso-d$_6$.

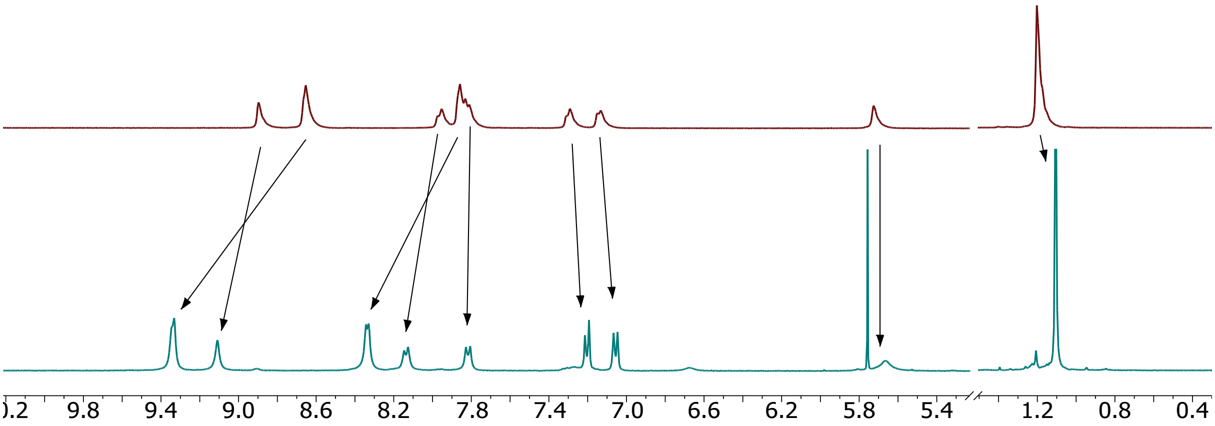

Figure S9. $[Pd_6(\mathbf{L^N})_{12}]$ assembly (bottom) and free building block (top), ^{1}H NMR in dmso-d$_6$.

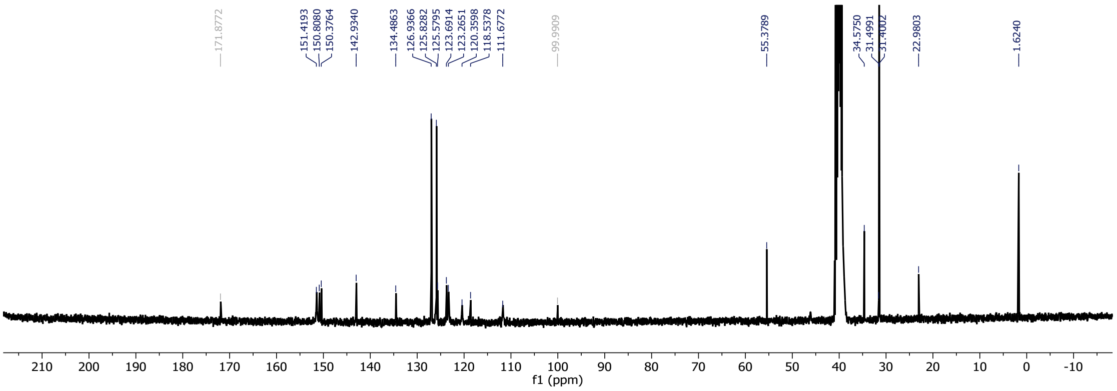

Figure S10. $[Pd_6(\mathbf{L^N})_{12}]$ assembly, ^{13}C NMR in dmso-d_6.

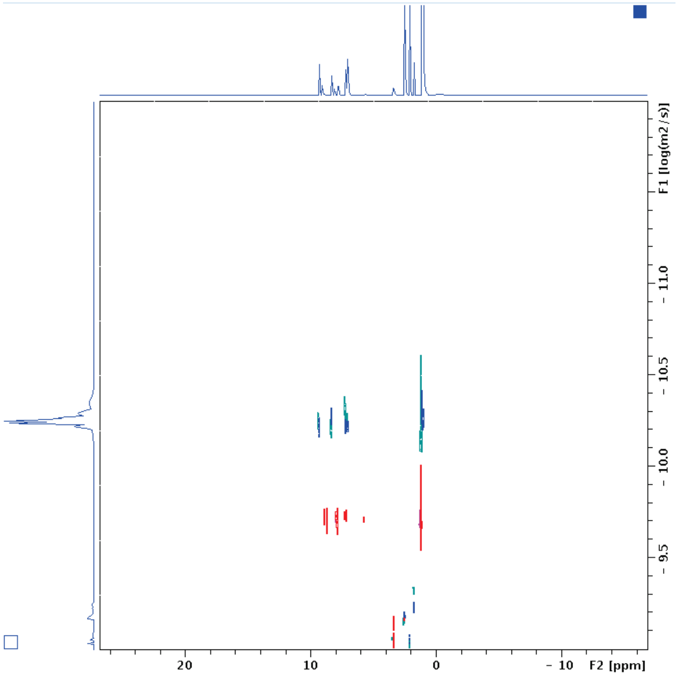

Figure S11. Overlay of $[Pd_6(\mathbf{L^N})_{12}]$ assembly (blue) and free building block (red), DOSY NMR of both recorded in dmso-d_6 at 300K.

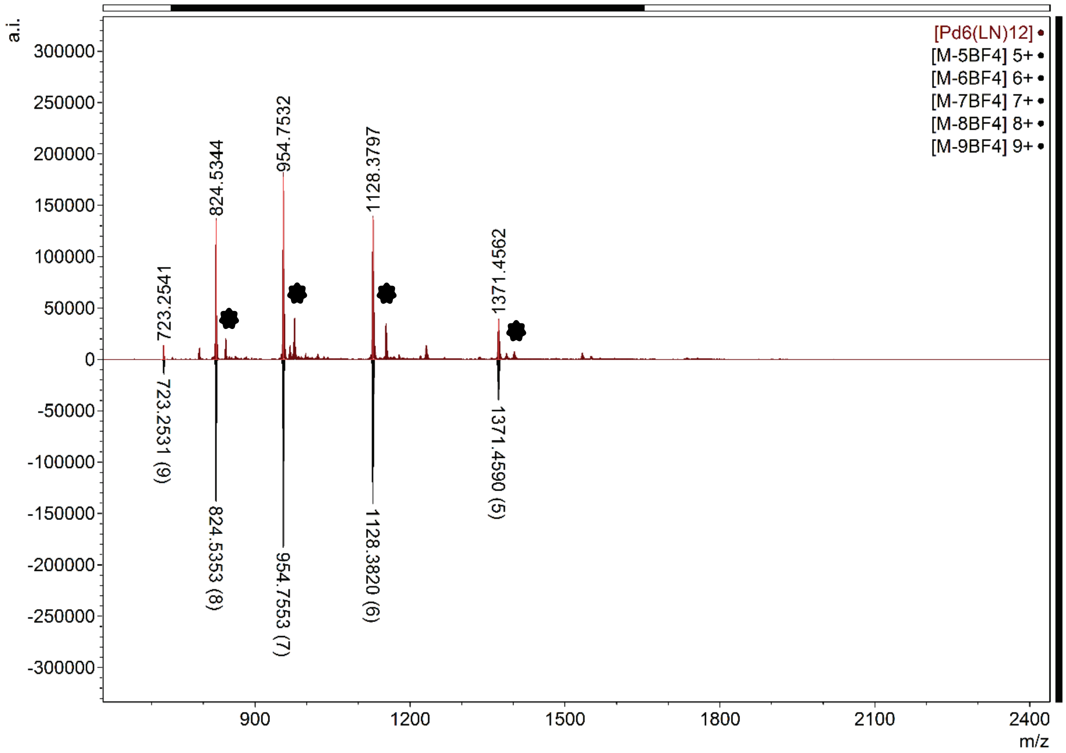

Figure S12. Full ESI-MS spectra of $[Pd_6(\mathbf{L^N})_{12}]^{12+}(BF_4^-)_{12}$. Below the simulated spectra, above obtained spectra. (Signals marked with a * represent cage signals together with solvent, e.g. 2 dmso + cage or 1 dmso 4 water).

Table S13. Zoom into ESI-MS spectra of $[Pd_6(\mathbf{L^N})_{12}]^{x+}(BF_4^-)_{12-x}$ (below the simulated spectra, above obtained spectra).

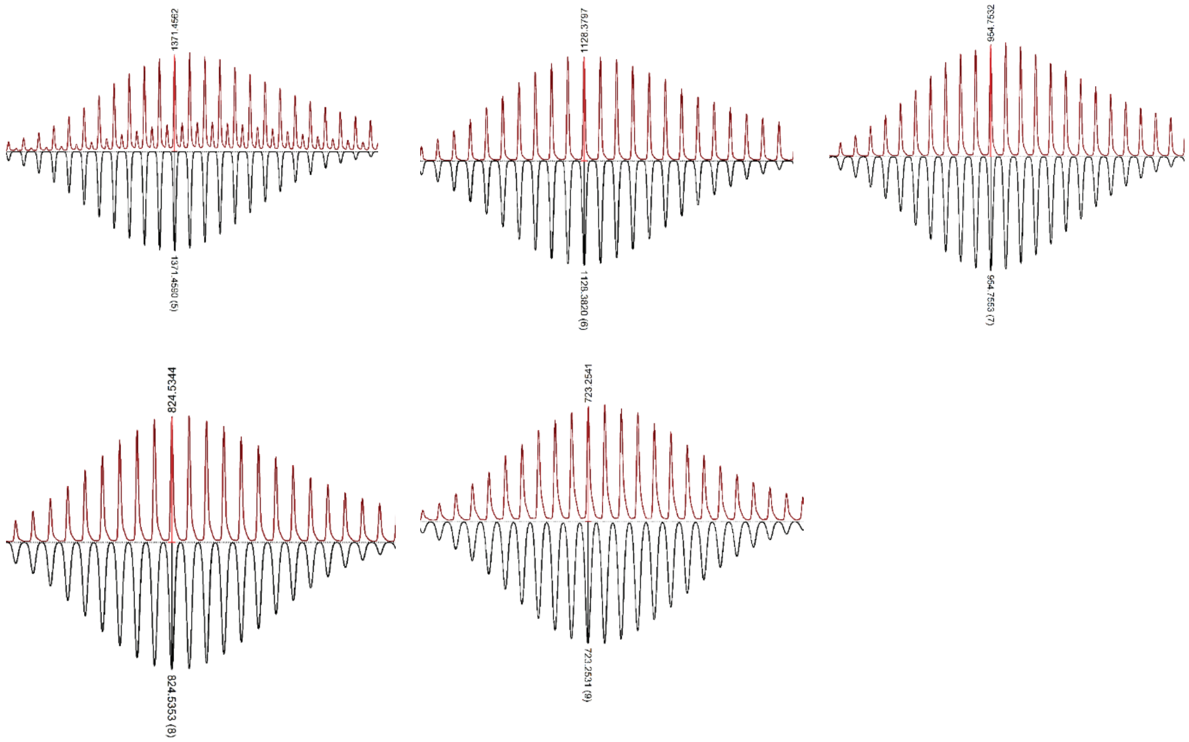

[$Pd_6(\mathbf{L}^{PEGPy})_{12}]^{24+}(BF_4^-)_{24}$

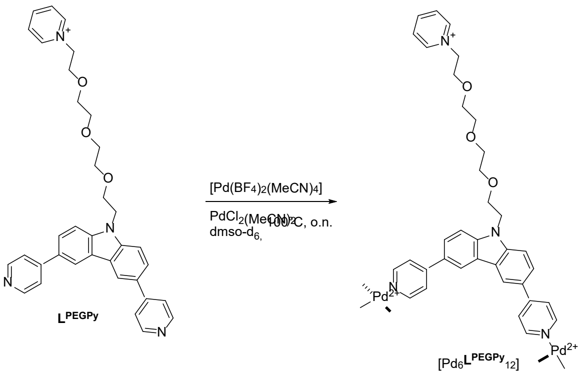

Scheme S6. Synthetic route for $Pd_6(\mathbf{L}^{PEGPy})_{12}$.

To a solution of $\mathbf{L}^{PEGPy}$ (6.46 mg, 10 µmol, 1 eq.) in 1 mL dmso-d$_6$, $[Pd(BF_4)_2(MeCN)_4]$ (2.44 mg, 5.5 µmol, 0.55 eq.) and $PdCl_2MeCN_2$ (0.1 mg) were added. The solution was then stirred at 100°C for 1d to yield a stock solution of the desired cage in dmso (>95% as judged by NMR and MS). ^{1}H NMR (300 MHz, DMSO-d_6) δ 9.36 (s, 4H), 9.07 (s, 2H), 8.89 – 8.82 (m, 2H), 8.43 (td, *J* = 7.8, 1.4 Hz, 1H), 8.40 – 8.32 (m, 5H), 8.16 (d, *J* = 8.1 Hz, 2H), 8.05 – 7.93 (m, 2H), 7.76 (d, *J* = 8.2 Hz, 2H), 4.61 (t, *J* = 4.9 Hz, 2H), 3.76 (d, *J* = 6.0 Hz, 4H), 3.40 (d, *J* = 16.8 Hz, 23H), 3.26 (dd, *J* = 6.0, 3.3 Hz, 4H).

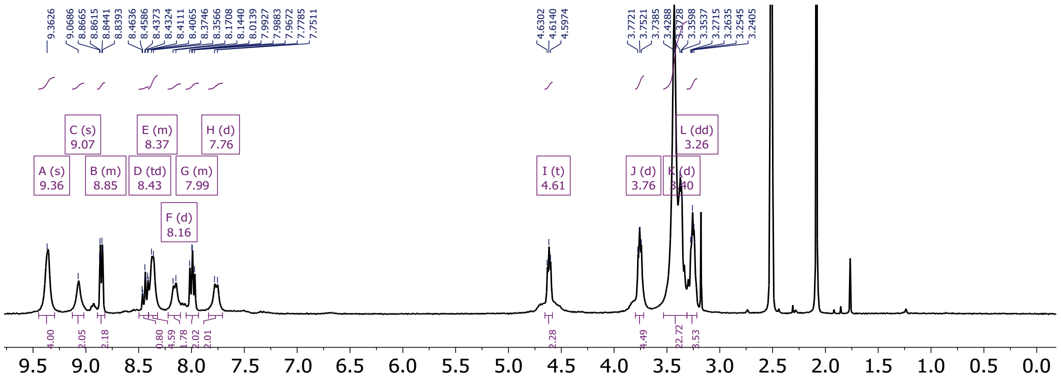

Figure S14. $[Pd_6(\mathbf{L}^{PEGPy})_{12}]$ assembly, ^{1}H NMR in dmso-d$_6$.

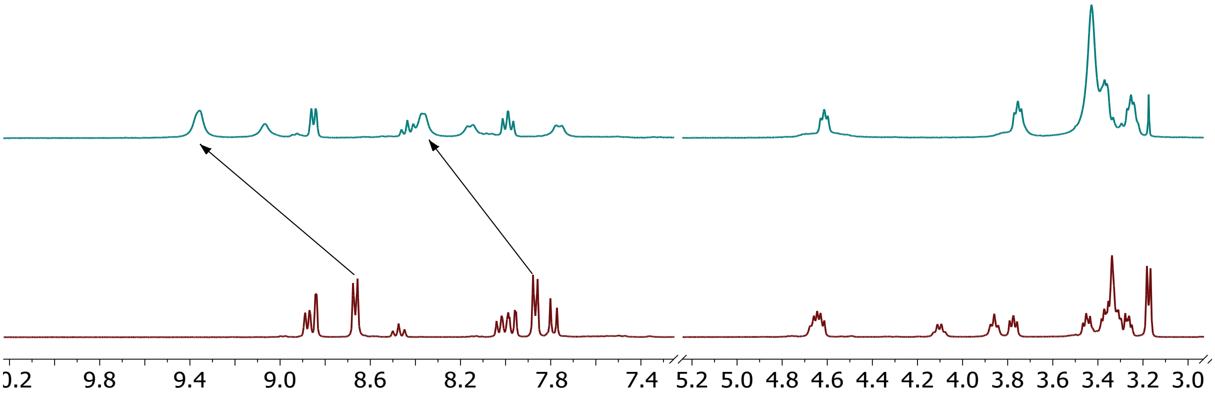

Figure S15. $[Pd_6(\mathbf{L}^{PEGPy})_{12}]$ assembly (top) and free building block (bottom), ^{1}H NMR in dmso-d$_6$.

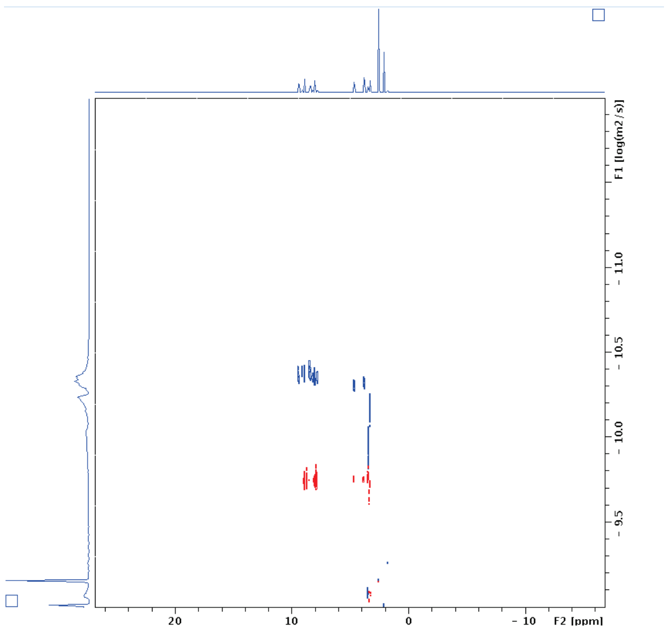

Figure S16. Overlay of [Pd_6($\mathbf{L}^{\mathbf{PEGPy}}$)$_{12}$] assembly (blue) and free building block (red), DOSY NMR of both recorded in dmso-d_6 at 300K.

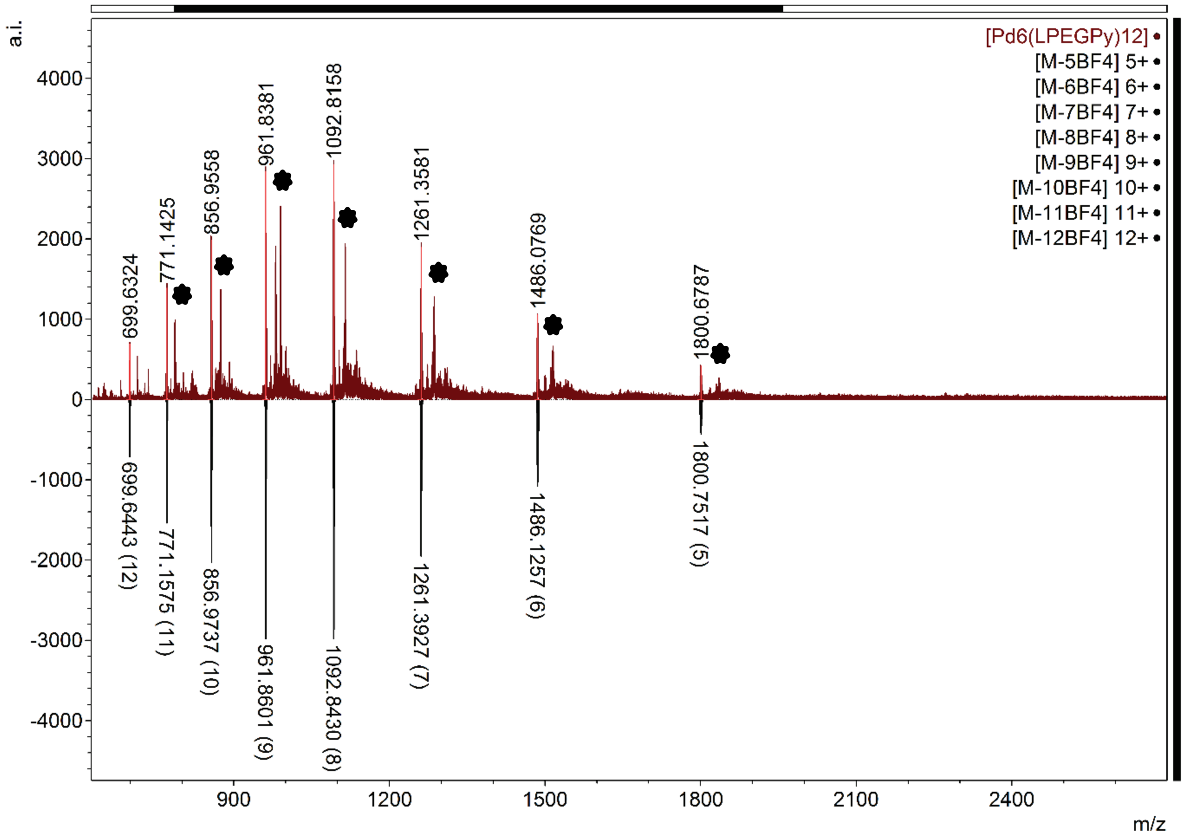

Figure S17. Full ESI-MS spectra of [Pd_6($\mathbf{L}^{\mathbf{PEGPy}}$)$_{12}$]$^{24+}$($BF_4^-$)$_{24}$. Below the simulated spectra, above obtained spectra. (Signals marked with a * represent cage signals together with solvent, e.g. 2 dmso + 1 water).

Table S18. Zoom into ESI-MS spectra of $[Pd_6(\mathbf{L^{PEGPy}})_{12}]^{x+}(BF_4^-)_{24-x}$ (below the simulated spectra, above obtained spectra).

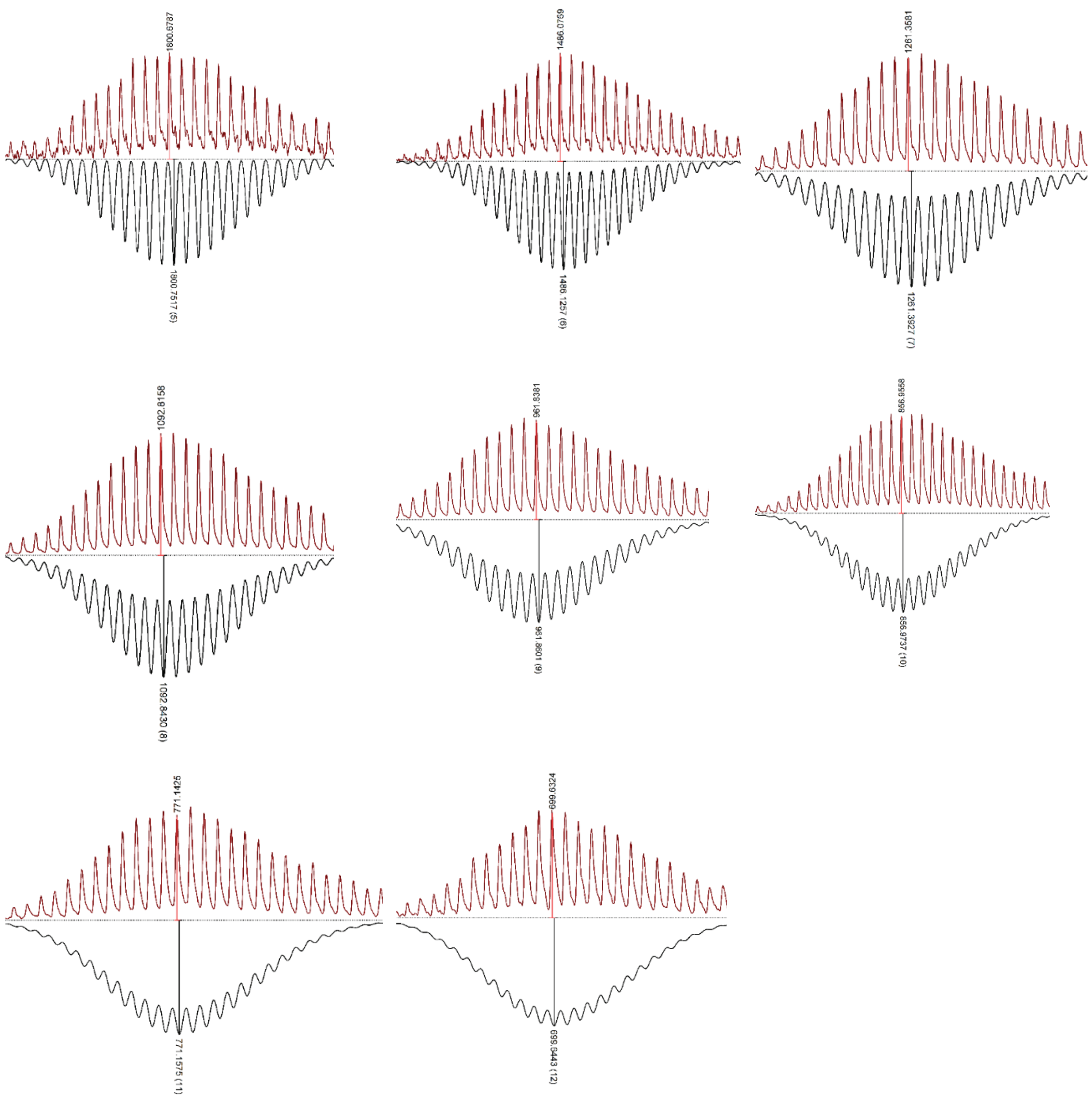

Fullerene Binding General Considerations (S3)

All herein presented assemblies were obtained as 0.83 mM solutions in dmso-d_6. Fullerene binding experiments were performed by addition of solid C_{60} (9.6 mg, 13 mmol, 16 eq.) or C_{70} (11.2 mg, 13 mmol, 16 eq.) to the corresponding solutions. The suspensions were stirred over night at room temperature, filtered (over a syringe filter) and analyzed. In a similar fashion also extraction of fullerenes from soot was performed. For the soot extraction, soot (50mg) was added to a 0.415 mM solution of the corresponding sphere and stirred overnight.

Binding studies were performed by addition of 1mM solutions of the corresponding fullerenes in toluene to sphere solutions in dmso. A black titration without any sphere was performed individually for C_{70} and C_{60}. The absorption of pure C_{70} and C_{60} was subtracted from the corresponding titration points.

All investigated spheres are multivalent fullerene binders, with four binding sites, as supported by MS studies. In order to achieve the best fit[3] for the binding multiple binding cases were briefly investigated. First, a simple case of non-cooperative binding between fullerene and individual binding sites was studied exemplary on $Pd_6\mathbf{L^N}_{12}$. To achieve this, the sphere was treated as four or three

separate hosts with no cooperativity (by simply multiplying the sphere concentration by a factor 3 or 4). This way an intrinsic binding constant K_{int} can be obtained (in the assumption of 1:1 interaction between fullerene and binding sites), that together with a statistical factor allow for the determination of the binding constants for each successive step. By implying this method, we observe a sigmoidal residuals errors and a high fitting covariances ($2.14e^{-2}$) with a binding constant for C_{70} and $Pd_6\mathbf{L}^{\mathbf{N}}_{12}$ of $2.12{\cdot}10^6$ with an error of 23% (see Fig. S19), implying that this binding model is not appropriate describing the system (similar trend is also observed for a 1:3 fit, Fig. S20).

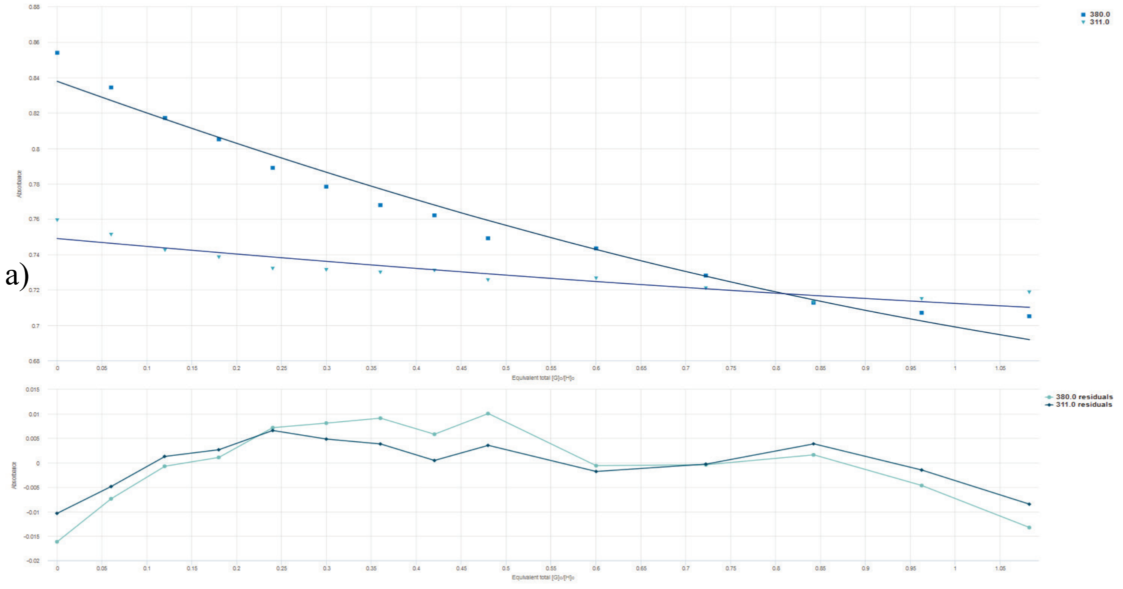

Details

**Time to fit**	0.2916 s
**SSR**	1.1808e-3
**Fitted datapoints**	28
**Fitted params**	5

Parameters

Parameter (bounds)	Optimised	Error	Initial
**K** (0 → ∞)	2118842.66 M⁻¹	± 20.2574 %	100.00 M⁻¹

Quality of fit

Fit	RMS	Covariance
**380.0**	7.8109e-3	2.8949e-2
**311.0**	4.8307e-3	0.1423
**Total**	**6.4941e-3**	**2.1449e-2**

Coefficients

Fit	H	HG
**380.0**	503567.2315	373617.0911
**311.0**	450128.7992	424911.8646

Figure S19. A) Titration data fitted to a non-cooperative model using the concentration of binding sites; b) the residual from the fit; and c) parameters extracted from the fitting. The high error and covariances together with the sigmoidal residuals indicate that a non-cooperative 4:1 model is not appropriate to describe the binding of C_{70} to $Pt_6L^N_{12}$.

a)

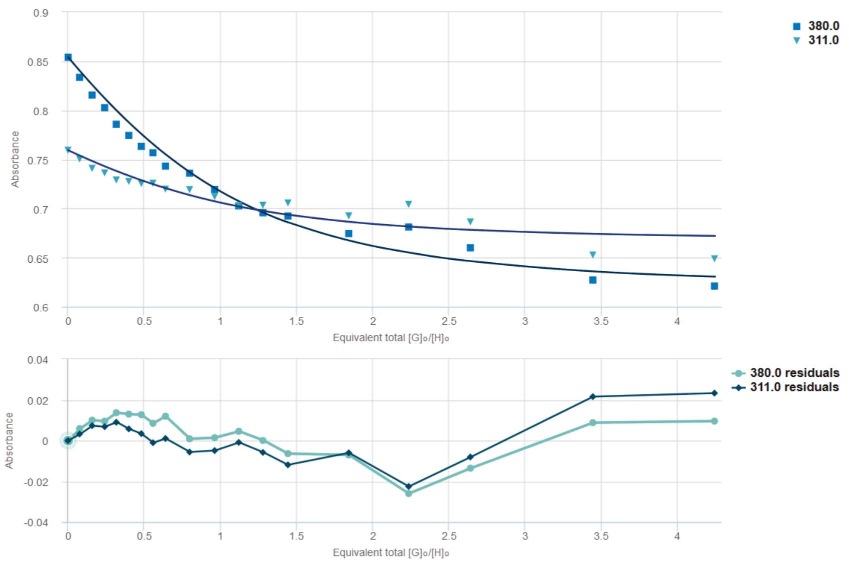

b)

Details

**Time to fit**	0.3337 s
**SSR**	4.1911e-3
**Fitted datapoints**	38
**Fitted params**	3

Parameters

Parameter (bounds)	Optimised	Error	Initial
K (0 → ∞)	1753661.14 M^{-1}	± 14.4522 %	1000.00 M^{-1}

c)

Quality of fit

Fit	RMS	Covariance
380.0	1.0519e-2	2.3526e-2
311.0	1.0485e-2	0.1389
**Total**	**1.0502e-2**	**2.7265e-2**

Coefficients

Fit	H	HG
380.0	684375.0000	468281.5960
311.0	608461.5385	524124.0539

Figure S20. A) Titration data fitted to a non-cooperative model using the concentration of 3 binding sites; b) the residual from the fit; and c) parameters extracted from the fitting. The high error and covariances together with the sigmoidal residuals indicate that a non-cooperative 3:1 model is not appropriate to describe the binding of C_{70} to $Pt_6L^N{}_{12}$.

Attempts to fit the binding in a 1:4 fashion manually (using the concentration of host as guest in a regular fashion, Figure S21) yield a slightly higher binding constant for the first event (K_{HG1} = 1.2·10^7 M^{-1}) and a comparable binding constant for the second binding (K_{HG2} = 1.2·10^6 M^{-1}). The third and fourth binding events were basically absent with binding constants of K_{HG3} = 5 M^{-1} and K_{HG4} = 0.5 M^{-1}. Because the third and fourth binding constants were in comparison to the first two very low, little influence of the third and fourth binding event under the diluted concentrations of the titration experiments were expected. As the binding constant determination is performed in mixtures of toluene and dmso, we anticipate lower binding than in pure dmso due to competition (solvation of fullerene by toluene). In line with that, when we determined the distribution of fullerene bound to a nanosphere $Pd_6\mathbf{L^N}{}_{12}$ in absence and presence of toluene, lower bound fullerene C_{70} was obtained as depicted below (S21).

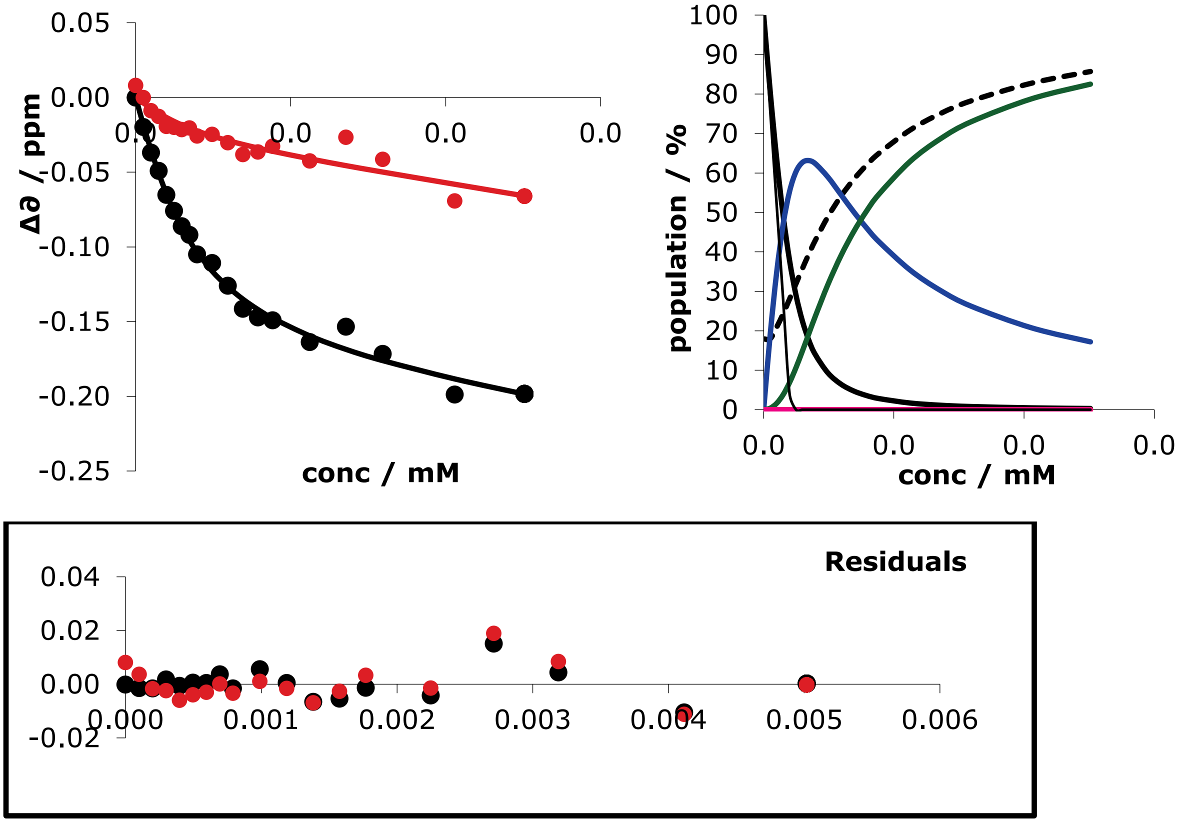

Figure S21. A) Titration data fitted to a 1:4 multiple sites model; b) the speciation graph (blue: HG, green : HG2, pink :HG3); and c) residuals. The following binding constants were obtained: (K_{HG1} = 1.2·10^{7} M^{-1}), (K_{HG2} = 1.2·10^{6} M^{-1}), (K_{HG3} = 5 M^{-1}) and (K_{HG4} = 0.5 M^{-1}) with 82% HG2, 18% HG and 0% HG3 or HG4 at the end concentration.

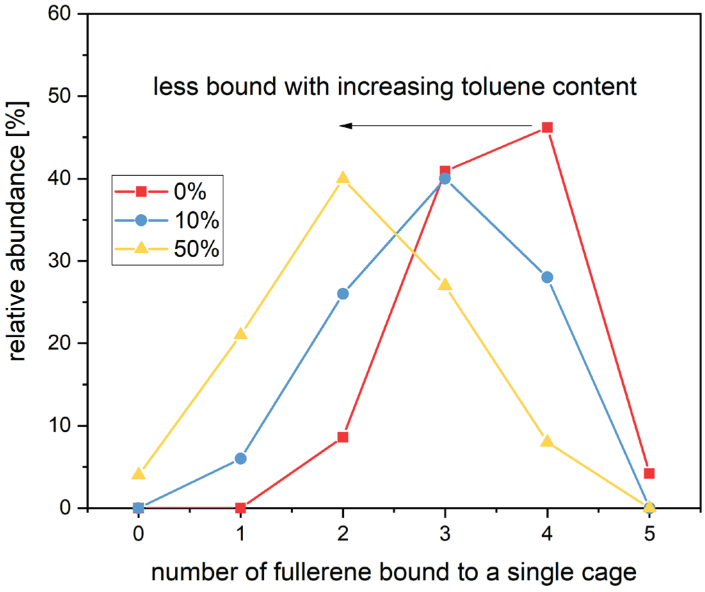

Figure S22. MS distribution analysis of a sample of C_{70} and $Pt_6L^{N}_{12}$ in dmso with different amount of toluene.

When a non-cooperative 1:2 model using the sphere concentration was applied (under the assumption that at the low concentration four fullerenes are not able to bind to the self-assembly), the binding constant was determined to be in a similar range ($2.61{\cdot}10^6$) (see Fig. S21). The 1:2 model non-cooperative model shows lower error and fitting covariances ($3.44e^{-3}$) together with a lower overall error (6%). Thus, this model is a better description of our system.

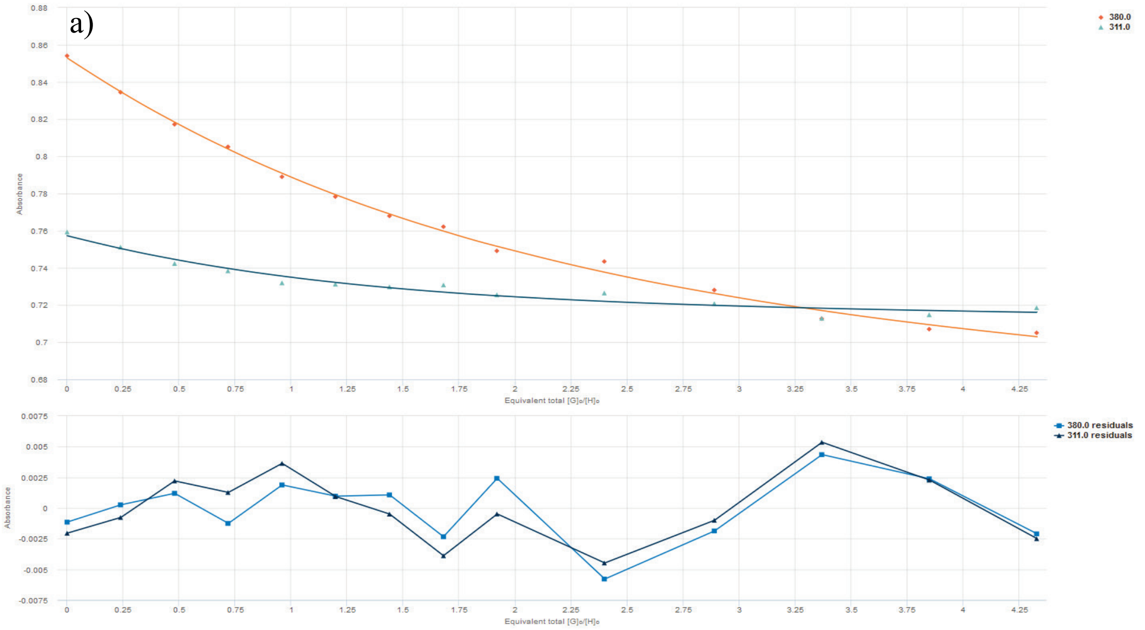

Details

**Time to fit**	0.5516 s
**SSR**	1.8960e-4
**Fitted datapoints**	28
**Fitted params**	7

Parameters

Parameter (bounds)	Optimised	Error	Initial
K_{11} (0 → ∞)	2610950.00 M^{-1}	± 6.1416 %	1000.00 M^{-1}

Quality of fit

Fit	RMS	Covariance
380.0	2.4979e-3	2.9605e-3
311.0	2.7024e-3	4.4543e-2
Total	**2.6022e-3**	**3.4438e-3**

Coefficients

Fit	H	HG	HG2
380.0	2050356.7300	1696996.6685	1628024.1161
311.0	1820446.8760	1691487.9065	1802570.3193

Figure S23. A) Titration data fitted to a non-cooperative model using the concentration of sphere; b) the residual from the fit; and c) parameters extracted from the fitting. The low error and covariances together indicate that a non-cooperative 2:1 model is appropriate to describe the binding of C_{70} to $Pt_6L^N{}_{12}$.

On an important note: The 1:2 non-cooperative binding which is applied for the determination of the binding constants is based on the hypothesis that the existing third and fourth binding events are not observed or significant weak at the low concentrations. Although, their contribution may be minimal at the low concentrations, the obtained binding constants can be prone to errors and should be only be referenced to similar systems or seen as a rough estimation. As all herein presented systems have very similar 4 binding sites and we apply the same model for the fitting of all systems, each binding constant should have a similar error. This allows the comparison between the herein presented binding to all three nanospheres.

Table S24. Concentrations of host and guest for titration of fullerenes to a sphere solutions.

Host concentration / M	Guest concentration / M	Guest equivalents
4,16E-07	0	0
4,1558E-07	9,974E-08	0,24
4,1517E-07	1,9928E-07	0,48
4,1475E-07	2,9862E-07	0,72
4,1434E-07	3,9776E-07	0,96
4,1392E-07	4,967E-07	1,2
4,135E-07	5,9545E-07	1,44
4,1309E-07	6,9399E-07	1,68
4,1267E-07	7,9233E-07	1,92
4,1184E-07	9,8842E-07	2,4
4,1101E-07	1,1878E-06	2,89
4,1018E-07	1,3823E-06	3,37
4,0934E-07	1,576E-06	3,85
4,0851E-07	1,7689E-06	4,33
4,0643E-07	2,2476E-06	5,53
4,0435E-07	2,7132E-06	6,71
4,0227E-07	3,19E-06	7,93
3,9811E-07	4,1165E-06	10,34
3,9395E-07	5,0189E-06	12,74
3,8979E-07	5,9015E-06	15,14

Fullerene Binding $Pd_6L^O_{12}$ (S4)

Following the general conditions, following spectroscopic properties were obtained:

NMR studies

$C_{60}\subset Pd_6\mathbf{L^O}_{12}$: ^{1}H NMR (300 MHz, DMSO-d_6) δ 9.34 (d, J = 5.9 Hz, 4H), 9.04 (s, 2H), 8.35 (d, J = 5.9 Hz, 4H), 8.23 (d, J = 8.9 Hz, 2H), 7.92 (d, J = 8.5 Hz, 2H). ^{13}C NMR (126 MHz, DMSO) δ 158.14, 151.79, 150.05, 142.12, 129.77, 127.85, 124.88, 123.97, 121.02, 118.55, 113.59.

$C_{70}\subset Pd_6\mathbf{L^N}_{12}$: ^{1}H NMR (300 MHz, DMSO-d_6) δ 9.38 (d, J = 6.0 Hz, 4H), 9.04 (s, 2H), 8.35 (d, J = 6.1 Hz, 4H), 8.22 (d, J = 8.6 Hz, 2H), 7.91 (d, J = 8.6 Hz, 2H). ^{13}C NMR (75 MHz, DMSO) δ 158.18, 151.73, 149.98, 149.62, 146.89, 144.10, 129.66, 127.87, 124.99, 123.91, 121.23, 118.54, 113.60.

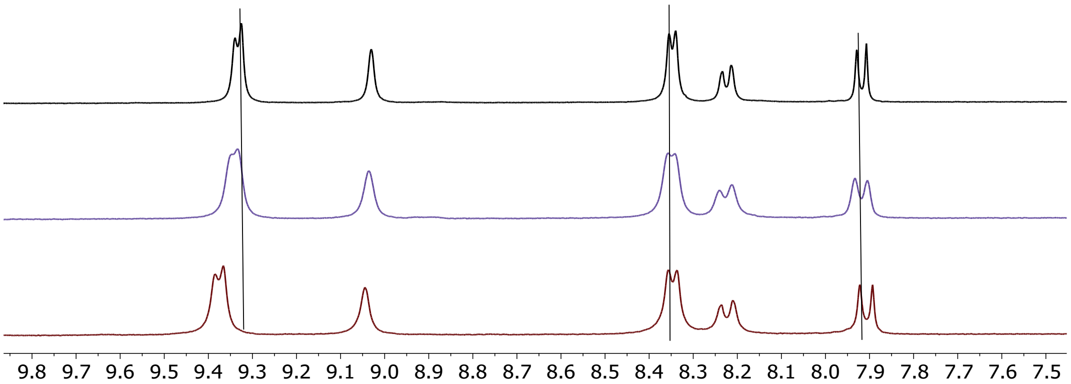

Figure S25. $[Pd_6(\mathbf{L^O})_{12}]$ assembly (top) and changes upon addition of solid C_{60} (middle) and C_{70} (bottom), ^{1}H NMR in dmso-d_6. Slight shifts of pyridine protons are visible for C_{70}.

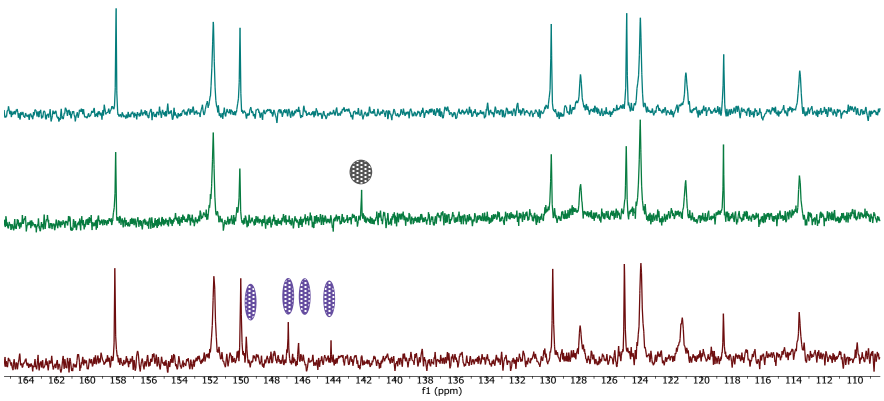

Figure S26. [Pd_6(**L**^{O})$_{12}$] assembly (top) and changes upon addition of solid C_{60} (middle) and C_{70} (bottom), ^{13}C NMR in dmso-d$_6$.

<u>MS studies</u>

Table S27. Analysis of C_{60}⊂Pd_6**L**$^O_{12}$ MS spectrum. Displayed are the counts for different amount of fullerene bound to the assembly and the resulting relative abundancy of the corresponding species.

Equiv. Fullerene	Counts	Relative abundance [%]
0	201900	87
1	29863	13
2	0	0
3	0	0
4	0	0
5	0	0

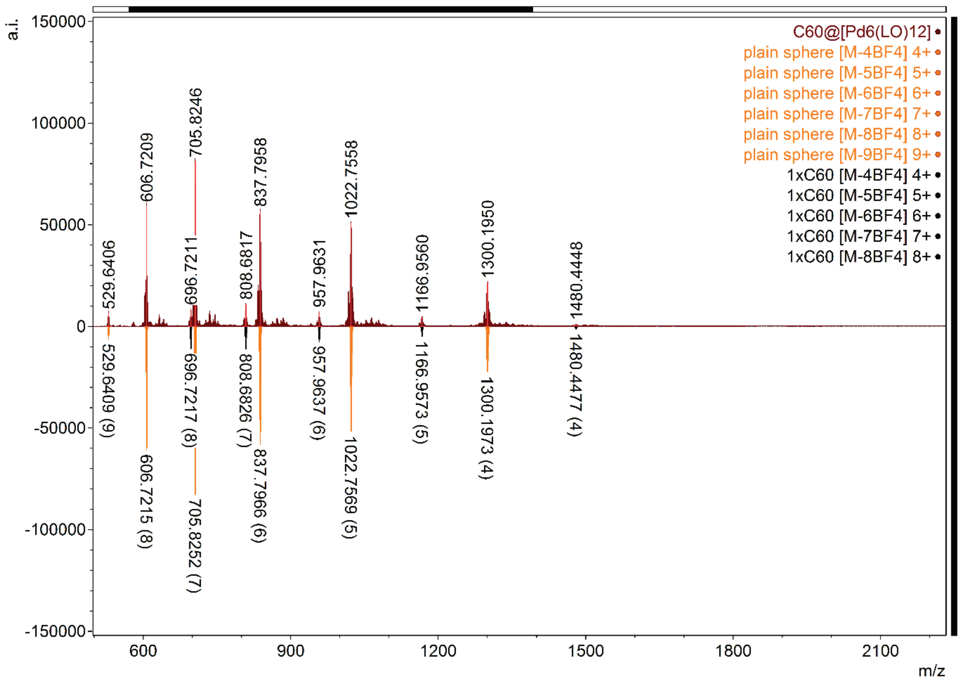

Figure S28. Full ESI-MS spectra of C_{60}⊂Pd_6**L**$^O_{12}$. Below the simulated spectra, above obtained spectra.

Table S29. Zoom into ESI-MS spectra of $1C_{60}\subset Pd_6$**L**$^{O}_{12}$ (below the simulated spectra, above obtained spectra).

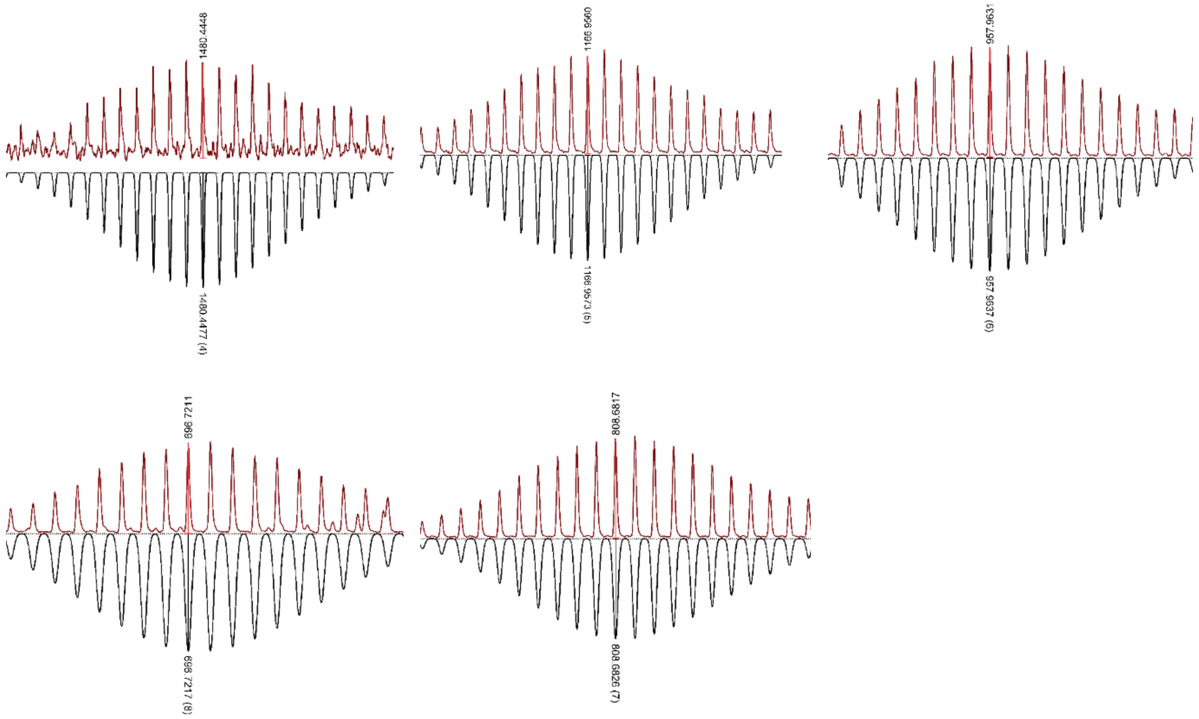

$C_{70}\subset Pd_6$**L**$^{O}_{12}$

Table S30. Analysis of $C_{70}\subset Pd_6$**L**$^{O}_{12}$ MS spectrum. Displayed are the counts for different amount of fullerene bound to the assembly and the resulting relative abundancy of the corresponding species.

Equiv. Fullerene	Counts	Relative abundance [%]
0	99200	32
1	141200	46
2	57800	19
3	9095	3
4	0	0
5	0	0

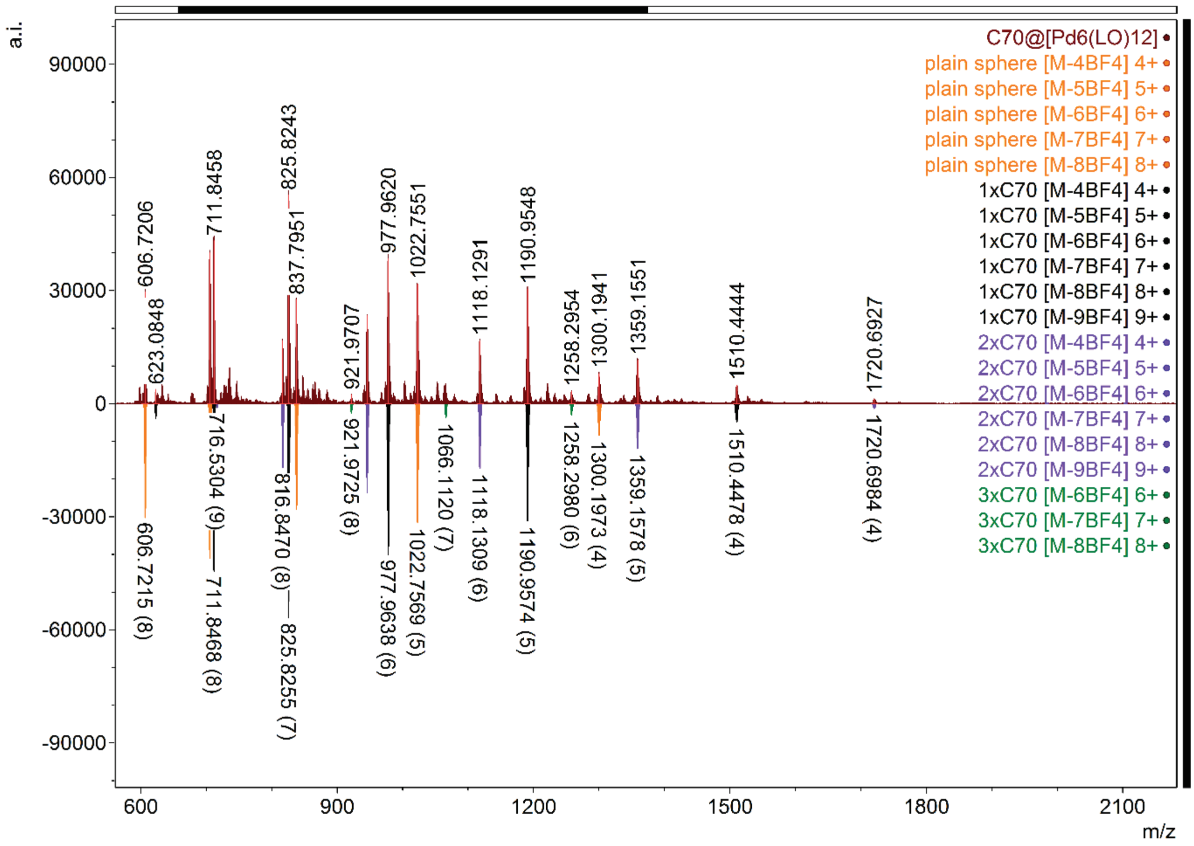

Figure S31. Full ESI-MS spectra of $C_{70}{\subset}Pd_6\mathbf{L}^{O}_{12}$. Below the simulated spectra, above obtained spectra.

Table S32. Zoom into ESI-MS spectra of $xC_{70}{\subset}Pd_6\mathbf{L}^{O}_{12}$ for x=1-3 (below the simulated spectra, above obtained spectra).

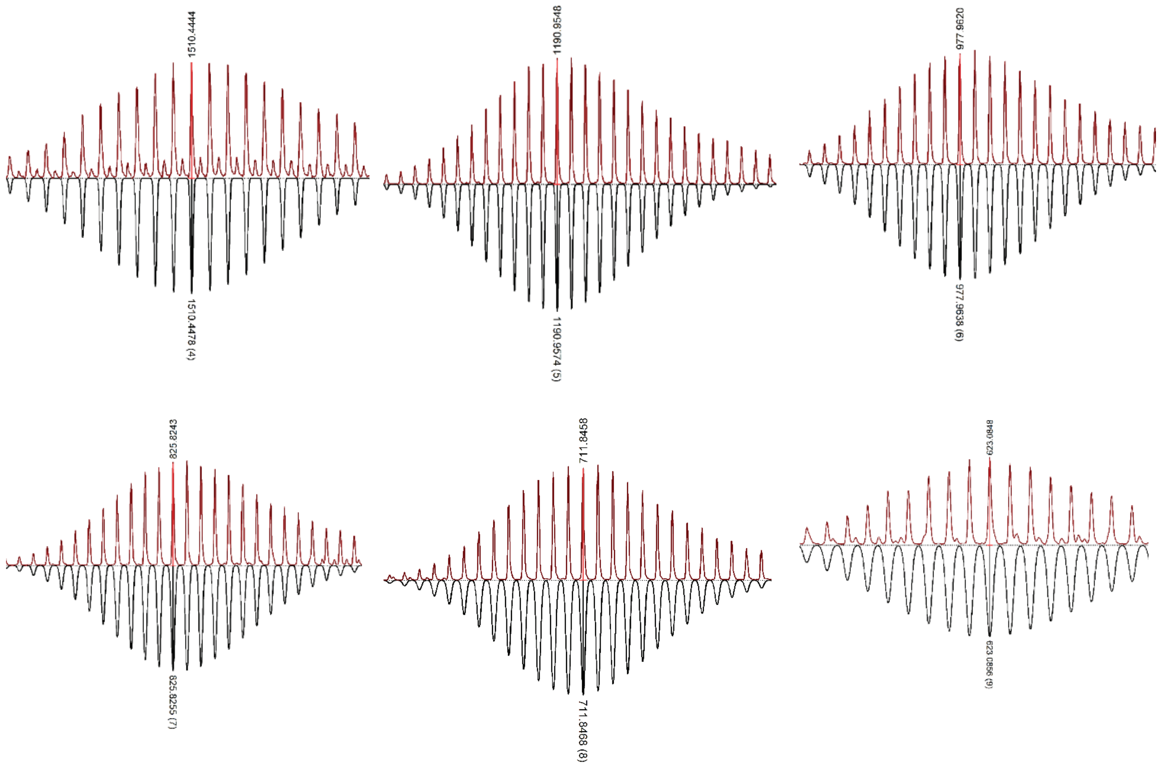

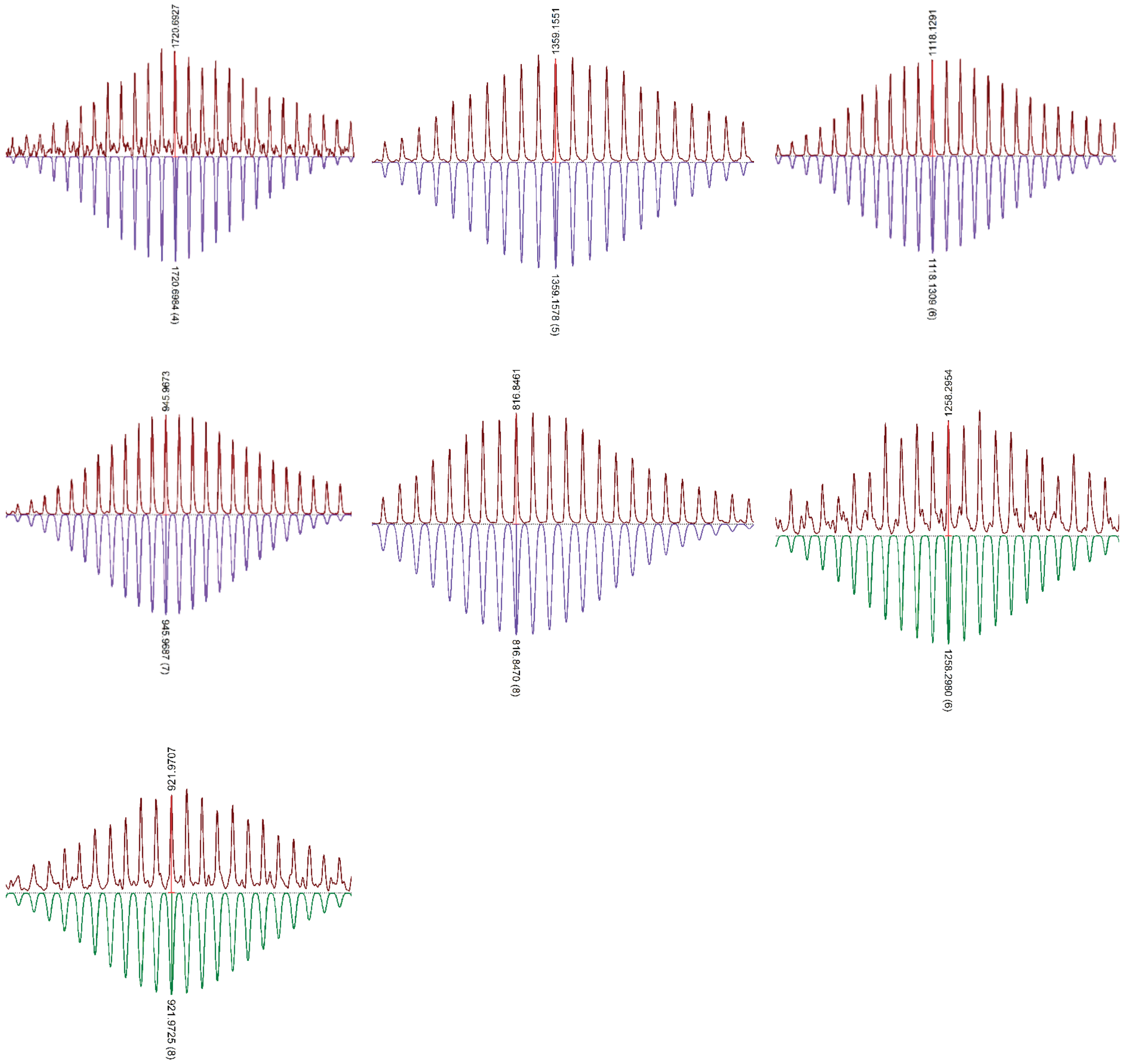

UV/VIS

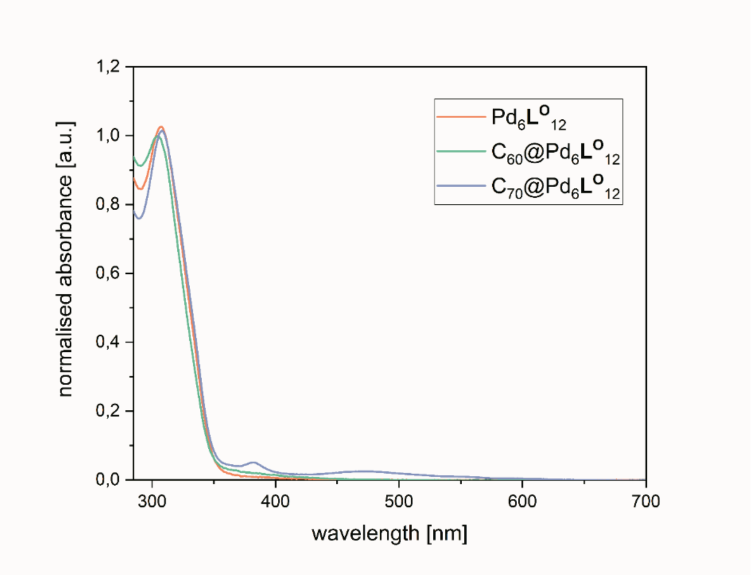

Figure S33. Normalized UV/VIS spectra of Pd_6**L**$^{O}_{12}$, C_{60}⊂Pd_6**L**$^{O}_{12}$ and C_{70}⊂Pd_6**L**$^{O}_{12}$.

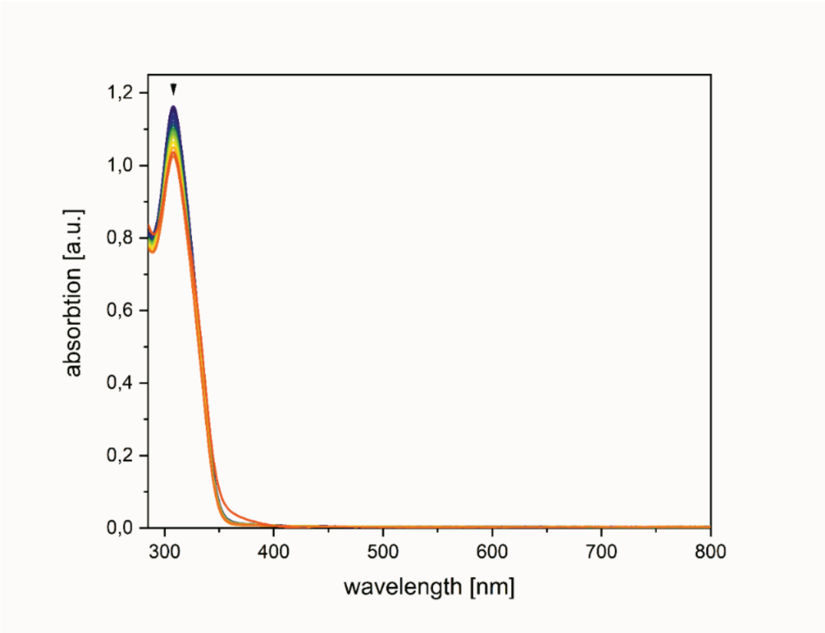

Figure S34. UV/VIS spectra of $Pd_6\mathbf{L}^{\mathbf{O}}_{12}$ upon addition of C_{60}.

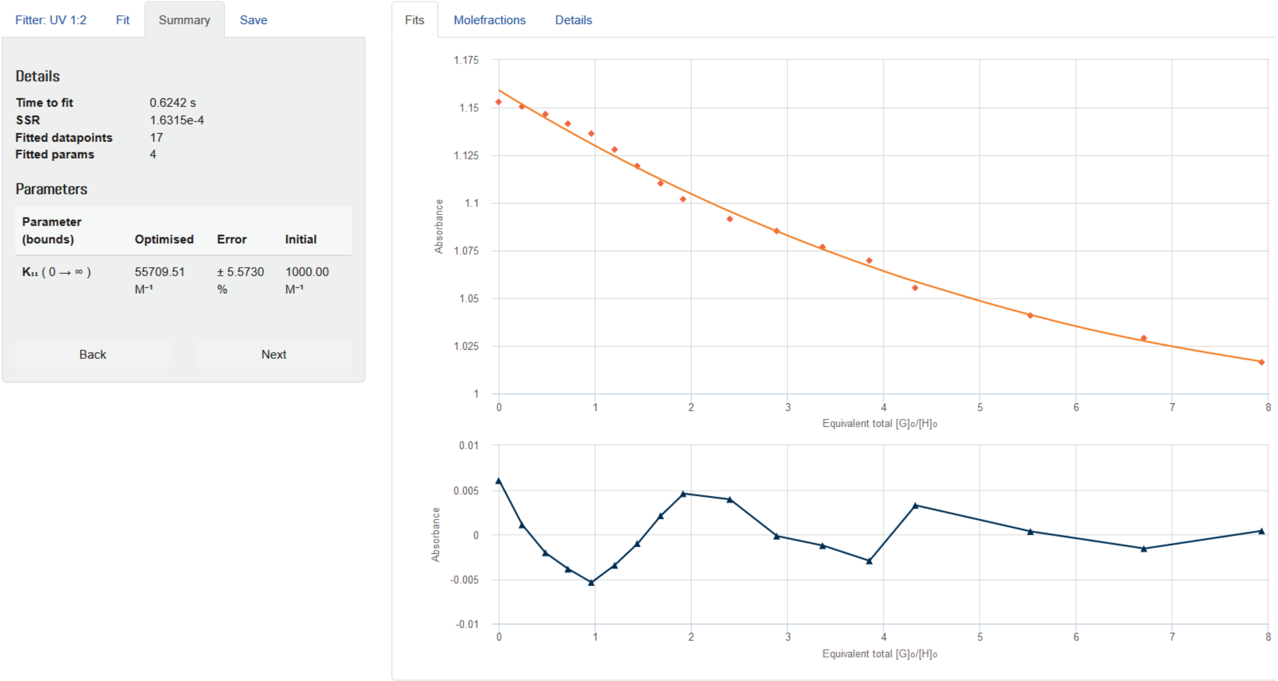

Figure S35. Non-cooperative 1:2 H:G fitting to obtain binding constant between C_{60} and $Pd_6\mathbf{L}^{\mathbf{O}}_{12}$ (330 nm).

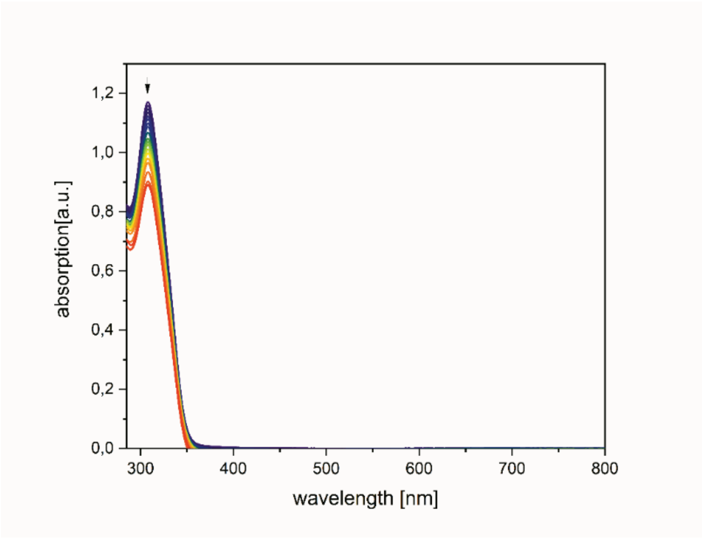

Figure S36. UV/VIS spectra of $Pd_6\mathbf{L^O}_{12}$ upon addition of C_{70}.

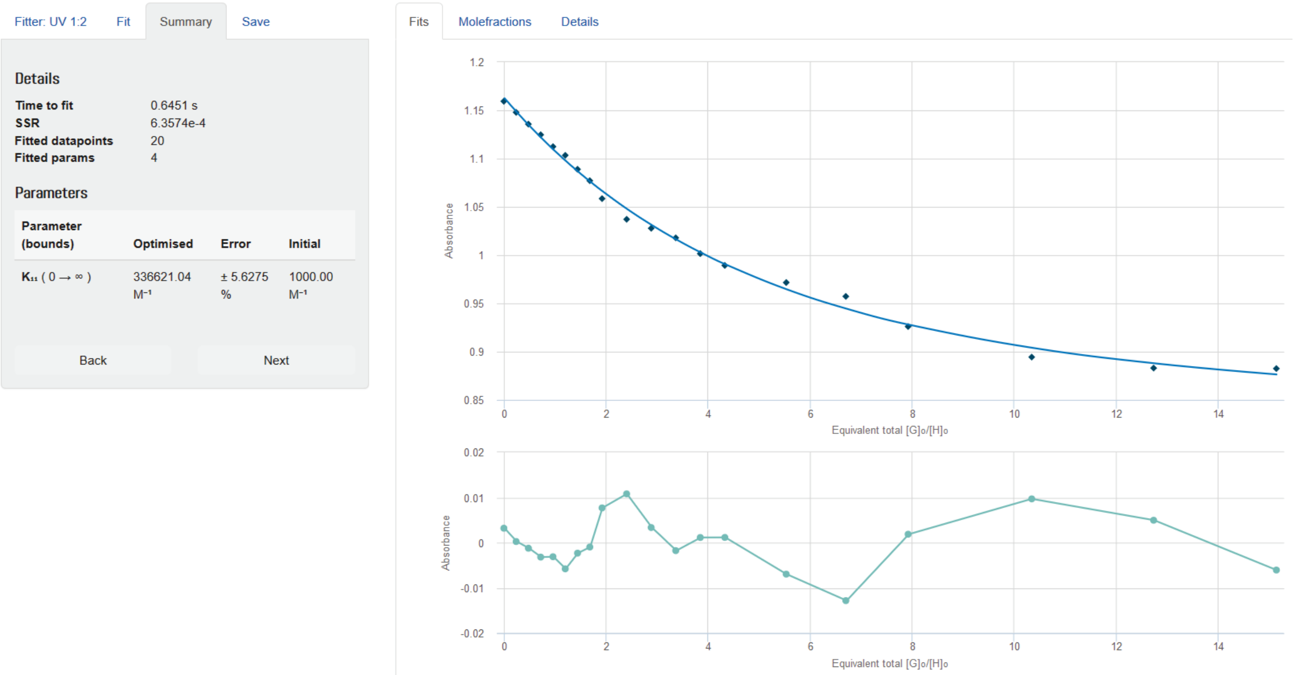

Figure S37. Fitting to obtain binding constant between C_{70} and $Pd_6\mathbf{L^O}_{12}$ (330 nm).

CV

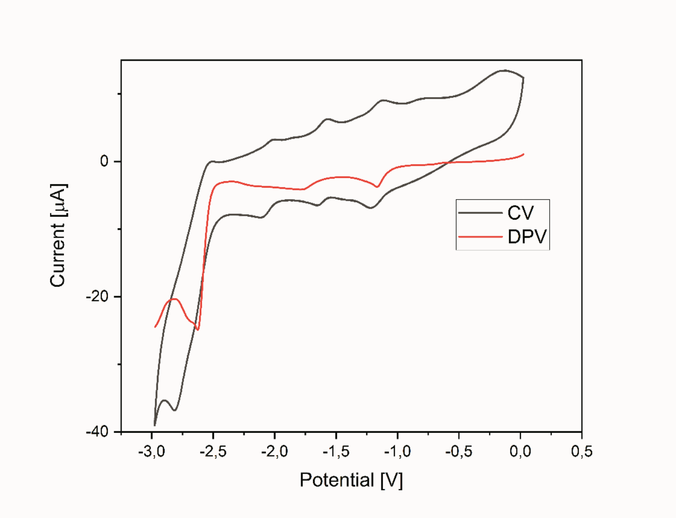

Figure S38. Reductive behavior of C_{70}⊂Pd_6**L**$^{O}_{12}$ in dmso by cyclic voltammetry (black) and by differential pulse voltammetry (red). V versus Fc/Fc$^+$.

Fullerene Binding $Pd_6L^N_{12}$ (S5)

Soot extraction was performed by addition of 50 mg fullerene soot to 1mL of sphere solution in dmso. The solids were removed after stirring the solution over night by multiple runs over syringe filers. Following the general conditions, following spectroscopic properties were obtained:

NMR studies

C_{60}⊂Pd_6**L**$^{N}_{12}$: ^{1}H NMR (500 MHz, Acetonitrile-d_3) δ 9.37 (s, 4H), 9.15 (s, 2H), 8.34 (d, *J* = 5.9 Hz, 4H), 8.14 (d, *J* = 8.0 Hz, 2H), 7.83 – 7.72 (m, 2H), 7.18 (d, *J* = 8.0 Hz, 2H), 7.02 (d, *J* = 8.0 Hz, 2H), 5.66 (s, 2H), 1.10 (s, 9H). ^{13}C NMR (126 MHz, CD_3CN) δ 150.76, 150.16, 149.69, 142.45, 141.45, 133.94, 126.24, 125.25, 123.19, 122.60, 120.10, 111.08, 54.82, 40.03, 39.94, 39.87, 39.78, 39.70, 39.61, 39.53, 39.44, 39.37, 39.27, 39.11, 38.94, 30.84, 1.06.

C_{70}⊂Pd_6**L**$^{N}_{12}$: ^{1}H NMR (300 MHz, DMSO-d_6) δ 9.39 (s, 4H), 9.15 (s, 2H), 8.34 (s, 4H), 8.14 (d, *J* = 8.4 Hz, 2H), 7.74 (d, *J* = 8.3 Hz, 2H), 7.18 (d, *J* = 8.1 Hz, 2H), 7.02 (d, *J* = 8.1 Hz, 2H), 5.65 (s, 2H), 1.11 (d, *J* = 2.6 Hz, 9H). ^{13}C NMR (75 MHz, DMSO) δ 151.17, 150.52, 150.13, 149.57, 146.86, 146.22, 144.12, 143.11, 134.50, 129.62, 126.80, 125.87, 125.12, 123.92, 123.12, 55.38, 40.82, 40.54, 40.26, 39.99, 39.71, 39.43, 39.15, 34.55, 31.41, 22.98, 1.62.

soot⊂Pd_6**L**$^{N}_{12}$: ^{1}H NMR (300 MHz, DMSO-d_6) δ 9.52 – 9.25 (m, 4H), 9.16 (s, 3H), 8.34 (s, 4H), 8.14 (s, 3H), 7.80 (s, 1H), 7.18 (s, 3H), 7.03 (s, 3H), 1.10 (s, 11H).

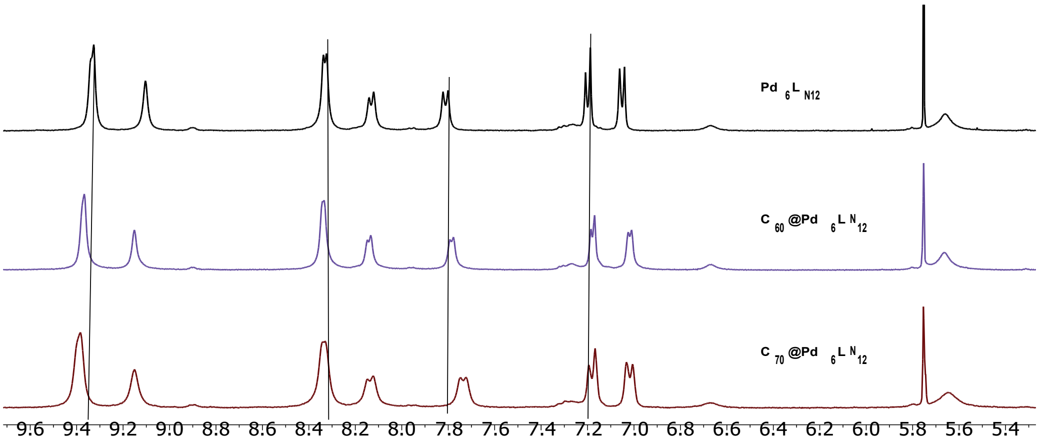

Figure S39. [Pd_6(**L**^{N})$_{12}$] assembly (top) and changes upon addition of solid C_{60} (middle) and C_{70} (bottom), ^{1}H NMR in dmso-d_6. Slight shifts of carbazole and pyridine protons are visible.

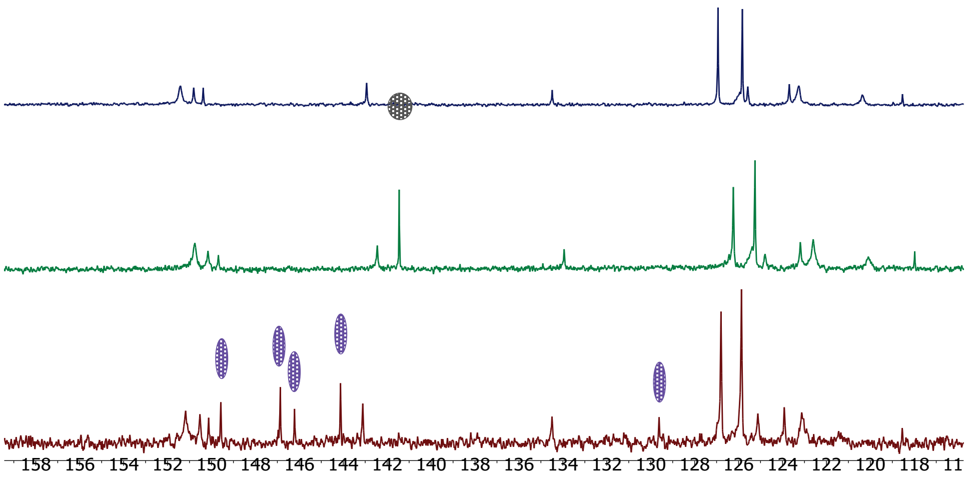

Figure S40. $[Pd_6(\mathbf{L^N})_{12}]$ assembly (top) and changes upon addition of solid C_{60} (middle) and C_{70} (bottom), ^{13}C NMR in dmso-d_6.

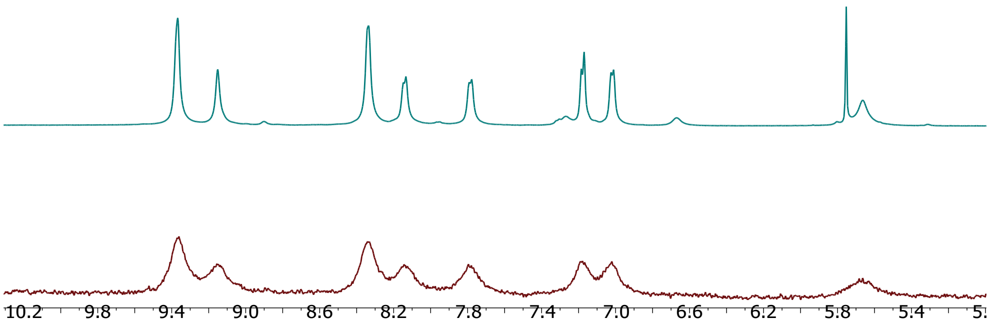

Figure S41. $[Pd_6(\mathbf{L^N})_{12}]$ assembly (top) and changes upon addition of soot (bottom), ^{1}H NMR in dmso-d_6. Broadening of all signals (including solvent) is visible.

<u>MS studies</u>

$C_{60}\subset Pd_6\mathbf{L^N}_{12}$

Table S42. Analysis of $C_{60}\subset Pd_6\mathbf{L^N}_{12}$ MS spectrum. Displayed are the counts for different amount of fullerene bound to the assembly and the resulting relative abundancy of the corresponding species.

Equiv. Fullerene	Counts	Relative abundance [%]
0	145100	29
1	227000	45
2	110400	22
3	20289	4
4	0	0
5	0	0

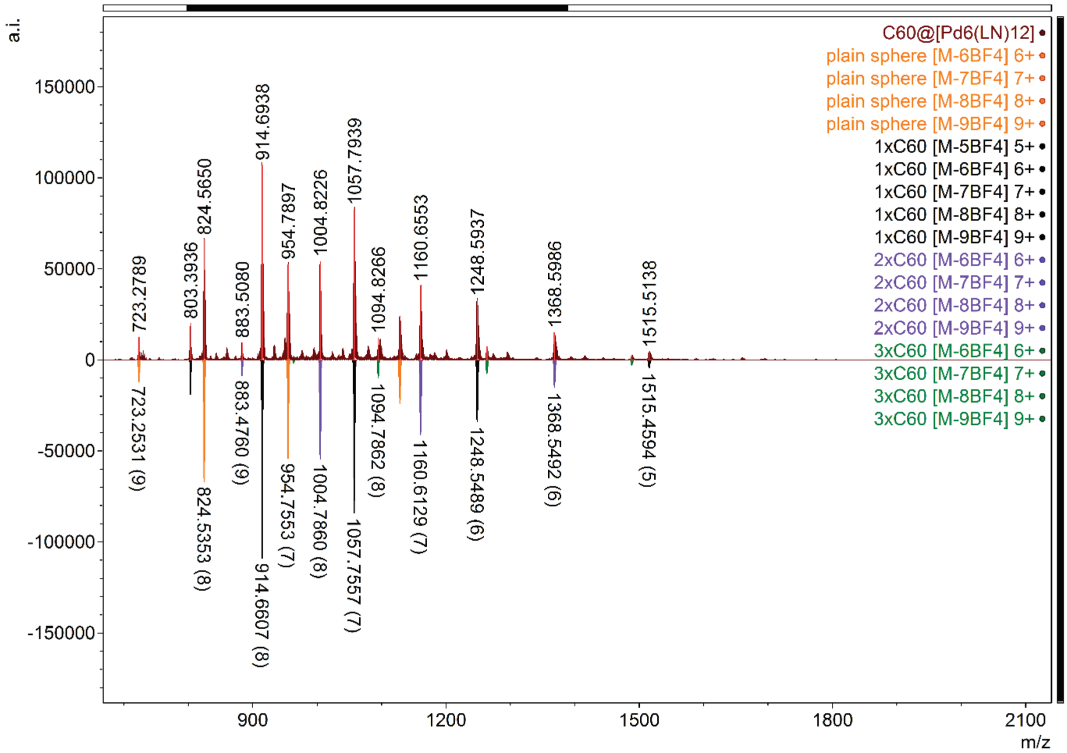

Figure S43. Full ESI-MS spectra of $C_{60}{\subset}Pd_6\mathbf{L}^{N}_{12}$. Below the simulated spectra, above obtained spectra.

Table S44. Zoom into ESI-MS spectra of $xC_{60}{\subset}Pd_6\mathbf{L}^{N}_{12}$ for x=1-3 (below the simulated spectra, above obtained spectra).

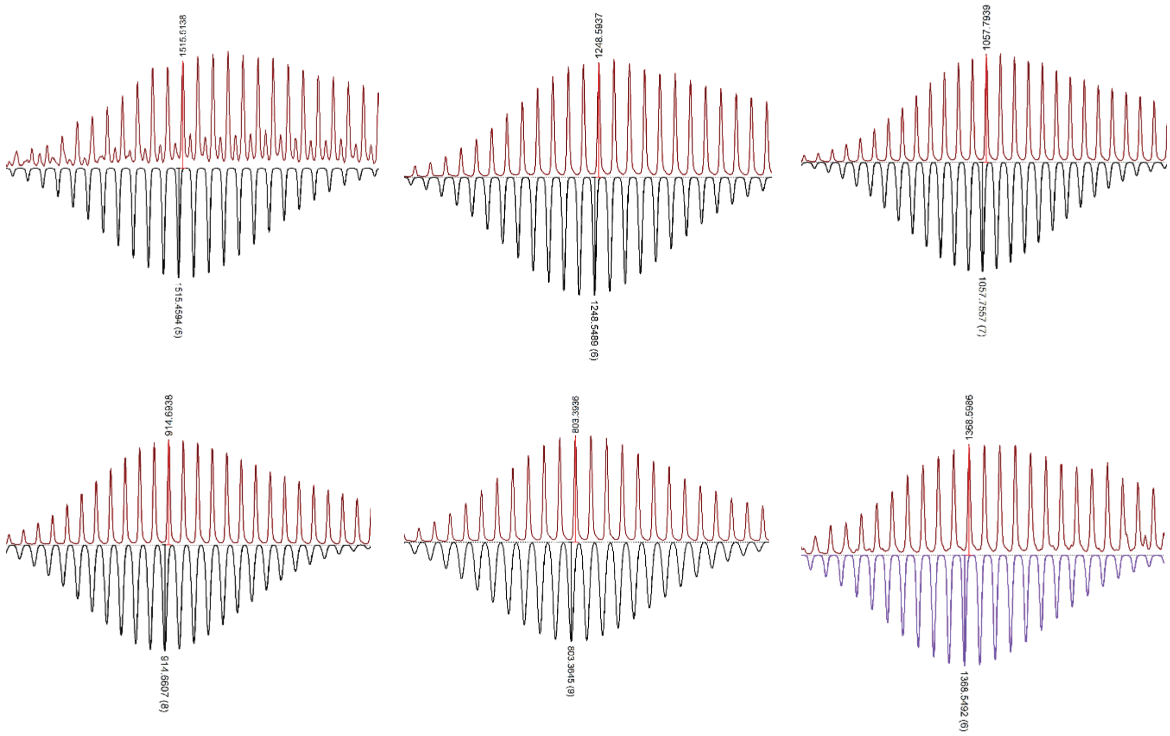

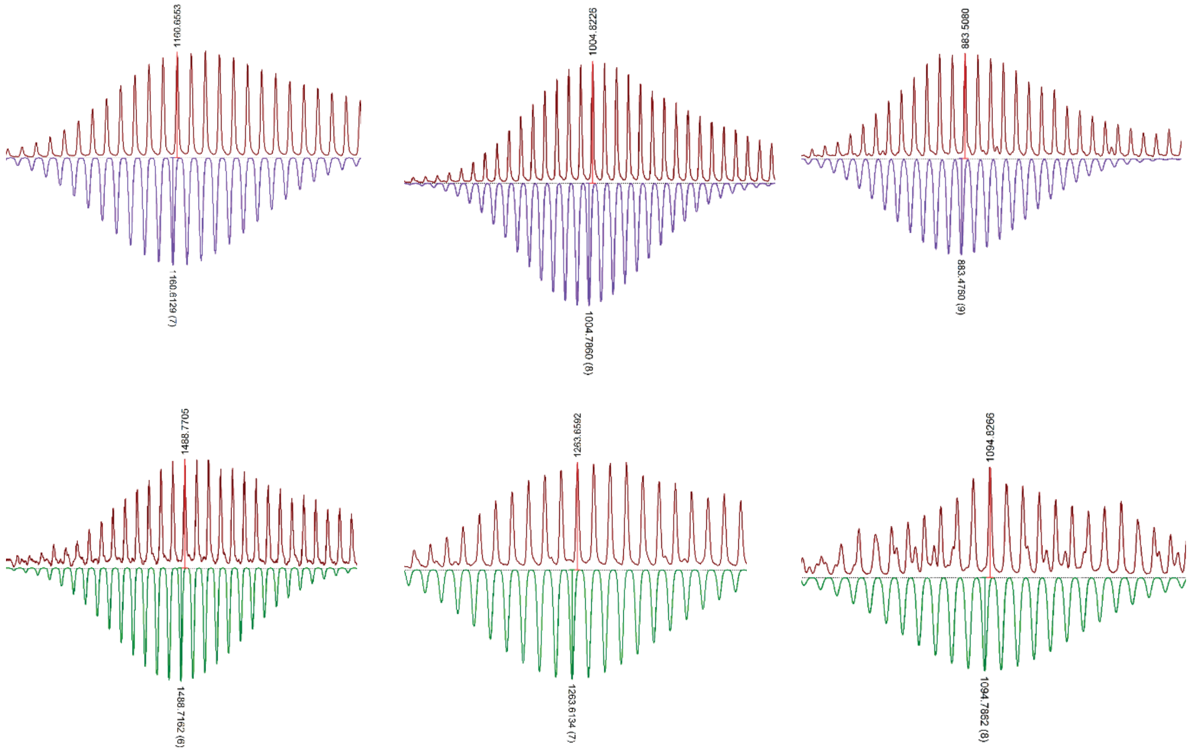

C_{70}⊂Pd_6**L**$^{\mathbf{N}}_{12}$

Table S45. Analysis of C_{70}⊂Pd_6**L**$^{\mathbf{N}}_{12}$ MS spectrum. Displayed are the counts for different amount of fullerene bound to the assembly and the resulting relative abundancy of the corresponding species.

Equiv. Fullerene	Counts	Relative abundance [%]
0	0	0
1	0	0
2	8590	9
3	40825	41
4	46096	46
5	4254	4

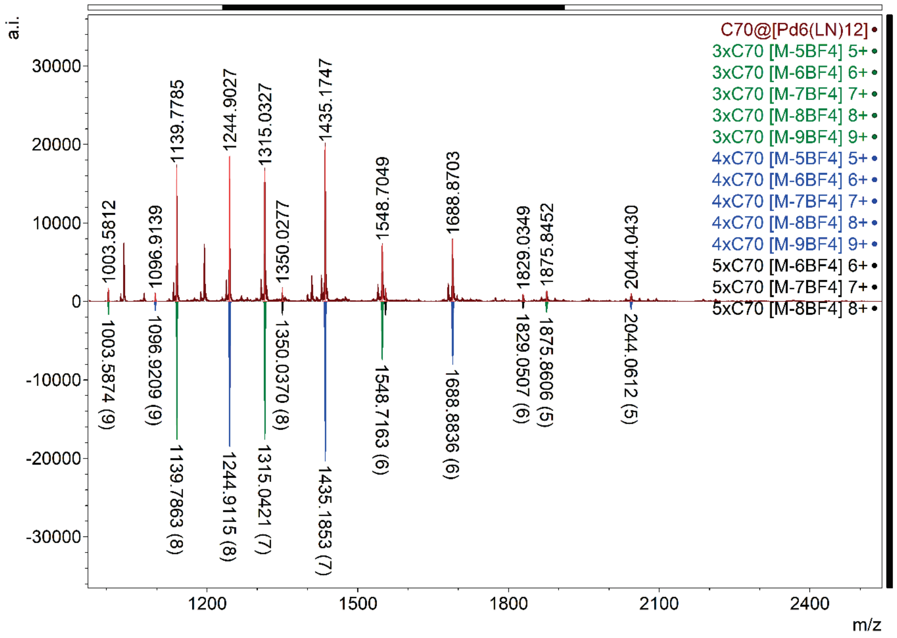

Figure S46. Full ESI-MS spectra of $C_{70}{\subset}Pd_6\mathbf{L^N}_{12}$. Below the simulated spectra, above obtained spectra.

Table S47. Zoom into ESI-MS spectra of $xC_{70}{\subset}Pd_6\mathbf{L^N}_{12}$ for x=3-5 (below the simulated spectra, above obtained spectra).

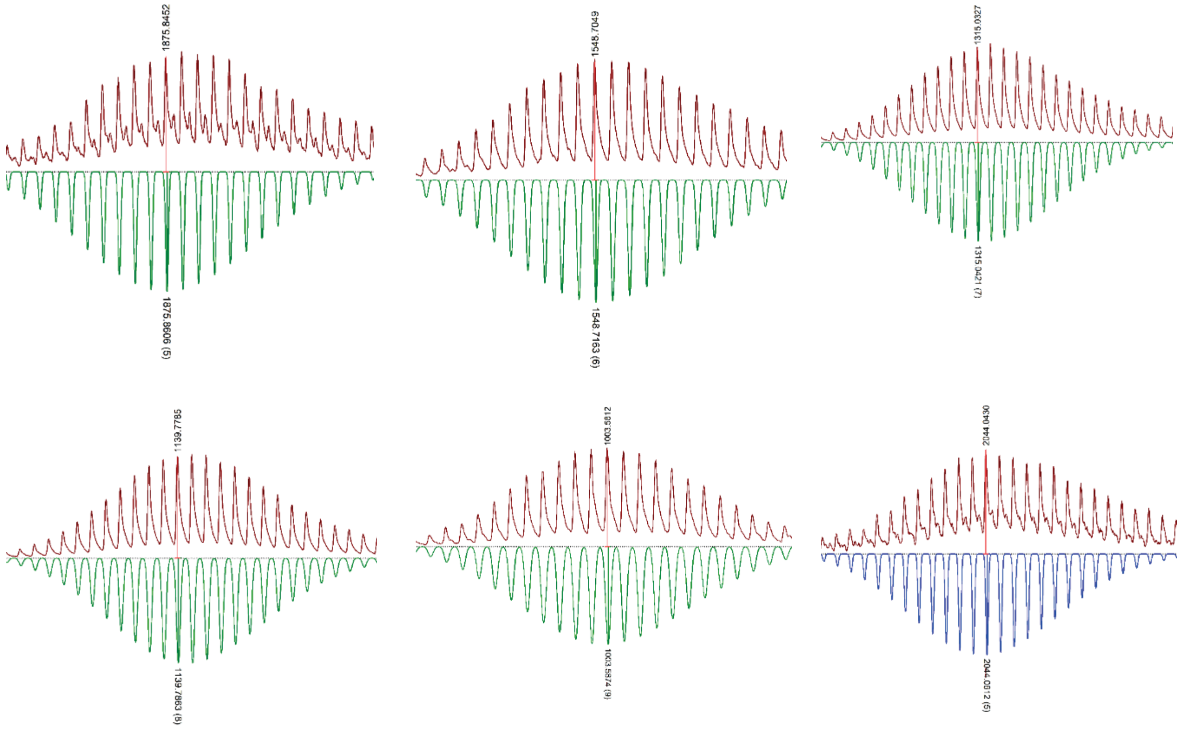

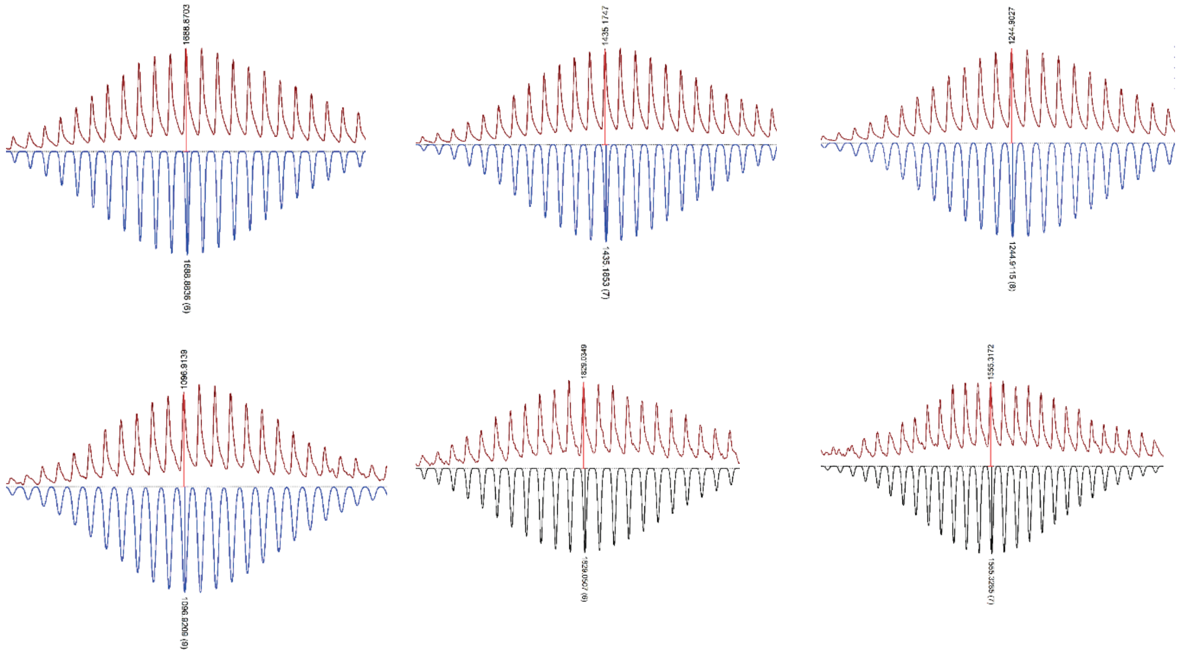

soot⊂Pd$_6$**L**$^{\mathbf{N}}_{12}$

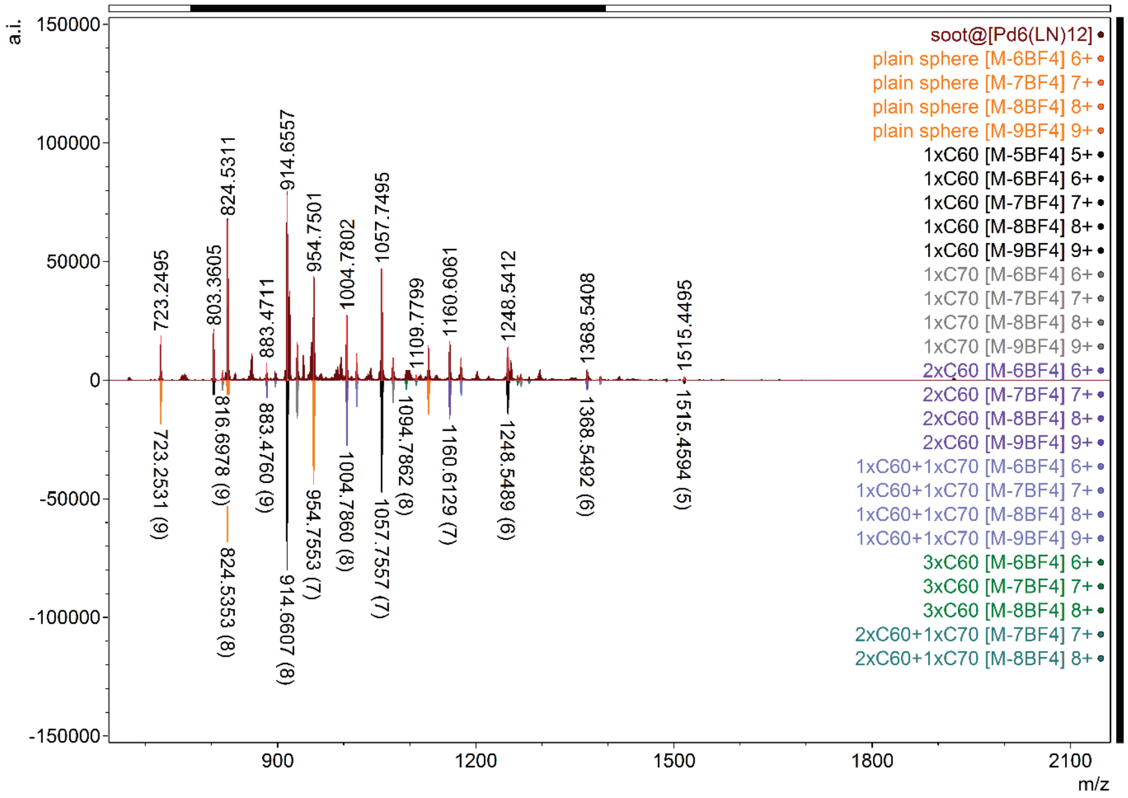

Figure S48. Full ESI-MS spectra of xC$_{70}$+yC$_{60}$⊂Pd$_6$**L**$^{\mathbf{N}}_{12}$. Below the simulated spectra, above obtained spectra.

Table S49. Zoom into ESI-MS spectra of xC_{70}+yC_{60}⊂$Pd_6$$\mathbf{L}^{\mathbf{N}}_{12}$ (below the simulated spectra, above obtained spectra).

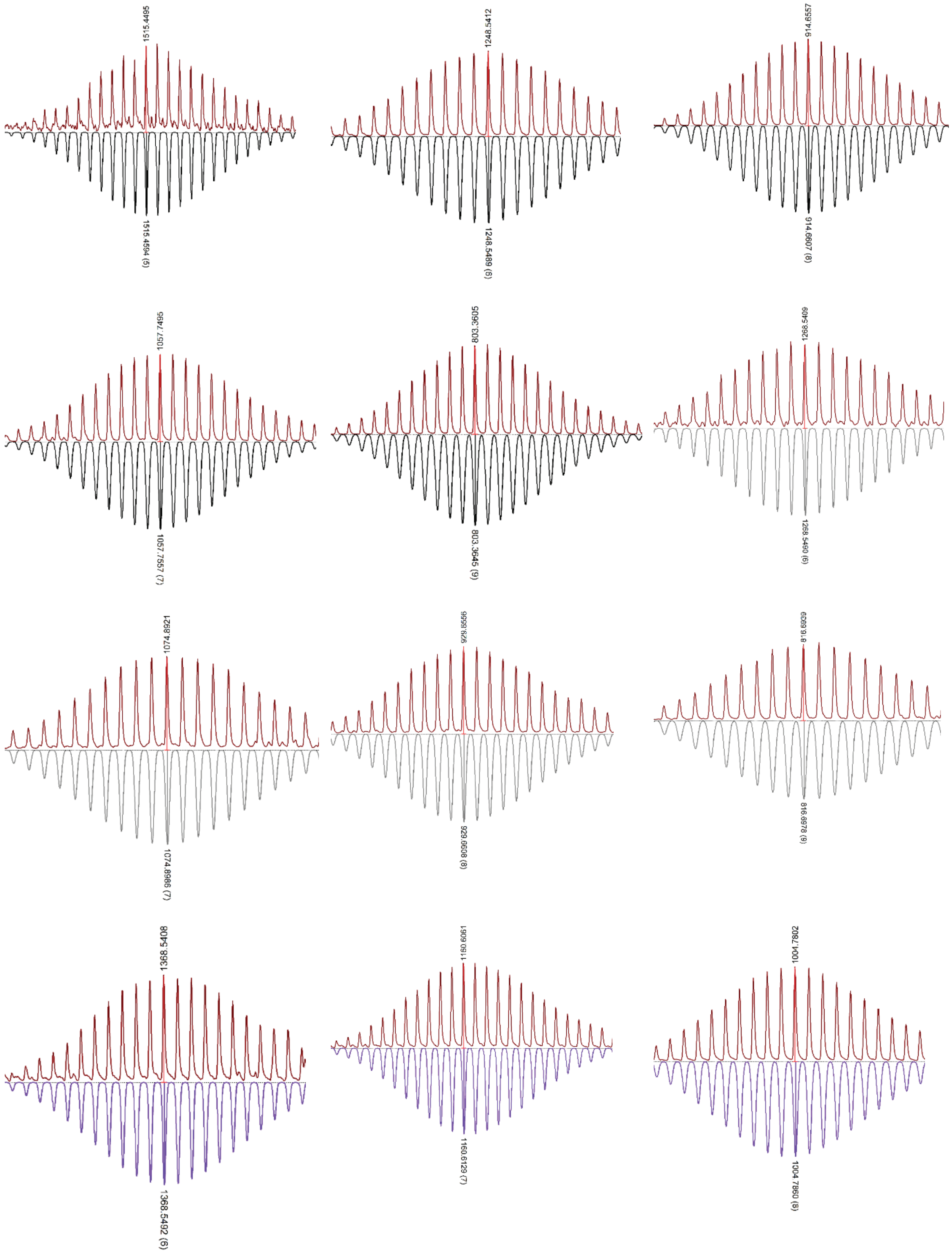

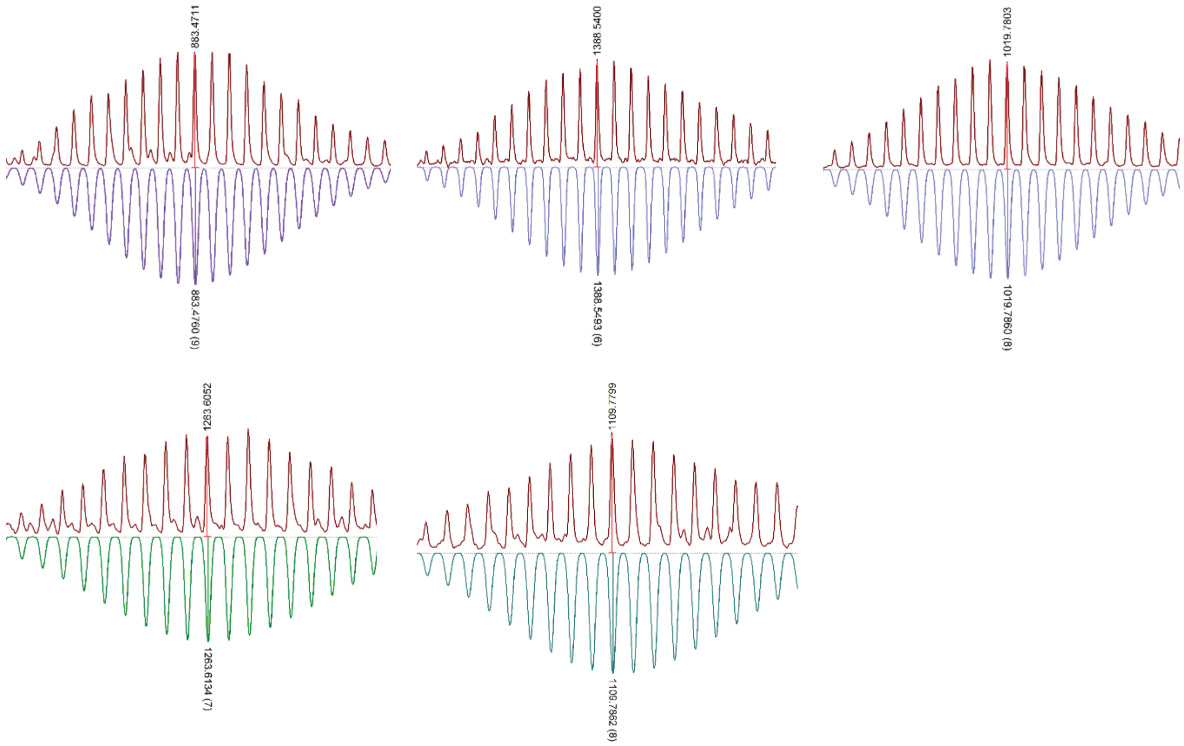

UV/VIS studies

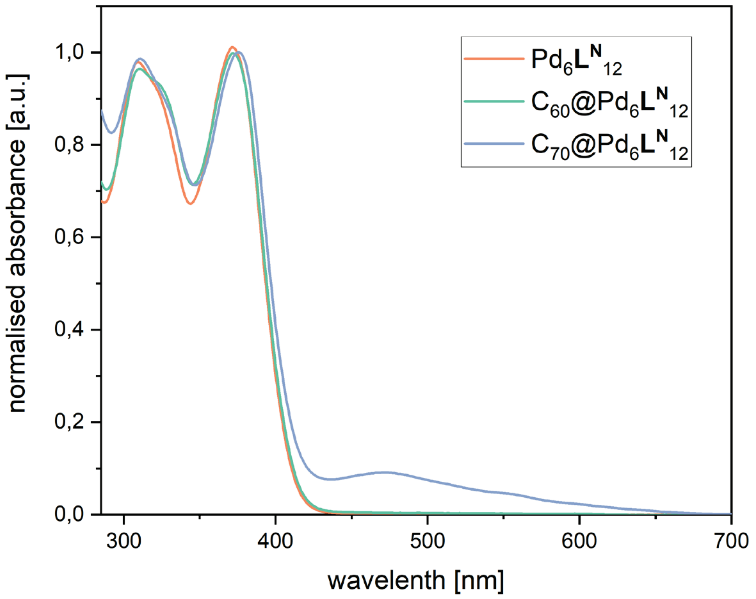

Figure S50. Normalized UV/VIS spectra of $Pd_6\mathbf{L}^{\mathbf{N}}_{12}$, $C_{60}\subset Pd_6\mathbf{L}^{\mathbf{N}}_{12}$ and $C_{70}\subset Pd_6\mathbf{L}^{\mathbf{N}}_{12}$.

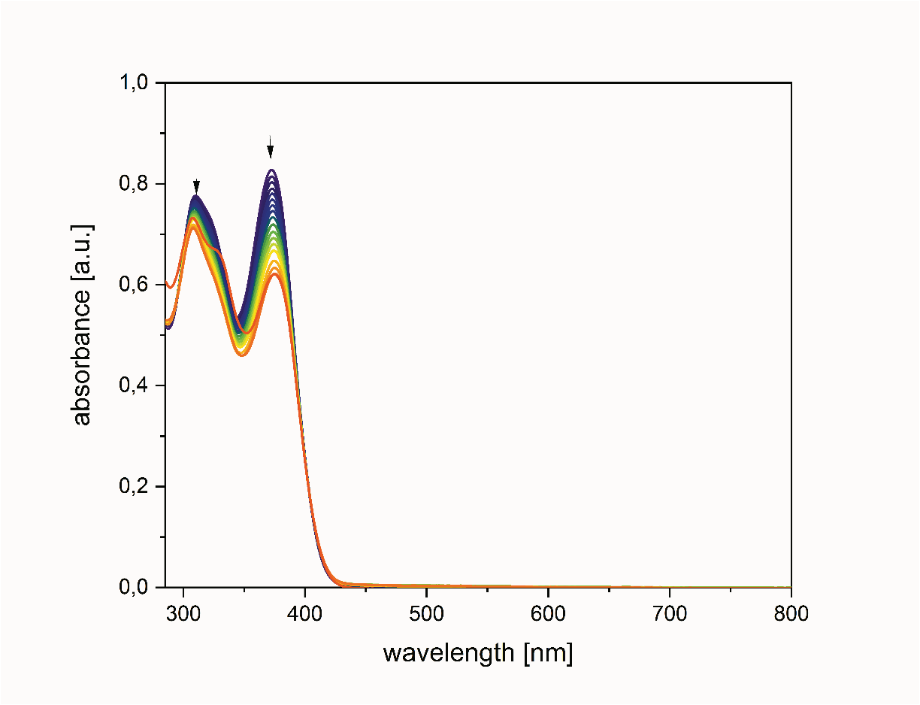

Figure S51. UV/VIS spectra of $Pd_6\mathbf{L}^{N}_{12}$ upon addition of C_{60}.

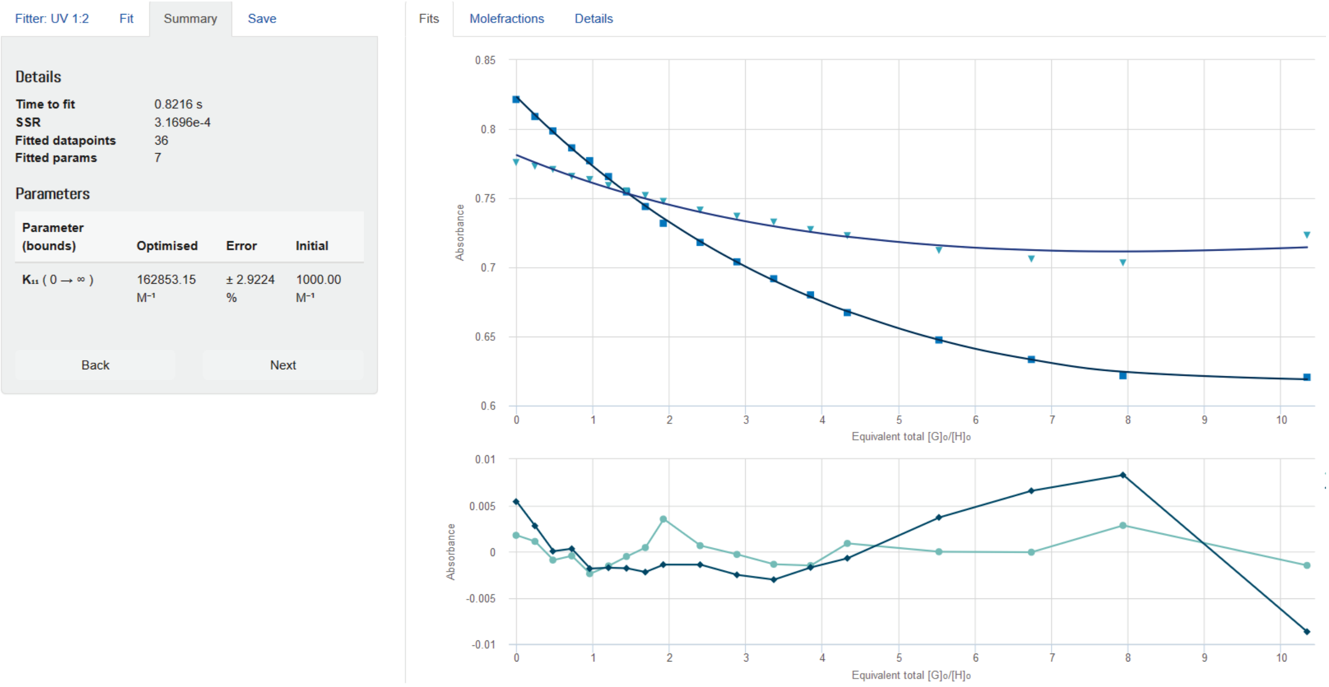

Figure S52. Fitting to obtain binding constant between C_{60} and $Pd_6\mathbf{L}^{N}_{12}$ (375 nm).

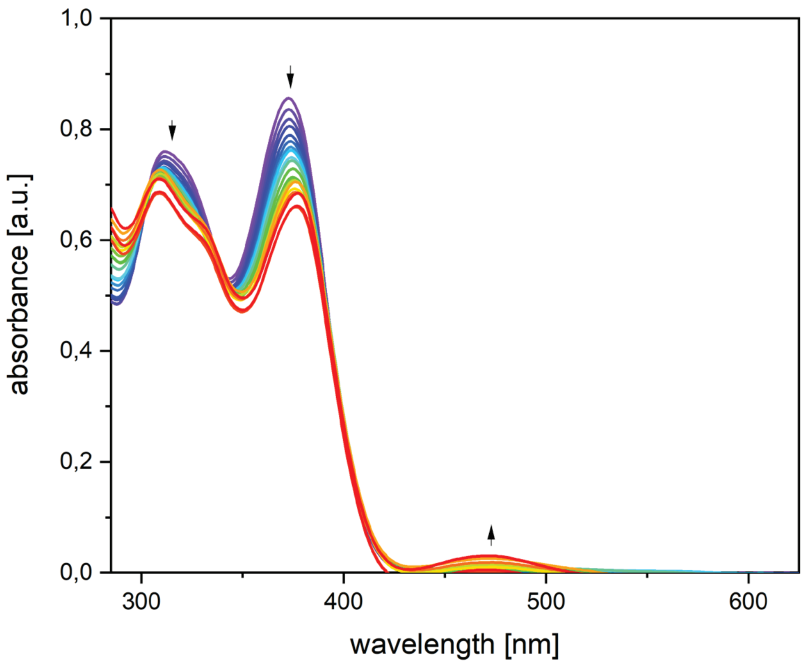

Figure S53. UV/VIS spectra of $Pd_6\mathbf{L}^{\mathbf{N}}_{12}$ upon addition of C_{70}.

<u>CV studies</u>

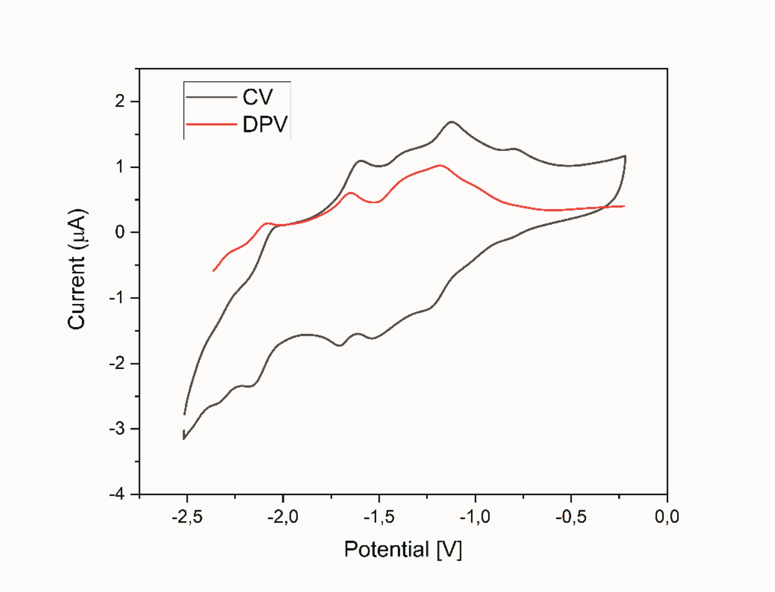

Figure S54. Reductive behavior of $C_{70}\subset Pd_6\mathbf{L}^{\mathbf{N}}_{12}$ in dmso by cyclic voltammetry (black) and by differential pulse voltammetry (red). V versus Fc/Fc^{+}.

Fullerene Binding $Pt_6L^{PEGPy}{}_{12}$ (S6)

<u>MS studies</u>

Table S55. Analysis of $C_{70}\subset Pd_6\mathbf{L}^{PEGPy}{}_{12}$ MS spectrum. Displayed are the counts for different amount of fullerene bound to the assembly and the resulting relative abundancy of the corresponding species.

Equiv. Fullerene	Counts	Relative abundance [%]
0	0	0
1	4561	42
2	4571	42
3	1707	16
4	0	0
5	0	0

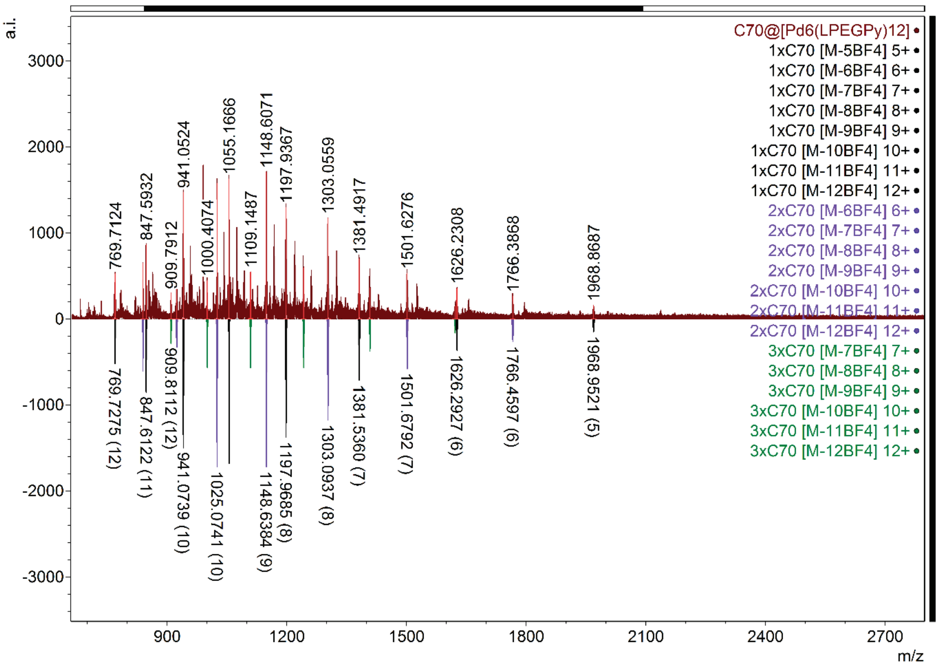

Figure S56. Full ESI-MS spectra of $C_{70}\subset Pd_6\mathbf{L}^{PEGPy}{}_{12}$. Below the simulated spectra, above obtained spectra.

Table S57. Zoom into ESI-MS spectra of $xC_{70}\subset Pd_6\mathbf{L}^{PEGPy}{}_{12}$ for x=1-3 (below the simulated spectra, above obtained spectra).

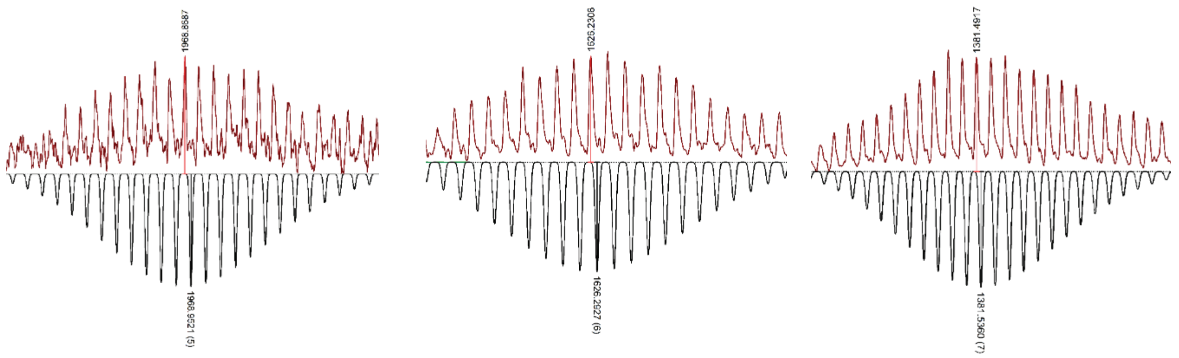

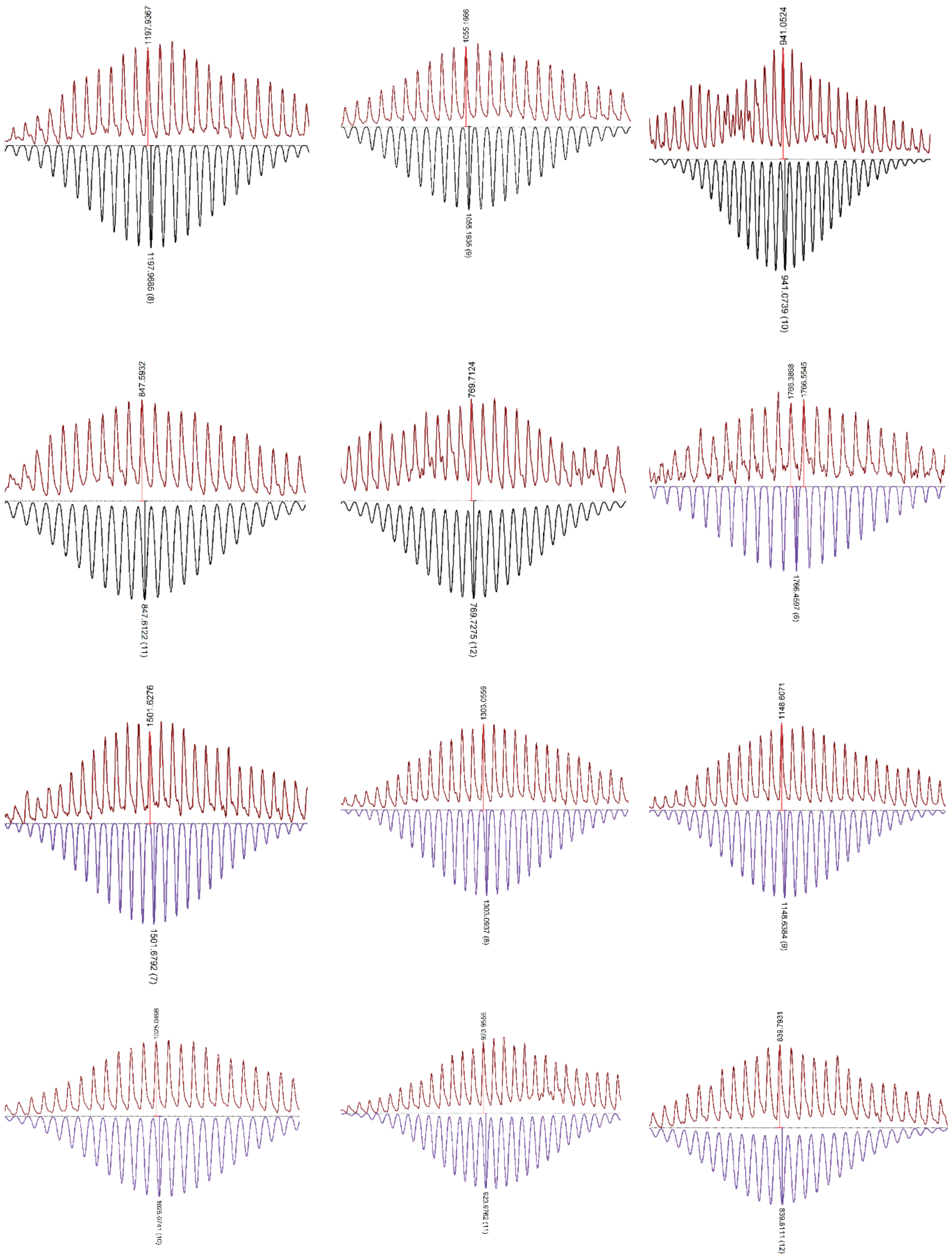
1197.9367
1197.9685 (8)
1055.1666
1055.1936 (9)
941.0524
941.0739 (10)
847.5932
847.6122 (11)
769.7124
769.7275 (12)
1766.3868
1766.5545
1766.4597 (6)
1501.6276
1501.6792 (7)
1303.0559
1303.0837 (8)
1148.6071
1148.6384 (9)
1025.0498
923.9555
839.7931
839.8111 (12)

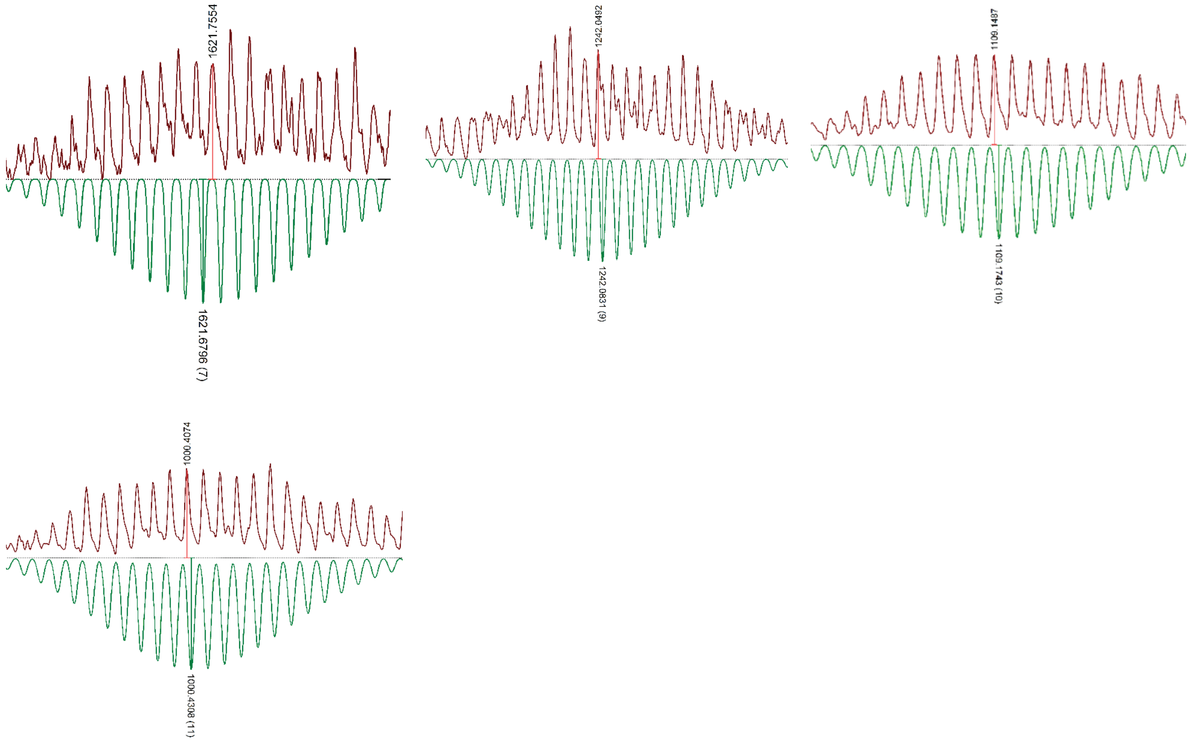

UV/VIS studies

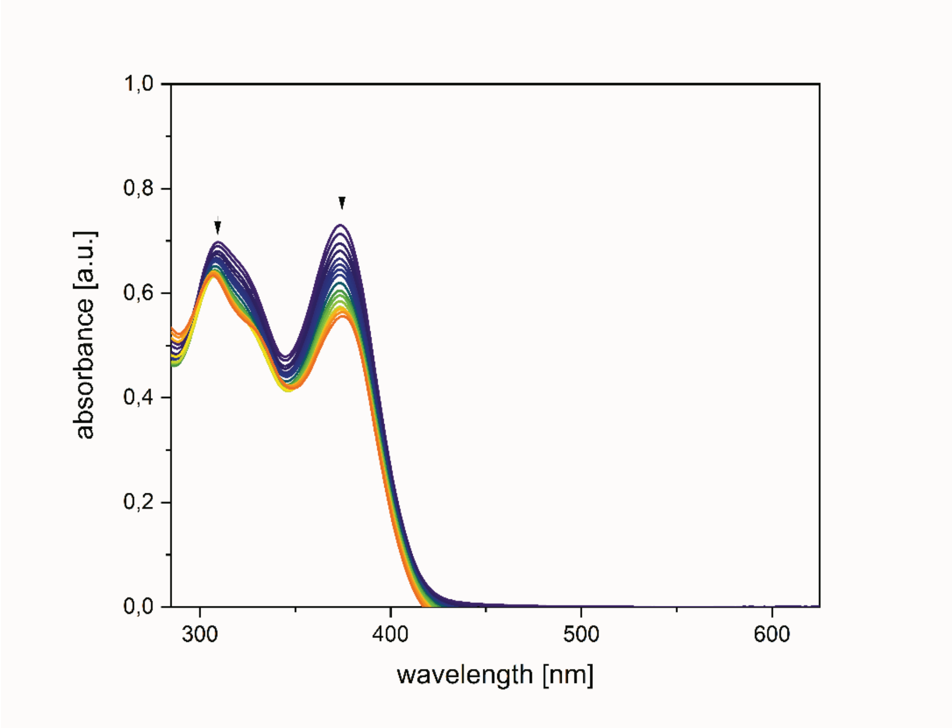

Figure S58. UV/VIS spectra of $Pd_6\mathbf{L}^{PEGPy}{}_{12}$ upon addition of C_{70}.

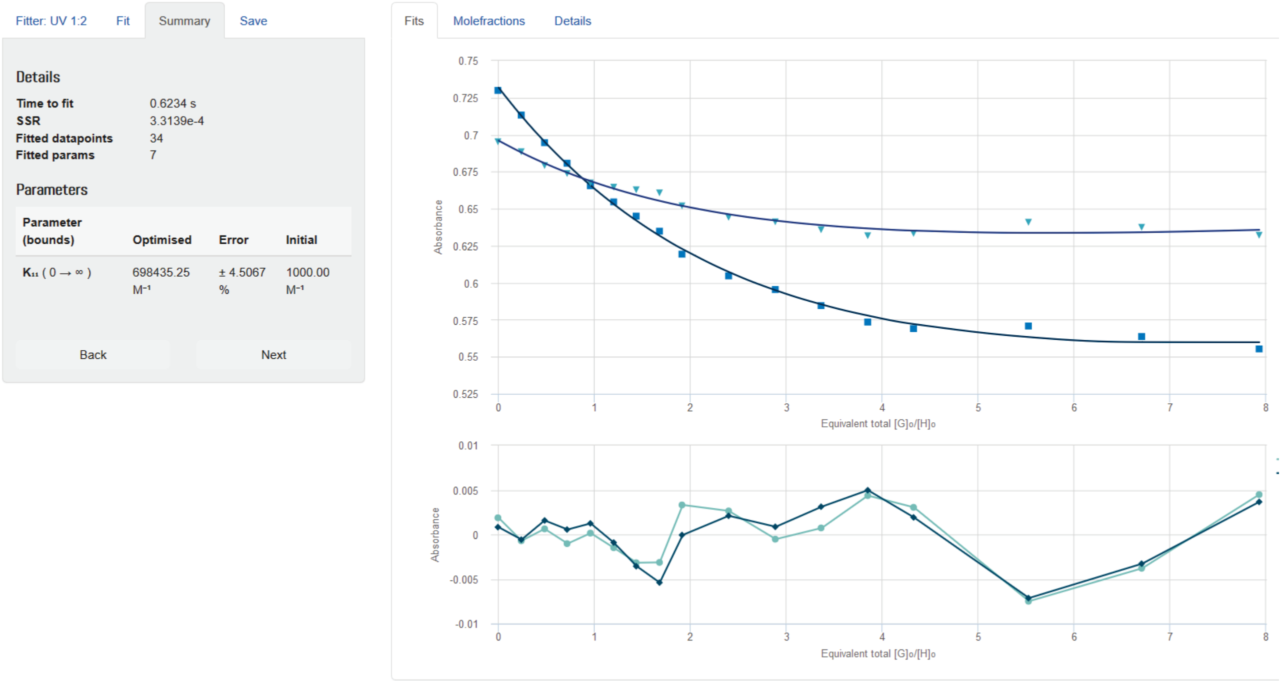

Figure S59. Non-cooperative 2:1 G:H fitting to obtain binding constant between C_{70} and $Pd_6\mathbf{L}^{\mathbf{PEGPy}}_{12}$ (375 nm and 308 nm).

Catalytic $^1O_2$ formation (S7)

General procedure: To solutions/suspensions of 10-20 μmol of substrate in 1 mL of the corresponding deuterated solvent, 5 ml of 0.83 mM solution of the corresponding sphere or sphere-fullerene complex in dmso-d_6 was added. The quartz tubes were placed 2 cm away from the light source and irradiated for 4 h at room temperature. Afterwards, mesitylene was added as standard and the yields of product determined by ^{1}H-NMR.

For control reactions using pure C_{70}, either solid C_{70} or solutions of C_{70} in toluene were added to 1 ml of the solvent of choice (see main text) and the resulting solutions were stirred for overnight. Thereafter, the solutions/suspensions were filtered through a syringe filter and applied in the catalysis.

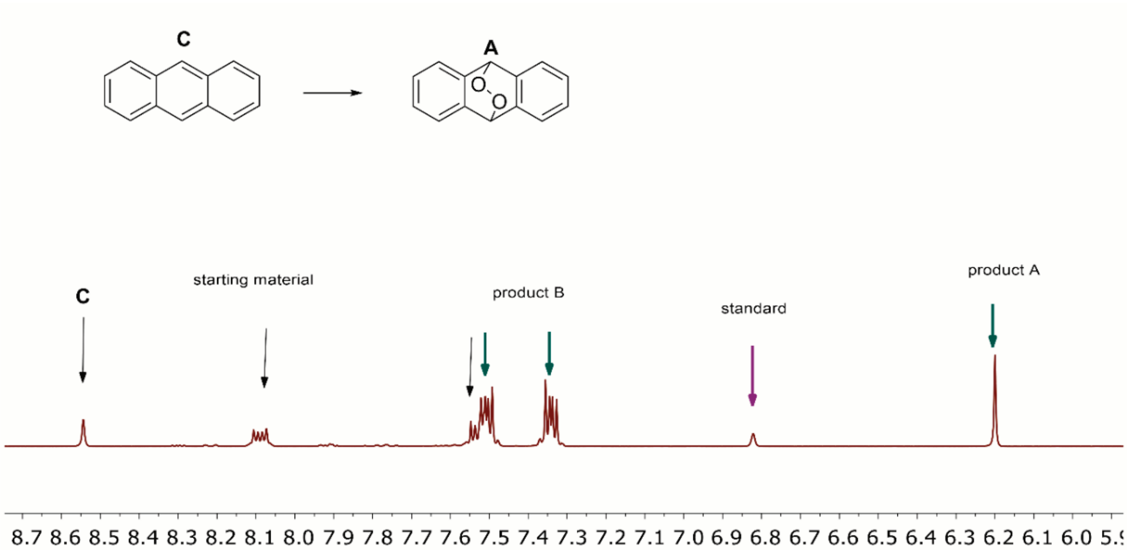

Figure S60. Anthracene oxidation in MeCN.

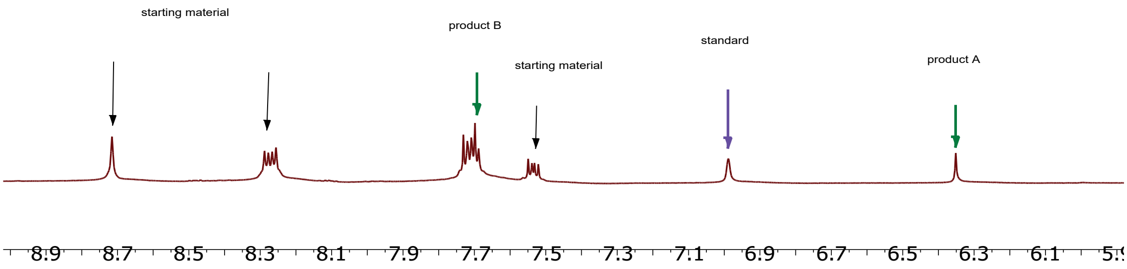

Figure S61. Anthracene oxidation in $MeNO_2$.

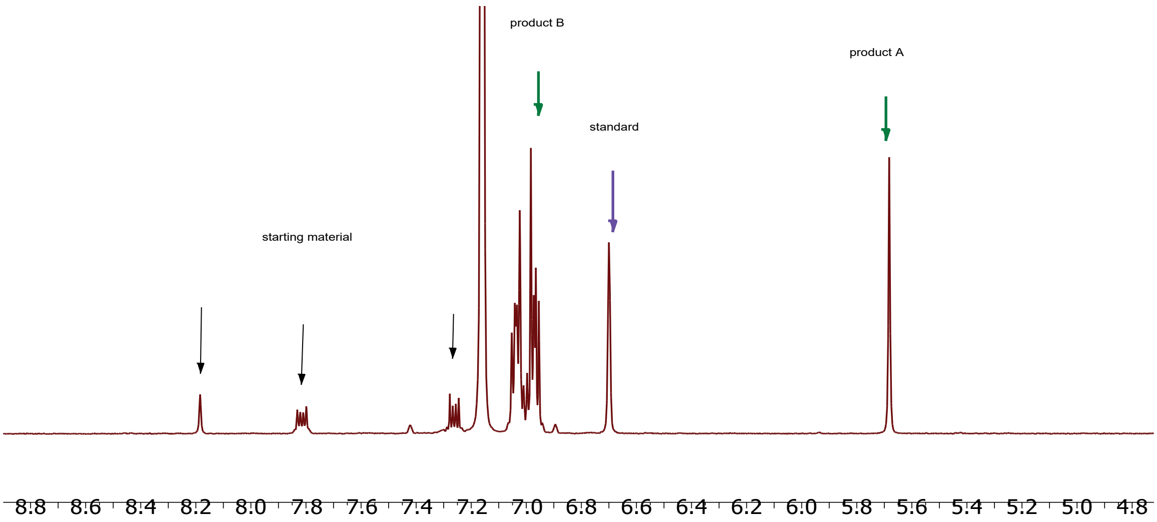

Figure S62. Anthracene oxidation in benzene.

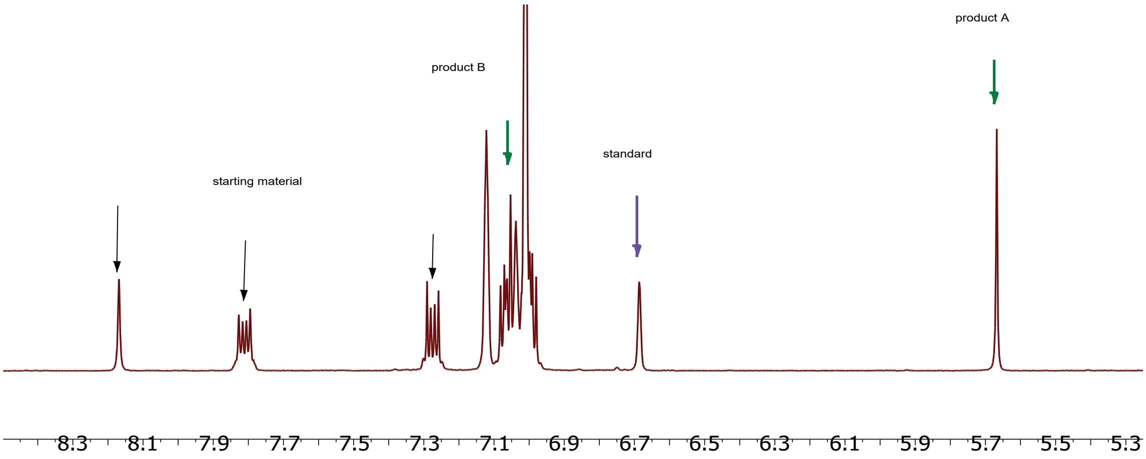

Figure S63. Anthracene oxidation in toluene.

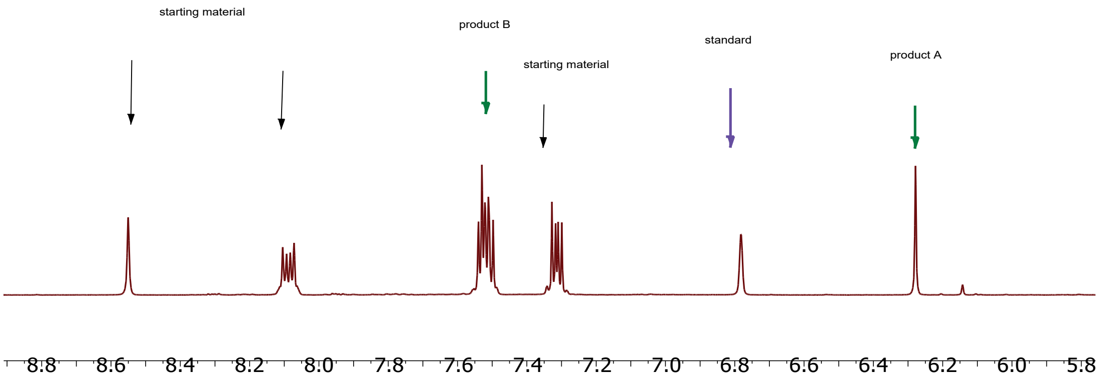

Figure S64. Anthracene oxidation in acetone.

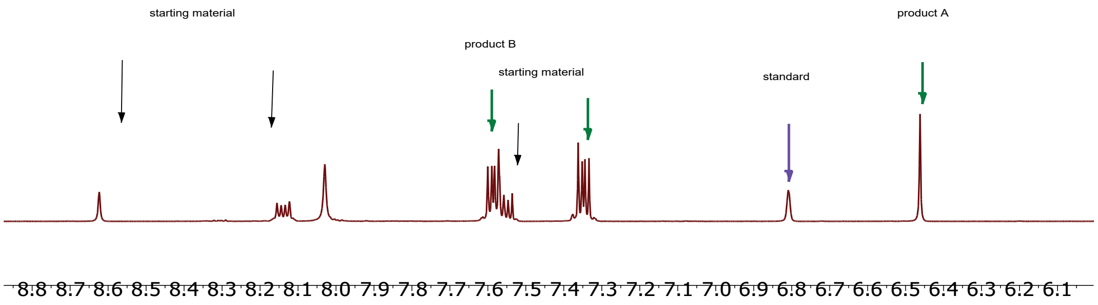

Figure S65. Anthracene oxidation in dmf.

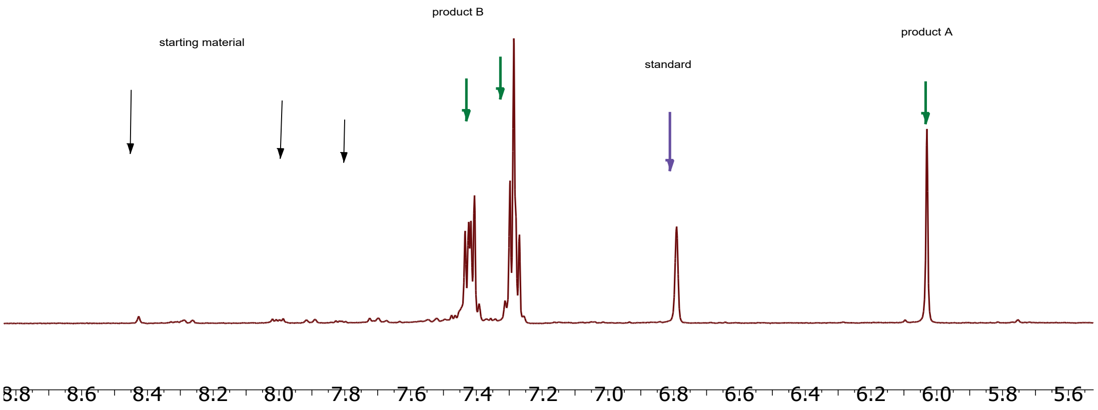

Figure S66. Anthracene oxidation in chloroform.

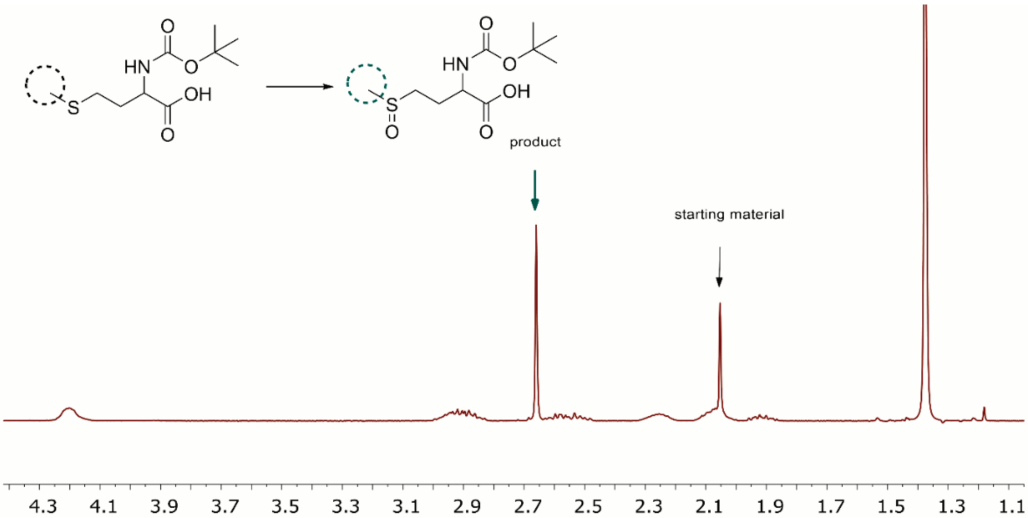

Figure S67. Boc-Met oxidation in D_2O.

Oxidation of other substrates: All other substrates were oxidized according to the general procedure in MeCN-d_3. ^{1}H-NMR spectra are in agreement to literature.

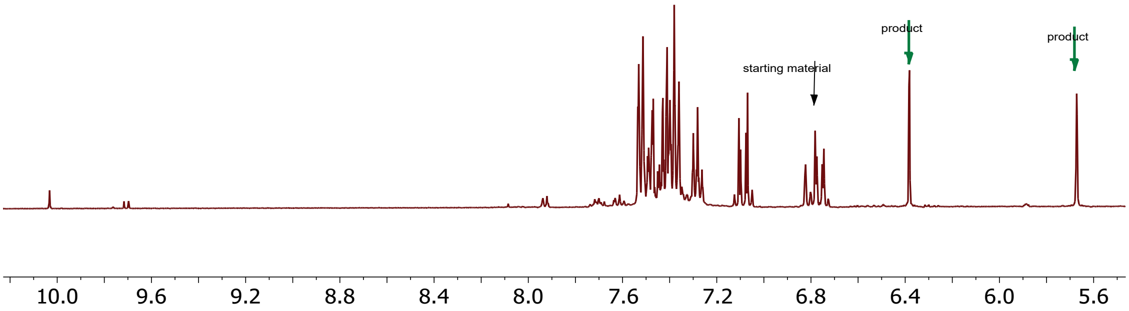

Figure S68. Selected region of the ^{1}H-NMR spectra of the photocatalytic peroxidation of 1,4-diphenylbuta-1,3-diene.

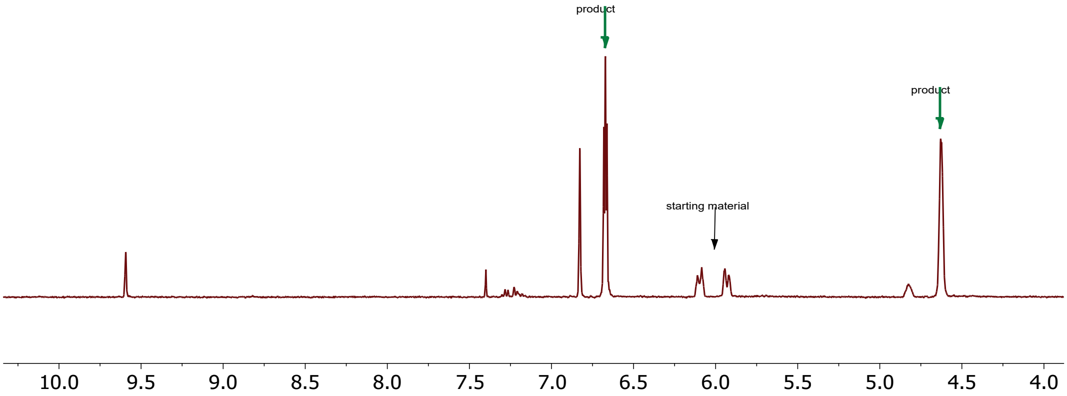

Figure S69. Selected region of the ^{1}H-NMR spectra of the photocatalytic peroxidation of cyclohexadiene.

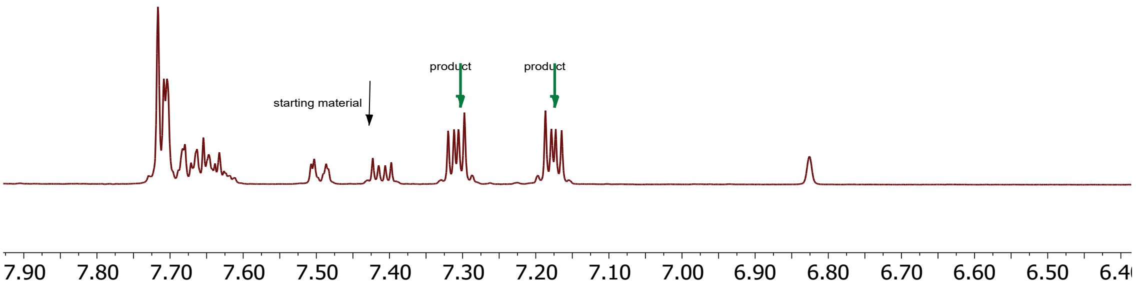

Figure S70. Selected region of the ^{1}H-NMR spectra of the photocatalytic peroxidation of diphenyl anthracene.

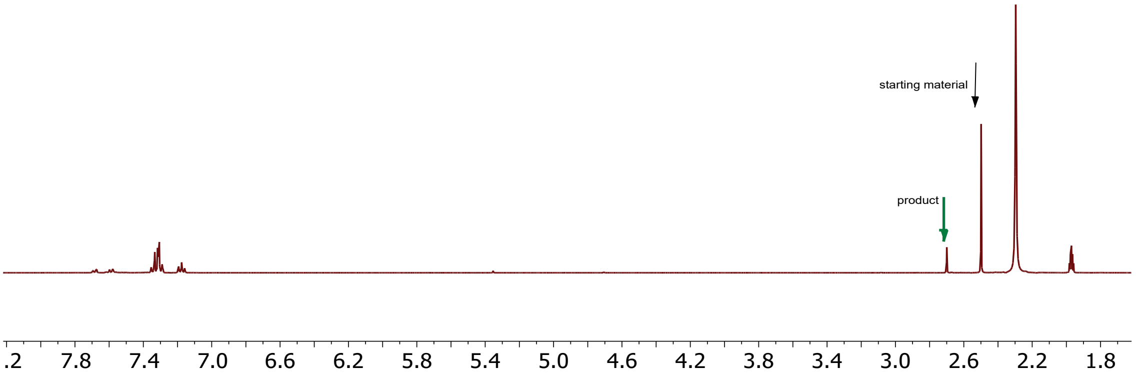

Figure S71. Selected region of the ^{1}H-NMR spectra of the photocatalytic peroxidation of methyl(phenyl)sulfane.

MS analysis evaluation (S8)

Mass analysis performed on the fullerene containing nanospheres gives a distribution of different signals which can be assigned to different amounts of fullerene bound to the nanospheres (e.g., Fig. S43 and Table S44). The relative distribution in solution may differ from the MS experiments as the MS experiments are generally performed in the gas phase. Furthermore, ionizability of different species (e.g., spheres bearing one fullerene versus spheres bearing 4 fullerenes) may proceed in a non-linear correlated fashion, changing the overall distribution in MS analysis versus in solution. In an attempt to get a better picture how the MS distribution analysis and in solution distribution correlate, two sets of experiments were set up.

In the first experiment exactly 1 equivalent of solid C_{70} was added to a solution of $[Pd_6\mathbf{L}^{\mathbf{N}}_{12}]$ and stirred for 24 h. Thereafter, all C_{70} was dissolved and the solution was analyzed with MS. Analysis of the MS data revealed signals corresponding to 0-3 $C_{70}\subset[Pd_6\mathbf{L}^{\mathbf{N}}_{12}]$ (Fig. S72 and S73). The calculated avarage C_{70} amount bound to a single sphere was calculated according to:

$$\boldsymbol{N_{C70}} = \frac{\mathbf{0} \times \boldsymbol{I_{0C70}} + \mathbf{1} \times \boldsymbol{I_{1C70}} + \mathbf{2} \times \boldsymbol{I_{2C70}} + \mathbf{3} \times \boldsymbol{I_{3C70}} + \mathbf{4} \times \boldsymbol{I_{4C70}}}{\boldsymbol{I_{0C70}} + \boldsymbol{I_{1C70}} + \boldsymbol{I_{2C70}} + \boldsymbol{I_{3C70}} + \boldsymbol{I_{4C70}}}$$

With N_{C70} being the avarege number of C_{70} being bound to a nanosphere and $I_{0\text{-}4C70}$ being the number of counts for 0-4 C_{70} associated to a sphere in MS analysis. The following values were obtained: I_{0C70} = 13933 a.u.; I_{1C70} = 15365 a.u.; I_{2C70} = 6280 a.u.; I_{3C70} = 1422 a.u., yielding an average number of C_{70} bound to a sphere of N_{C70} = 0.9. This number is 10% off the theoretical $N_{C70}^{theoretical}$ = 1 which is expected as excactly 1 equivalent C_{70} was added to the solution. This experiment shows that the MS analysis reflects well the amount of C_{70} being present is solution with a 10% underrepresentation of C_{70} in MS analysis versus solution. This underrespresentation can be derived to different ionizability and/or fragmentation of certain species during the MS measurement. This experiment furthermore highlights the preferance of nanospheres to bind multiple fullerene C_{70} more favorably then single fullerenes. Although, only one equivalent of C_{70} was added to the solution, substential intensity of two and three fullerenes bound to a single nanosphere were found (see also disscussion in main text Figure 6).

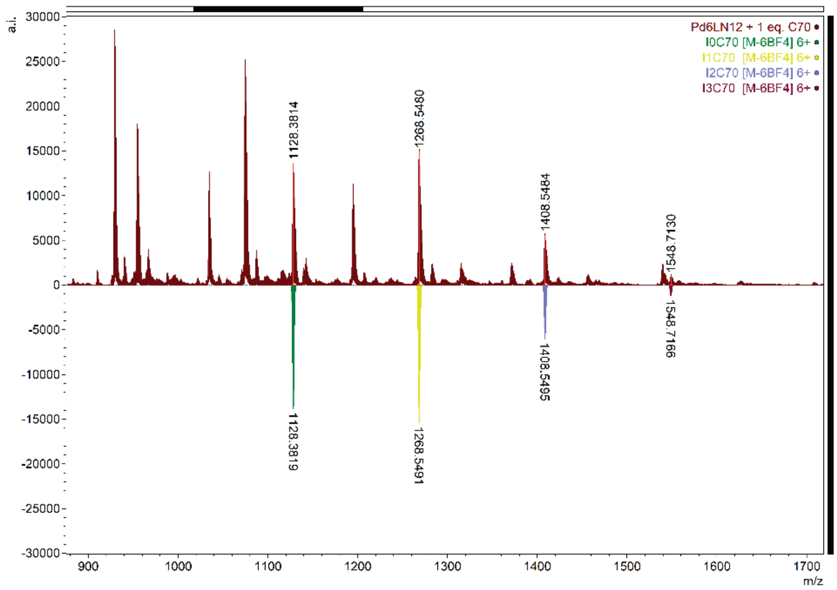

Figure S72. Full ESI-MS spectra of $1C_{70}$⊂$Pd_6\mathbf{L}^{\mathbf{N}}_{12}$. Below the simulated spectra, above obtained spectra.

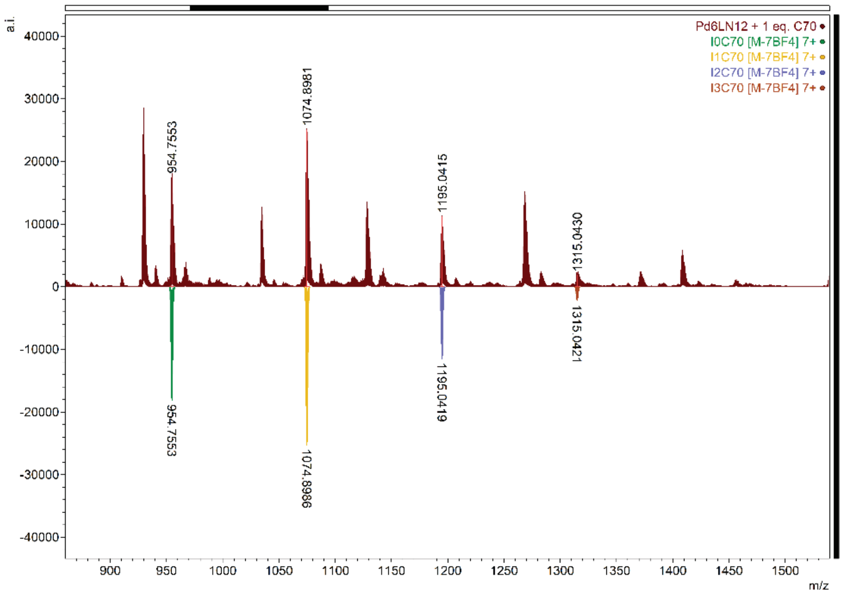

Figure S73. Full ESI-MS spectra of $1C_{70}$⊂$Pd_6\mathbf{L}^{\mathbf{N}}_{12}$. Below the simulated spectra, above obtained spectra.

In a second set of experiments, we added 5 eq. solid [6,6]-Phenyl C_{61} butyric acid methyl ester (Fig. S74) to a solution of [$Pd_6\mathbf{L}^{\mathbf{N}}_{12}$], in dmf, stirred the suspension for 24 h at room temperature, filtered the suspension and analysed the composite with ^{1}H-NMR and MS. NMR analysis displayed on average 3 C_{61} being associated with a single nanosphere (Fig. S71). According to the above described formula, we calculated via MS analysis (Fig. S75). The following values were obtained: I_{0C61} = 0 a.u.; I_{1C61} = 20251 a.u.; I_{2C61} = 38021 a.u.; I_{3C61} = 34362 a.u.; I_{4C61} = 16716 a.u.; I_{5C61} = 4218 a.u. yielding an average number of C_{61} bound to a sphere of N_{C61} =2.6 (according to MS analysis) which again is ~10% lower than the expected value of the solution analysis ($N_{C61}^{solution}$ = 3).

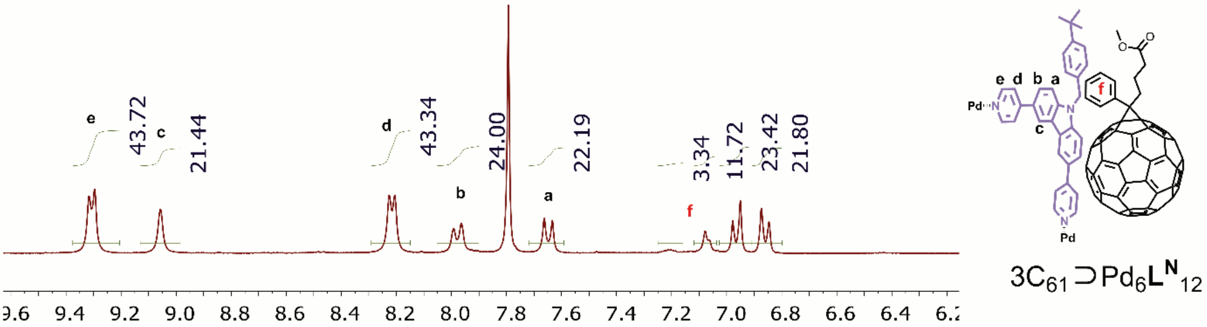

Figure S74. $C_{61}{\subset}[Pd_6(\mathbf{L^N})_{12}]$ composite, ^{1}H-NMR in dmf with assignment of the signals corresponding to the sphere framework and to the functionalized fullerene (right).

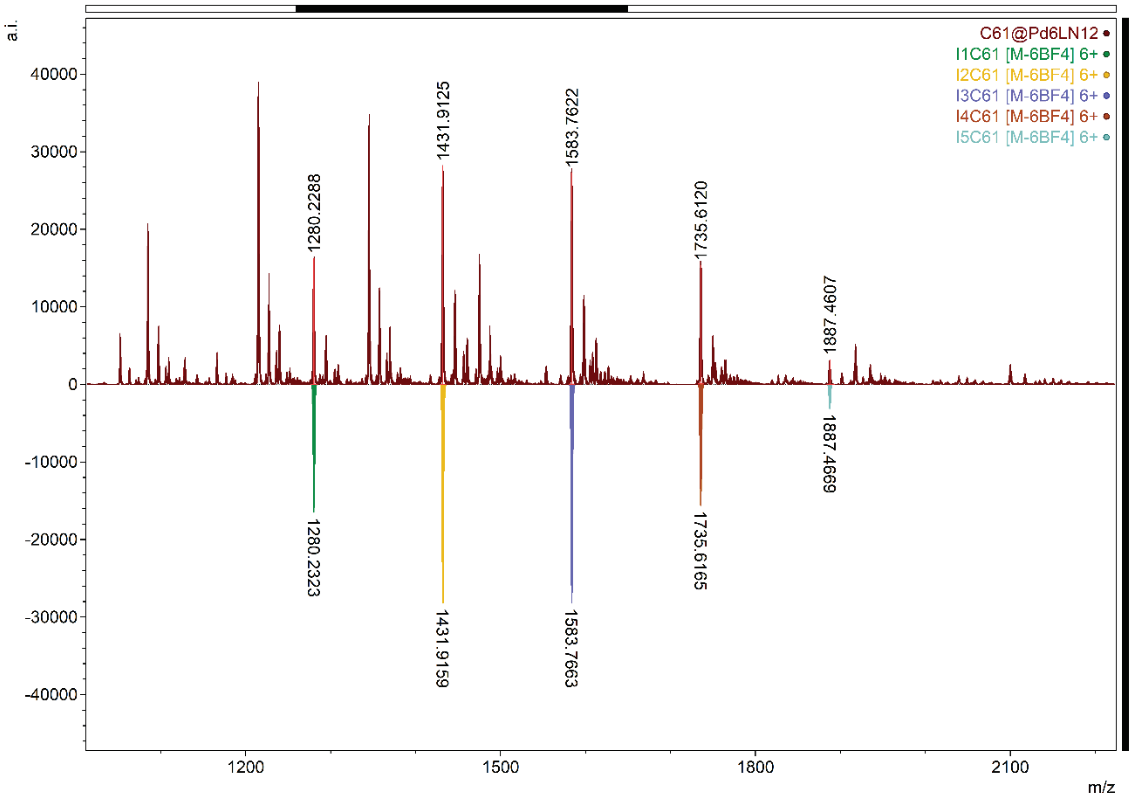

Figure S75. Full ESI-MS spectra of $C_{61}{\subset}Pd_6\mathbf{L^N}_{12}$. Below the simulated spectra, above obtained spectra.

The lower average amount of fullerenes found bound to single assemblies using MS analysis was further investigated using MSMS. When a species bearing one or two fullerenes was exposed to higher collision energies, we found fragmentation of fullerene containing species to the empty cage (Fig. S76 and Fig. S77). This fragmentation of the fullerene containing species is anticipated to yield the lower overall counts of the average number of fullerenes to single nanospheres.

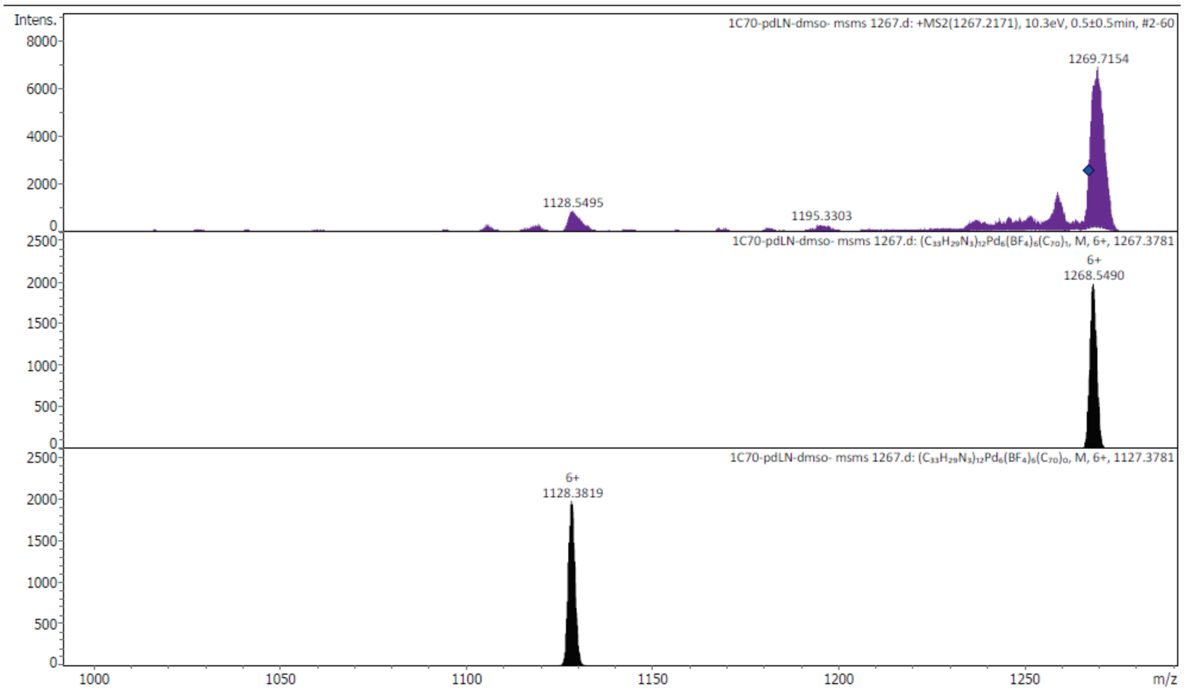

Figure S76. MSMS of $1C_{61}{\subset}Pd_6\mathbf{L}^{\mathbf{N}}_{12}$ (1268 Da), yielding the empty $Pd_6\mathbf{L}^{\mathbf{N}}_{12}$ (1128 Da) (below the simulated spectra, above obtained spectra).

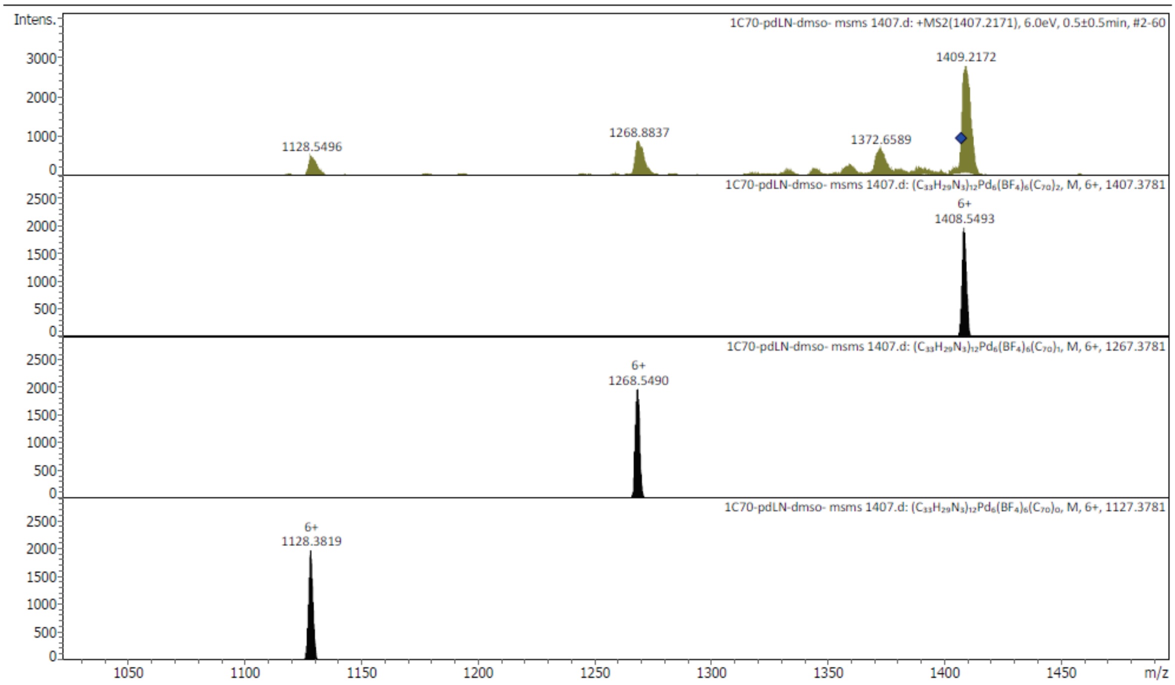

Figure S77. MSMS of $2C_{61}{\subset}Pd_6\mathbf{L}^{\mathbf{N}}_{12}$ (1408 Da), yielding $1C_{61}{\subset}Pd_6\mathbf{L}^{\mathbf{N}}_{12}$ (1268 Da) and the empty $Pd_6\mathbf{L}^{\mathbf{N}}_{12}$ (1128 Da) (below the simulated spectra, above obtained spectra).

Fullerene localization (S9)

Because atempts to obtain single crystals for X-Ray structure determination were unsuccesfull using a variety of solvent combinations (sphere-fullerene composites in either dmso, dmf or dmac, slow vapour diffusion of different anti-solvents such as diethylether, di-isopropyl-ether, ethylacetate, THF, chloroform, DCM), we focused the structural analysis on computational methods presented in the main text (Fig. 6). To further support the anticipated window-binding motiv, additional experiments were

performed which are described and summarized werein. In principle, inside of the sphere binfing (Figure S78A) or window binding is possible (Figure S78B). To distinguish between the two scenarios, we performed ^{1}H-NMR titration experiments.

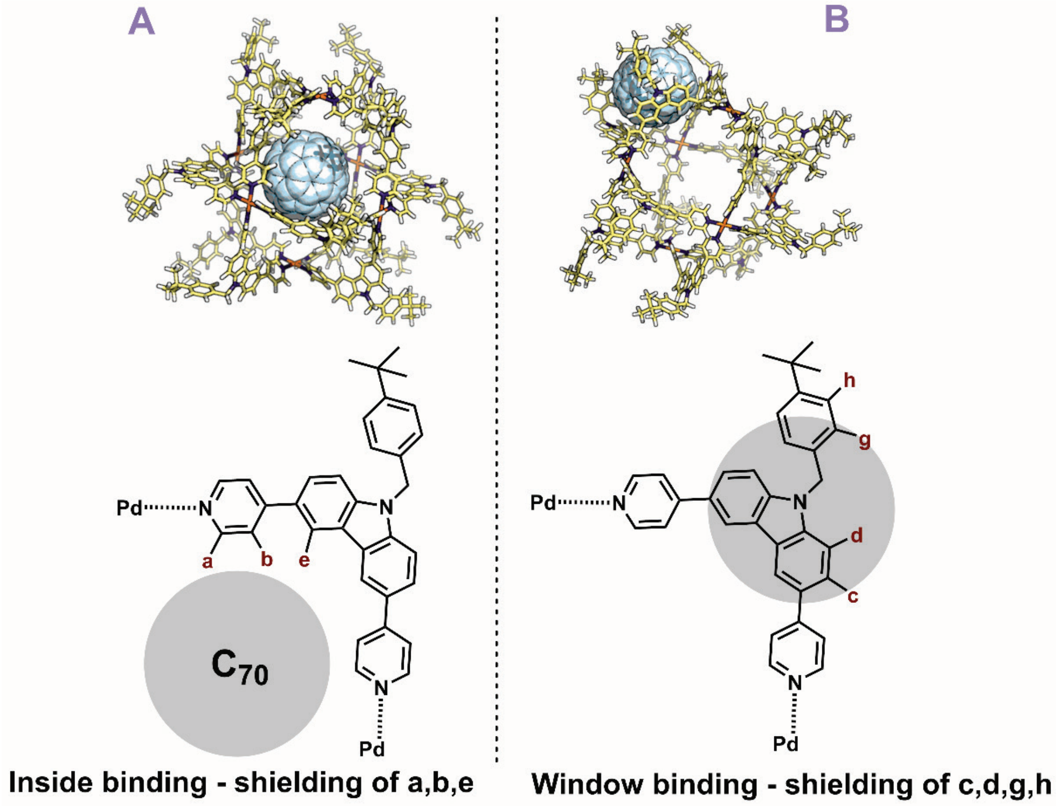

Figure S78. Influence of C_{70} binding for proton resonances at (A) inside binding of C_{70} and (B) window binding mode.

The proximity of C_{70} guests perturbs the magnetic environment of ^{1}H nuclei leading to changes in the chemical shift. A series samples of $Pt_6\mathbf{L}^{\mathbf{N}}_{12}$ cages (0.833 mM) with 0.00, 0.50, 0.75, 1.00, 1.50, 2.00 and 4.00 equivalents of C_{70} guests prepared in DMSO-d_6 to investigate the localization of guests. Ordinary ^{1}H – NMR spectra were obtained for each sample with a Bruker AVX 500 NMR (^{1}H = 500 MHz) at 300 K, where distinct changes in the chemical shifts were observed (Figure S79). At each titration level we observed a single peak corresponding to all ^{1}H nuclei in the absence and presence of C_{70} (Figure S80). Using the largest chemical shift difference observed for $Pt_6\mathbf{L}^{\mathbf{N}}_{12}$ ($\Delta\delta_c$ = 0.09 ppm) we infer that the C_{70} transfers between $Pt_6\mathbf{L}^{\mathbf{N}}_{12}$ cages and corresponding binding sites with a lifetime of less than 10 ms,[6] and thus cannot be distinguished by ^{1}H–NMR spectroscopy.

We observed that the most outward nuclei of the $Pt_6\mathbf{L}^{\mathbf{N}}_{12}$ cage (i.e., H_c , H_f, H_g, and H_h) shift upfield with increasing C_{70} concentration suggestive of π-interactions, shielding. This shift of the outer protons of the sphere is only agreeable with a window binding mode (Figure S78 B) as inside binding would only influence the interior protons (such as a,b,e; Figure S78 A). Moreover, these upfield shifts are more pronounced (Δδ = 0.033 – 0.093 ppm) than downfield shifts observed for other nuclei (Δδ = 0.010 – 0.048 ppm). We surmise that these chemical changes arise from associated C_{70} guests which reside proximately to these nuclei located at the windows of the sphere, in agreement with our computational simulations.

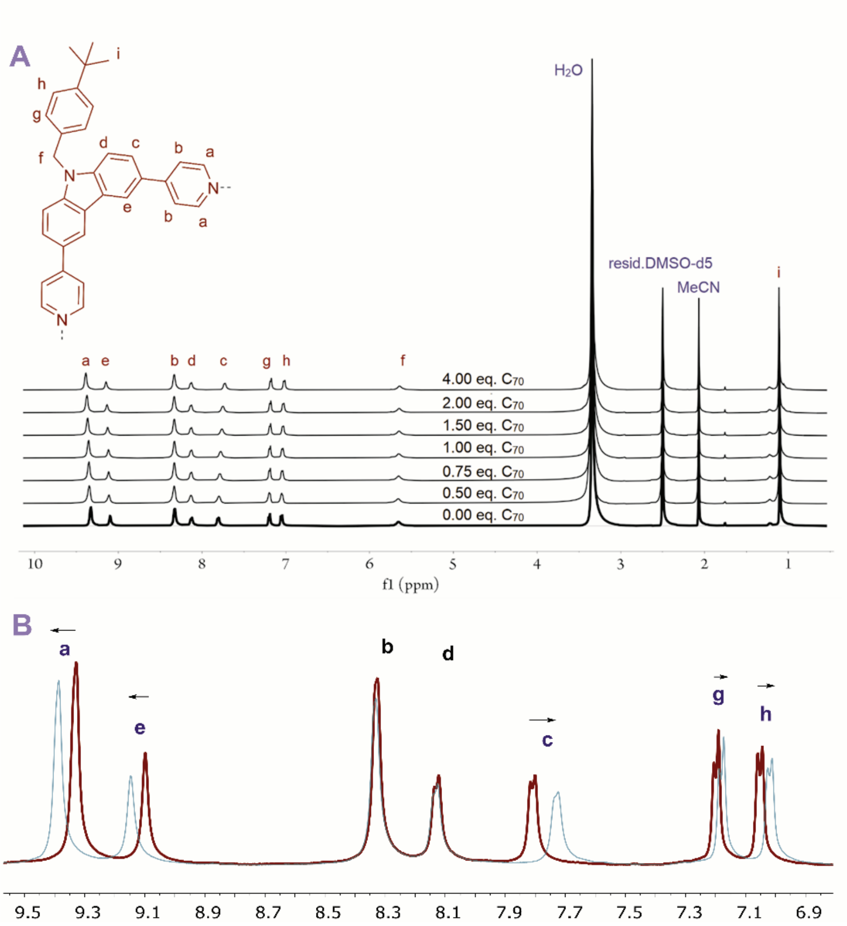

Figure S79. (A) A series of ^{1}H – NMR spectra obtained for Pt_6**L**$^{\mathbf{N}}{}_{12}$ coordination cages (0.833 mM) prepared with 0.00 to 4.00 equivalents of C_{70} guests in DMSO-d_6 (^{1}H = 500 MHz, T_r = 5 s). Chemical shifts were referenced from the solvent residual DMSO$_{\text{-d6}}$ ($\delta_{\text{resid DMSO-d6}}$ = 2.500 ppm).[5] Trace quantities of water (H_2O, δ_{H2O} = 3.333 ppm) and acetonitrile (MeCN, 2.071 ppm) remained as impurities from the preparation. (B) Zoom in into aromatic region of 0 equivalents of C_{70} (red) and 4 equivalents C_{70} (blue).

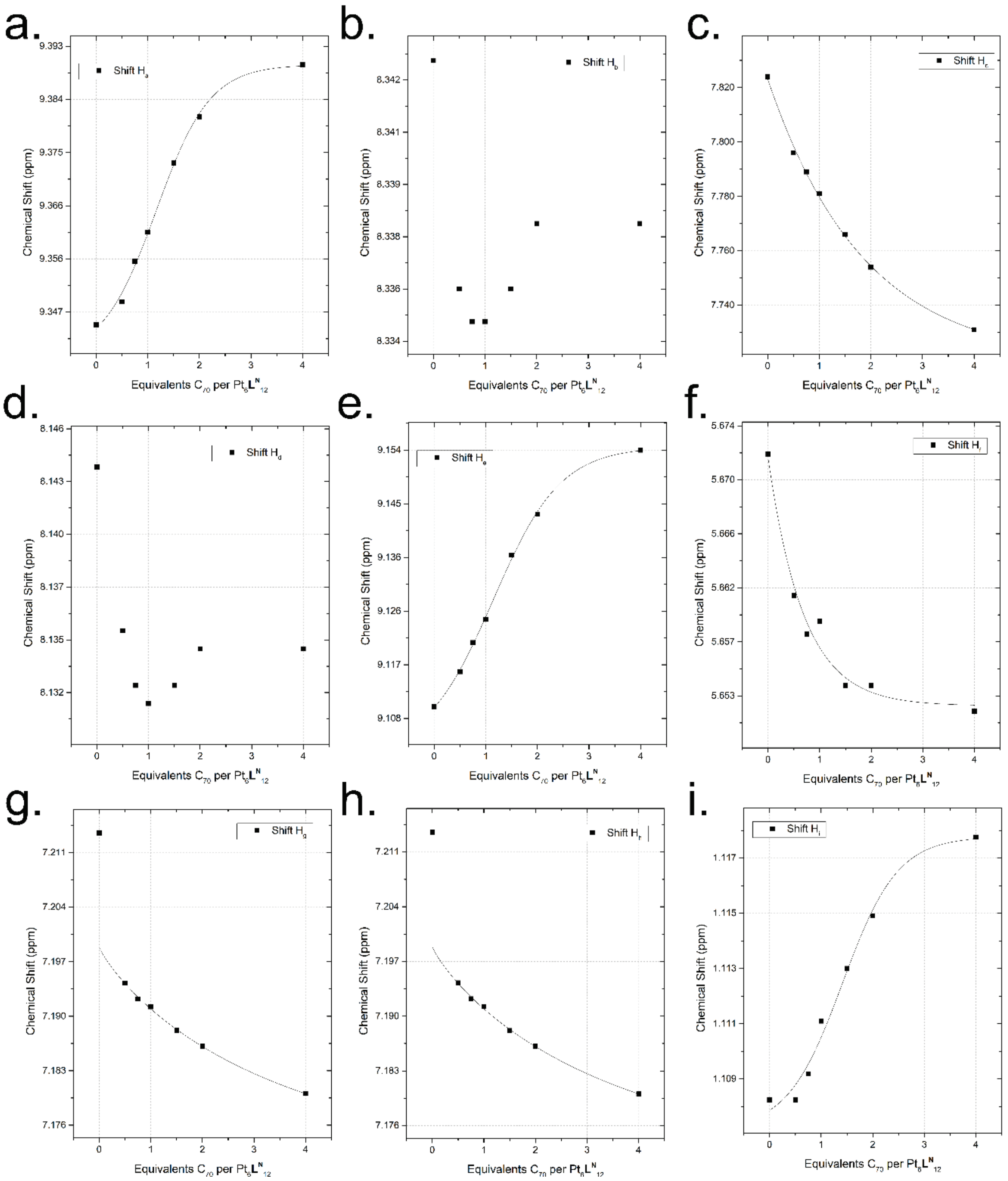

Figure S80. Chemical shifts changes for ^{1}H nuclei of $Pt_6L^N_{12}$ cages (0.833 mM, DMSO-d_6) with varying concentration of C_{70} (0.00 – 4.00 equivalents). Chemical shifts are referenced to residual DMSO-d_6 and graphs are identified by corresponding ^{1}H nucleus (Figure S76). Data are fit with a sigmoidal function (black trace) when appropriate to illustrate trends observed over the course of titration.

Modelled C_{70}-accesible internal volume of $Pt_6L^N_{12}$ cages

The accessible volume within the cage permits and limits the association of C_{70} guests. Using the structures generated from our MD studies we conducted a series of Connally accessible volume measurements with probe radii between 1.0 and 40.0 Å.[4] We observed that C_{70} guests (radius = 7.0 – 10.0 Å) is similar in size to the pores (i.e., windows) of $Pt_6L^N_{12}$. This result suggests that the transit of C_{70} through the pores of $Pt_6L^N_{12}$ may be sterically limited (Figure S81). Interestingly, for probe radii similar in size to C_{70} (6.0 – 8.0 Å) an internal cavity is identified by a Connally approach. This interior cavity possesses an volume of 2092.8 ± 428.1 Å^3, similar to a single C_{70} guest molecule. This suggests a single C_{70} guest may be encapsulated at the center of pores of the cage (although perturbation of the sphere structure is required to pass the windows) while further guest association can only occur at the pores (Figure 6, main text).

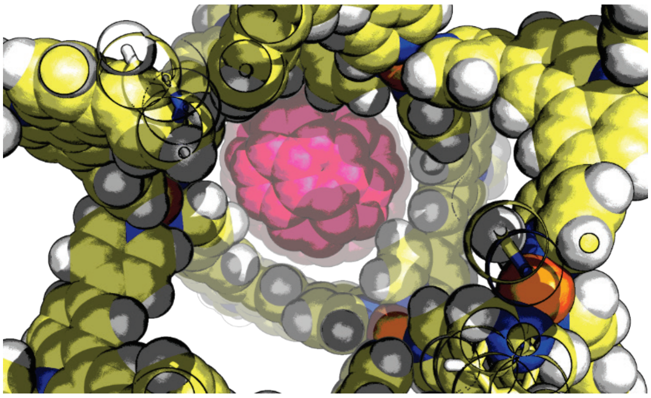

Figure S81. Space-filling model of a window and fullerene, showing that the window closing is of a comparable size as fullerene C_{70}.

Catalytic performance discussion (S10)

In the main text, we describe enhanced catalytic activity of the sphere-fullerene composites in comparison to free fullerene in a variety of solvents. Although, we anticipate that the majority of this enhancement is attributed to the enhanced solubility of fullerene by assembling the host-guest complexes, some interesting observations were made during the course of the experiments, which may be interesting for further investigation and are discussed herewith.

When solid anthracene (20 μmol) was added to 1 mL three different solutions of acetonitrile (containing 1: 5 μl dmso; 2: 5 μl $[Pd_6\mathbf{L^N}_{12}]$ in dmso; 3: 5 μl $C_{70}{\subset}[Pd_6\mathbf{L^N}_{12}]$ in dmso) we observed that initially more anthracene dissolved in the solutions containing the nanospheres then in the sample without nanospheres. This difference in initial solubilization of anthracene is documented by a comparison of the initial ^{1}H-NMR spectra which were taken directly after mixing the solutions (Fig. S82).

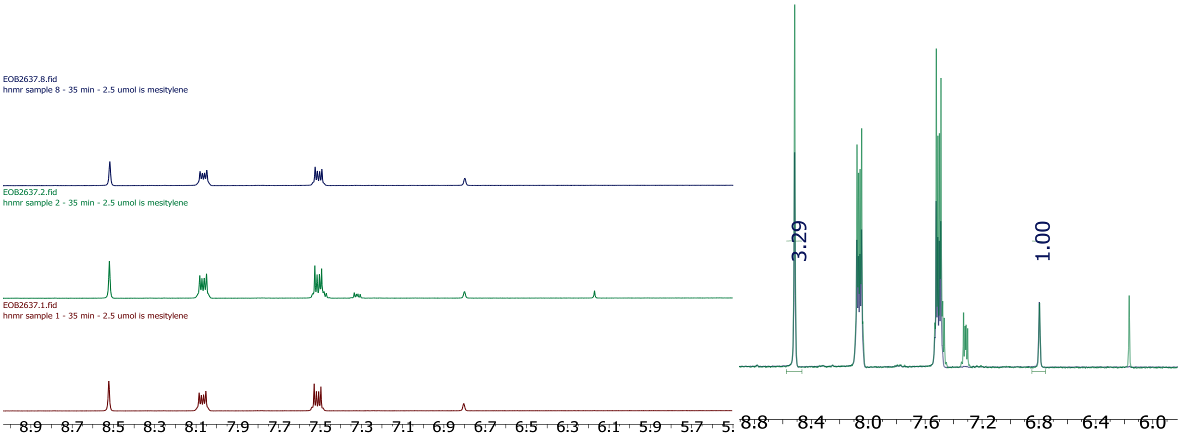

Figure S82. Left: Solid anthracene added to different solutions of MeCN containing dmso (top), $C_{70}{\subset}[Pd_6(\mathbf{L^N})_{12}]$ (middle) and $[Pd_6(\mathbf{L^N})_{12}]$ (bottom). Right: comparison of anthracene dissolved in MeCN containing dmso (blue) and $C_{70}{\subset}[Pd_6(\mathbf{L^N})_{12}]$ (green).

As described before, nanosphere-fullerene composites exhibit a hydrophobic π-rich pocket which can be utilized for guest encapsulation (main text Fig. 6). We anticipated that especially in polar solvents (such as MeCN) anthracene can act as a guest (or multiple guests) for the nanosphere-fullerene composite. Indeed, when we analyzed a sample of a catalytic mixture containing anthracene and $C_{70}{\subset}[Pd_6\mathbf{L^N}_{12}]$ at different stages of the catalysis, we observed initially multiple species that can be attributed to (anthracene & C_{70})$\subset[Pd_6\mathbf{L^N}_{12}]$, indicating an interaction between anthracene and the π-rich environment around and within the nanosphere. After 24h of irradiation we observe oxidized anthracene being associated with $C_{70}{\subset}[Pd_6\mathbf{L^N}_{12}]$. Whereas we anticipate major contributions to the activity of the nanosphere-fullerene composites to the enhanced solubility in different media, the attraction of hydrophobic apolar guest is an interesting observation which can be further utilized to produce even more complex or selective catalytic systems.

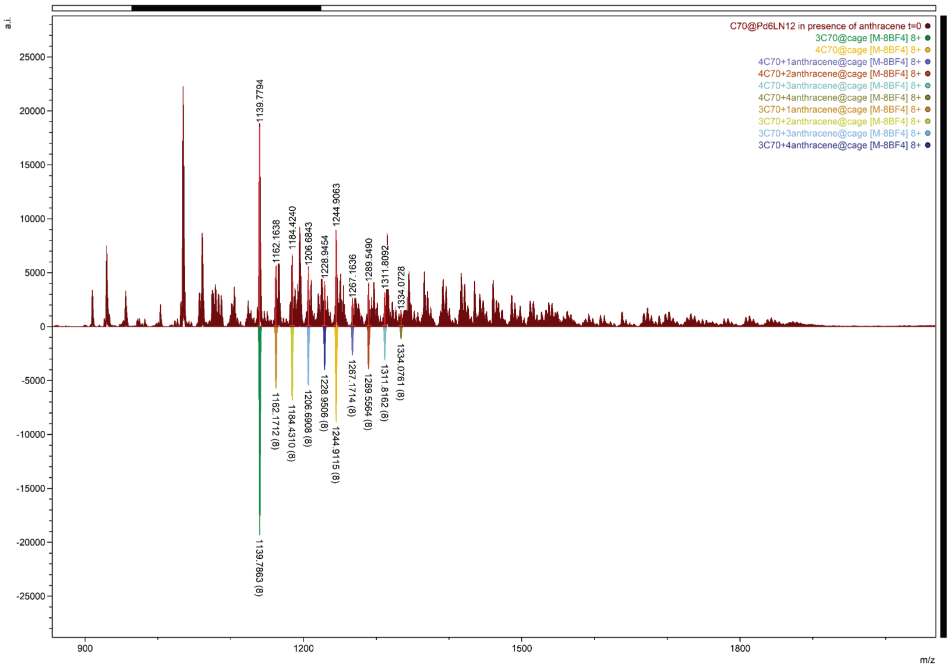

Figure S83. Full ESI-MS spectra of $C_{70}\subset Pd_6\mathbf{L^N}_{12}$ and anthracene, recorded directly after mixing. Below the simulated spectra, above obtained spectra showing up to four anthracene being associated with the $C_{70}\subset Pd_6\mathbf{L^N}_{12}$ complex.

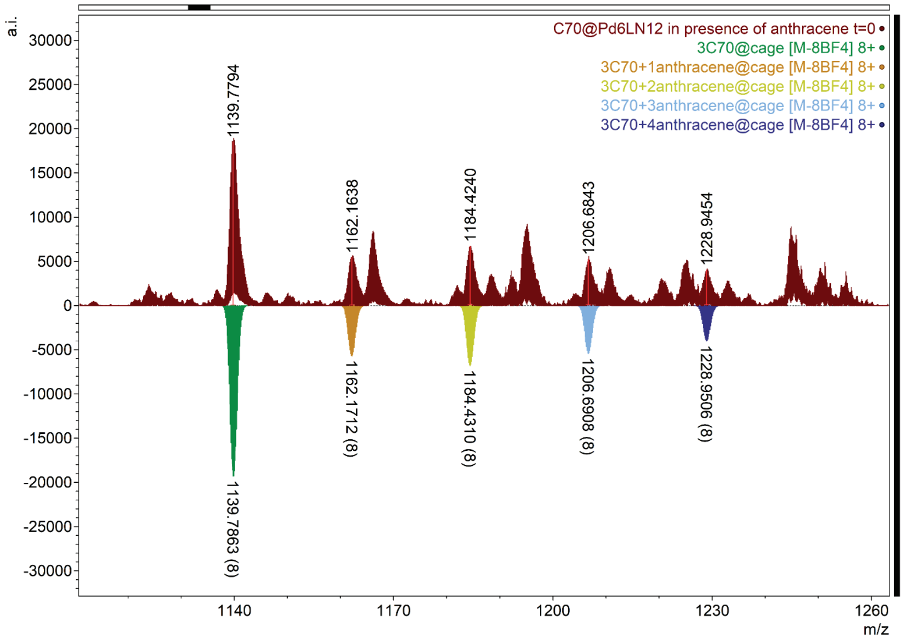

Figure S84. Zoom in ESI-MS spectra of $C_{70}\subset Pd_6\mathbf{L^N}_{12}$ and anthracene, recorded directly after mixing. Zoom into 8+ charged species of $3C_{70}\subset Pd_6\mathbf{L^N}_{12}$ carrying 0-4 anthracenes (below the simulated spectra, above obtained spectra).

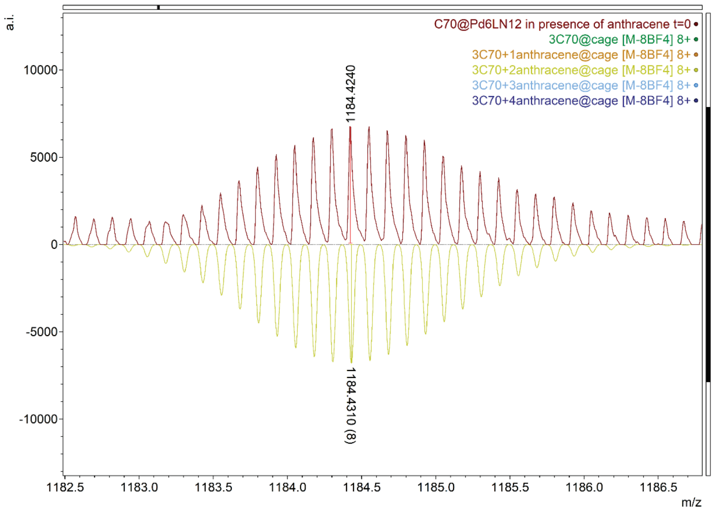

Figure S85. Zoom in ESI-MS spectra of [2antracene+$3C_{70}\subset Pd_6\mathbf{L^N}_{12}]^{8+}$, recorded directly after mixing showing a match between the simulated (bottom) and measured (top) isotope distribution.

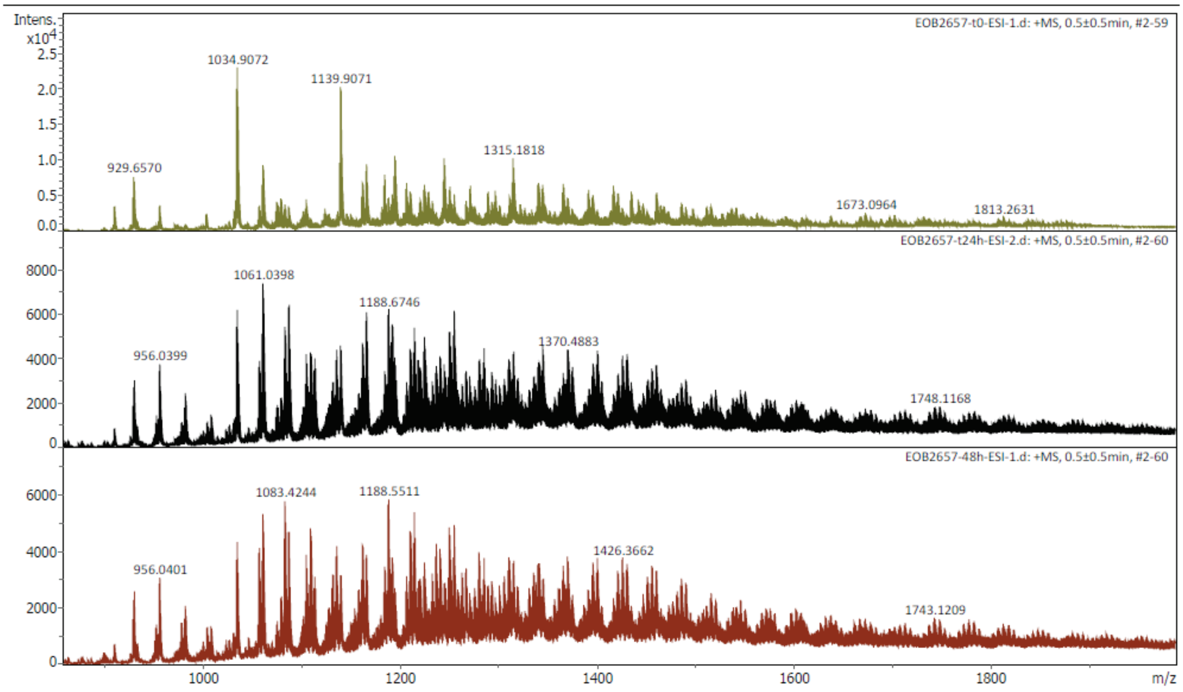

Figure S86. Development of the full ESI-MS spectra of $C_{70}\subset Pd_6\mathbf{L^N}_{12}$ and anthracene, recorded directly after mixing (top), after 24 h (middle) and after 48 h (bottom) of irradiation, showing (A) that the spheres remain intact and (B) that the anthracene being associated with the $C_{70}\subset Pd_6\mathbf{L^N}_{12}$ complex is oxidized over time.

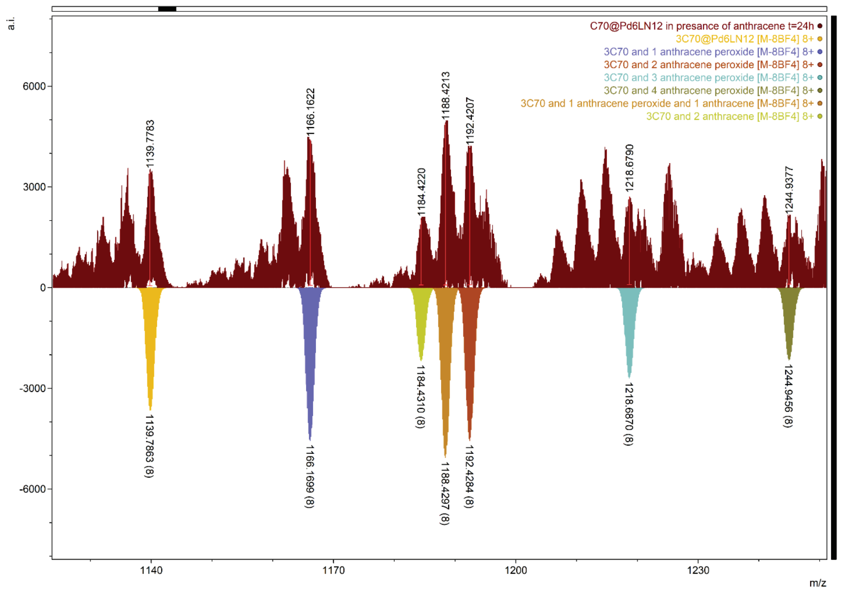

Figure S87. Zoom in ESI-MS spectra of $C_{70}{\subset}Pd_6\mathbf{L}^{\mathbf{N}}_{12}$ and anthracene, recorded after 24 h of irradiation. Zoom into 8+ charged species of $3C_{70}{\subset}Pd_6\mathbf{L}^{\mathbf{N}}_{12}$ carrying 0-4 anthracene peroxide (below the simulated spectra, above obtained spectra).

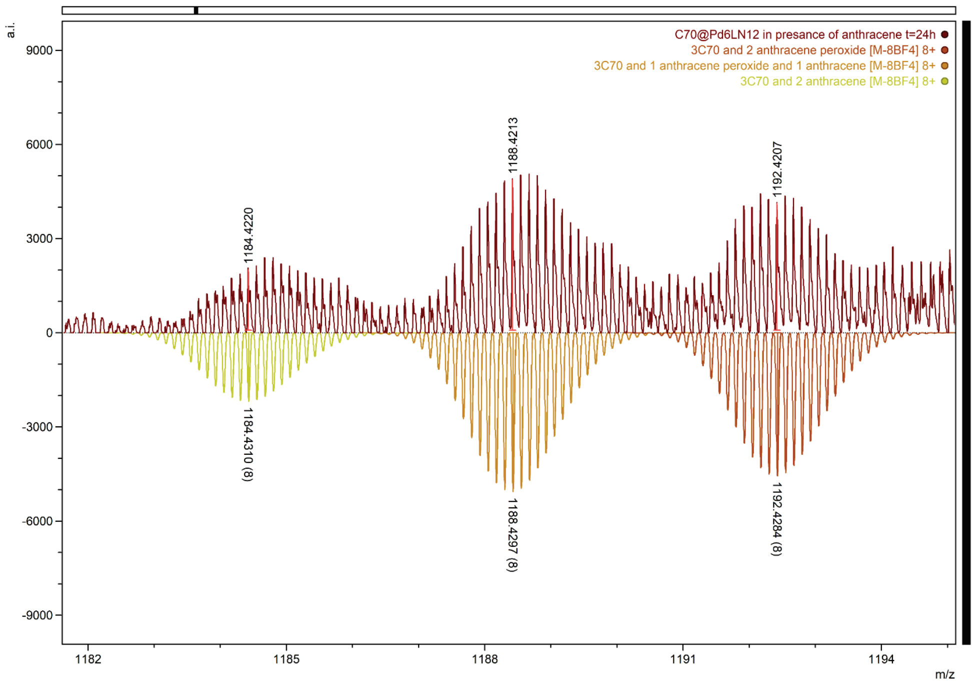

Figure S88. Zoom in ESI-MS spectra of $C_{70}{\subset}Pd_6\mathbf{L}^{\mathbf{N}}_{12}$ and anthracene, recorded after 24 h of irradiation. Zoom into 8+ charged species of $3C_{70}{\subset}Pd_6\mathbf{L}^{\mathbf{N}}_{12}$ carrying 0-2 anthracene and 0-2 anthracene peroxide (below the simulated spectra, above obtained spectra).

Water-solubility (S11)

In the main text, we describe catalytic activity of the sphere-fullerene composites in water. The experiments were performed according to the general procedure: To solutions of 10-20 μmol of substrate in 1 mL of D_2O, 5 μl of 0.83 mM solution of the sphere-fullerene complex in dmso-d_6 was added. The quartz tubes were placed 2 cm away from the light source and irradiated for 4 h at room temperature. The solubility of the fullerene complexes was attempted to be assessed by dilution of different quantities of sphere-fullerene complexes with D_2O. Regardless of the dilution amount of sphere-fullerene samples in dmf-d_7 (1.66 mM) with D_2O, we did not observe precipitation of the complexes. However, we observed in ^{1}H-NMR broadening and sharpening of the sphere signals depending on the dilution (Figure S89). We anticipate that the sphere-fullerene complex C_{70}⊂Pd_6**L**$^{PEGPy}{}_{12}$ forms nanosized aggregates when being present in high concentrations in water. At low concentration (as under catalytic conditions) relatively sharp signals were obtained, implying monomeric species. Attempts to solubilize samples in pure water were hampered because once solids were obtained by precipitation, re-solvation was not fruitful. With that, the solubility in water : dmf or water : dmso develops as follows:

DMF/DMSO content (%) in water	Sphere-Fullerene Concentration
0.5 (bio application relevant content)	8.3 μM
5 (bio application relevant content)	83 μM
10 (bio application relevant content)	166 μM (clustering of the spheres expected)
20	332 μM (clustering of the spheres expected)
30	498 μM

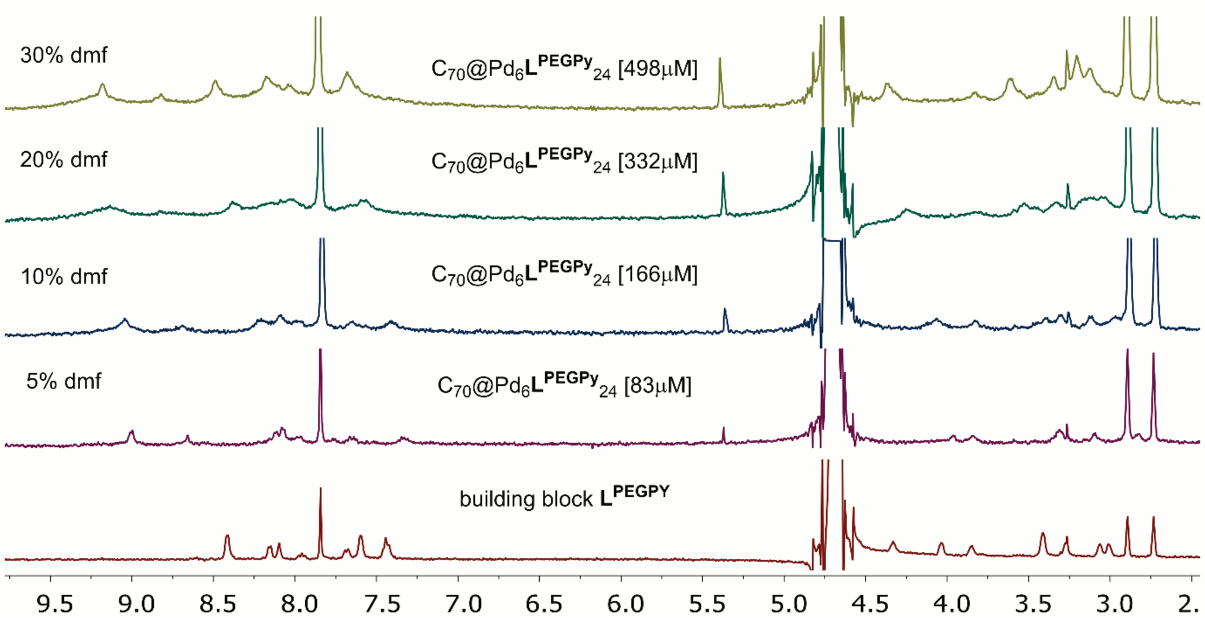

Figure S89. Dilutions of C_{70}⊂[Pd_6(**L**^{PEGPy})$_{12}$] in dmf-d7 with D2O, free building block **L**^{PEGPy} (bottom).

Catalysis in Water and Buffer (S12)

Synthesis and characterization of [Pt_6(L^{PEGPy})$_{12}$] and C_{70}⊂[Pt_6(L^{PEGPy})$_{12}$]

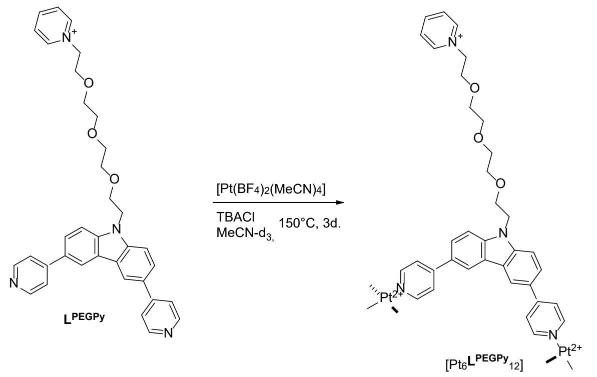

Scheme S7. Synthetic route for Pt_6(L^{PEGPy})$_{12}$.

To a solution of **L^{PEGPy}** (6.46 mg, 10 µmol, 1 eq.) in 1 mL MeCN-d_3, [Pt(BF_4)$_2$(MeCN)$_4$] (3.20 mg, 6 µmol, 0.55 eq.) and TBACl (0.1 mg) were added. The solution was then stirred at 150°C for 3d. The resulting product was precipitated in Et_2O, collected and dried under a stream of N_2 to afford Pt_6(L^{PEGPy})$_{12}$ (4.4 mg, 53%).The solid was redissolved in dmso-d_6 for further analysis and application. ^1H NMR (300 MHz, DMSO) δ 9.39 (m, 4H) , 9.08 (m, 2H), 8.86 (m, 3H), 8.43 (m, 4H), 8.07 (m, 6H), 7.77 (m, 2H), 4.61 (m, 4H), 3.72 (m, 4H). Desymmetrization/broadening of the signals are anticipated due to the slow tumbling and desymmetrization of the pyridines around the platinum cornerstones (see also [6] and [7]).The complexation with C_{70} was performed according to the same general procedure as described for the Pd analogue (S3).

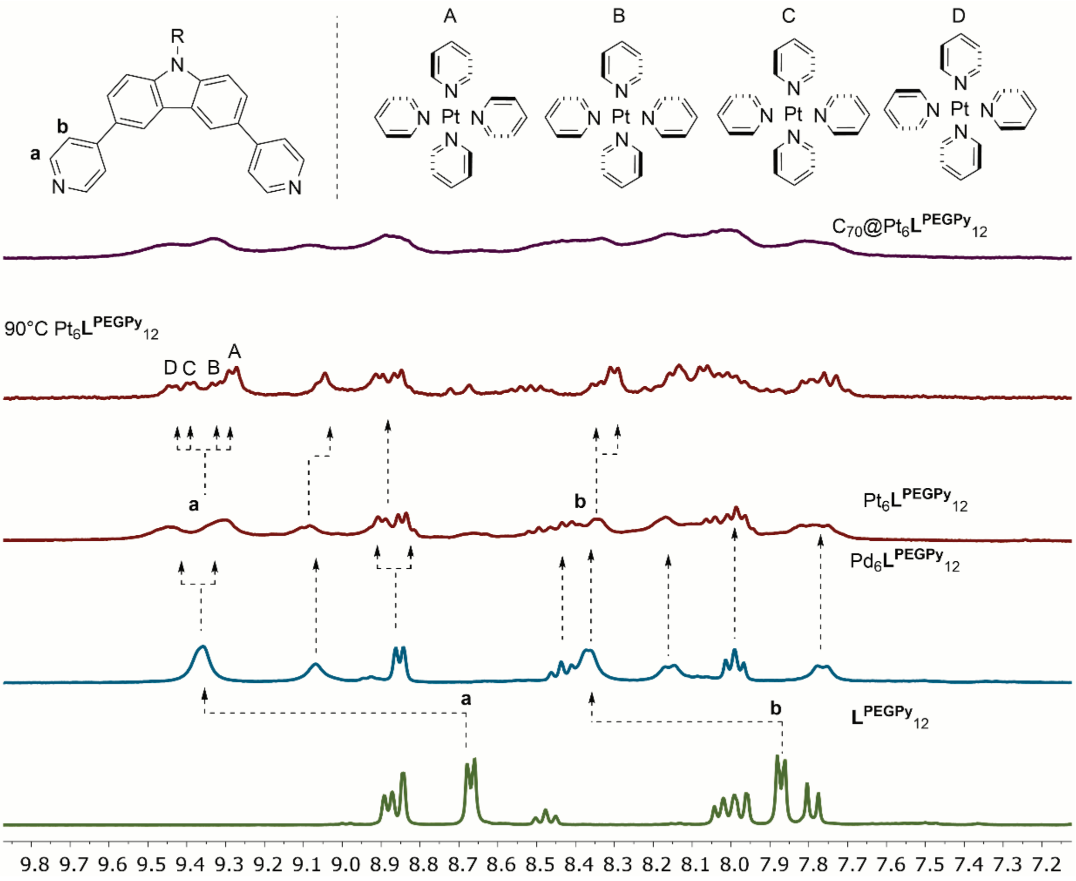

Figure S90. [Pt_6(**L^{PEGPy}**)$_{12}$] assembly (second from top), [Pd_6(**L^{PEGPy}**)$_{12}$] assembly (second from bottom) and free building block (bottom), ^1H NMR in dmso-d_6.

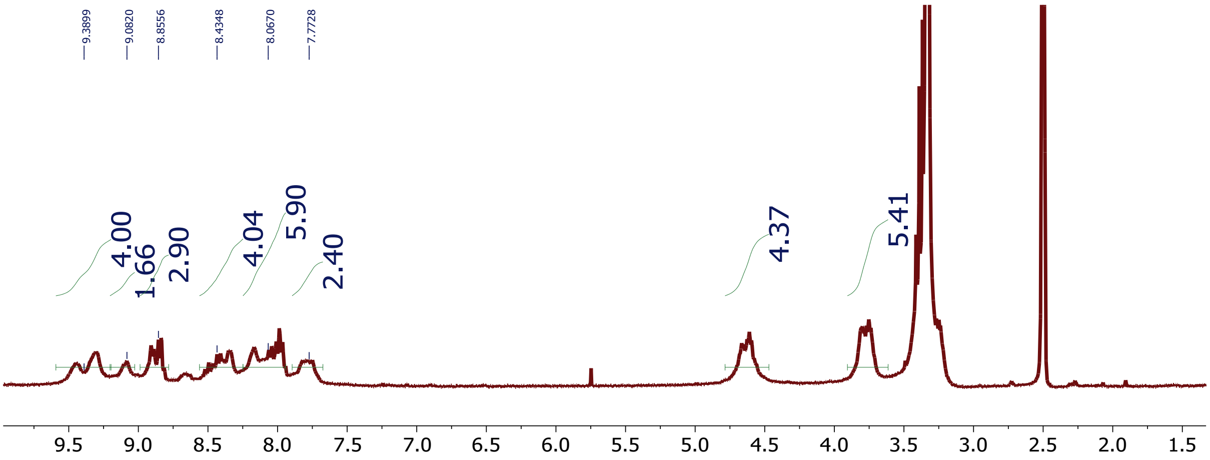

Figure S91. [Pt_6(**L**^{PEGPy})$_{12}$] assembly, ^{1}H NMR in dmso-d_6.

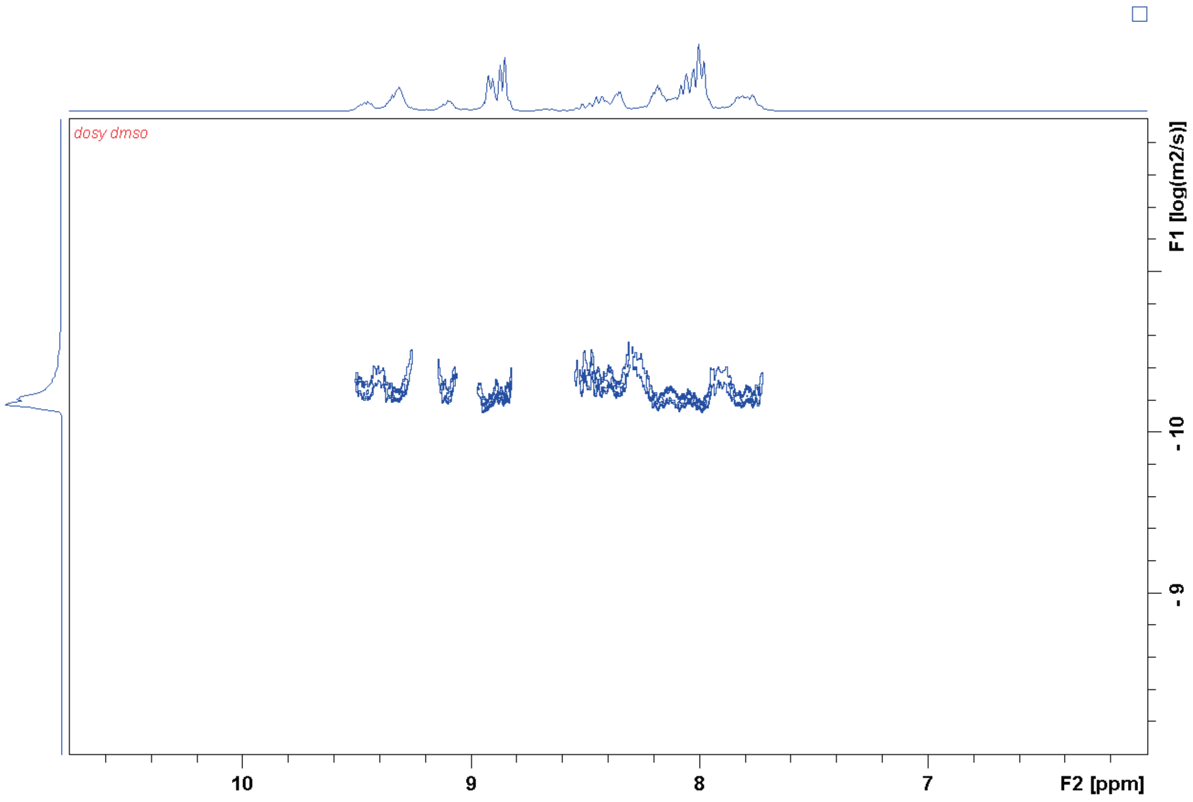

Figure S92. [Pt_6(**L**^{PEGPy})$_{12}$] assembly, DOSY NMR in dmso-d_6, zoom in into aromatic region, recorded at 300K.

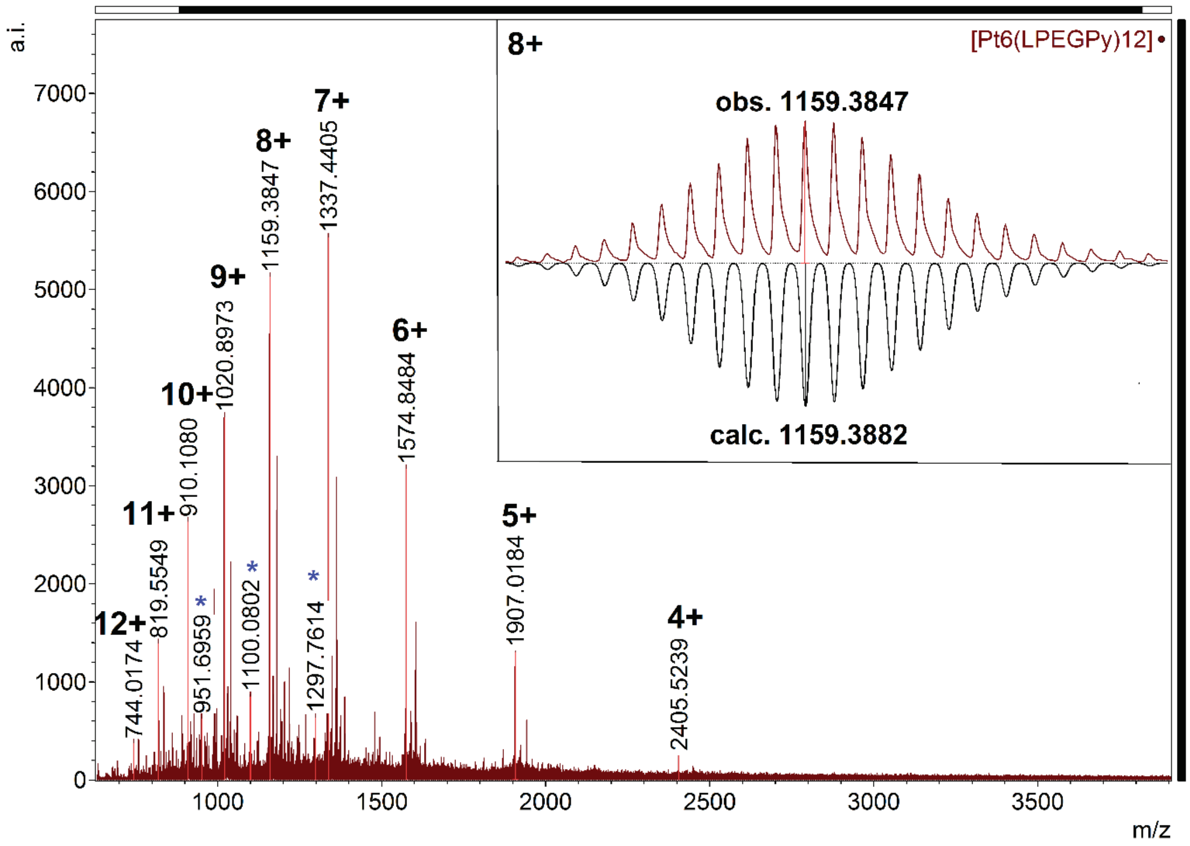

Figure S93. Full ESI-MS spectra of $[Pd_6(\mathbf{L^{PEGPy}})_{12}]^{24+}(BF_4^-)_{24}$. Below the simulated spectra, above obtained spectra.

Table S94. Zoom into ESI-MS spectra of $Pd_6\mathbf{L^{PEGPy}}_{12}$ (below the simulated spectra, above obtained spectra).

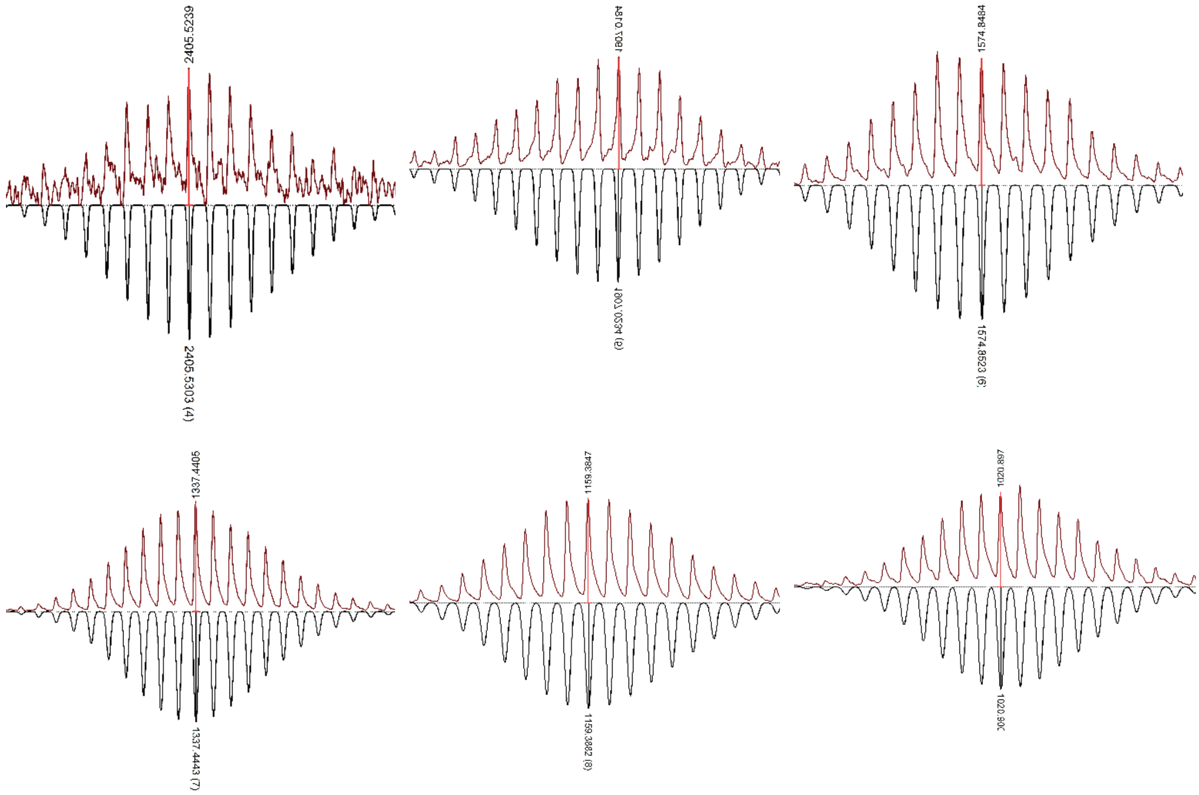

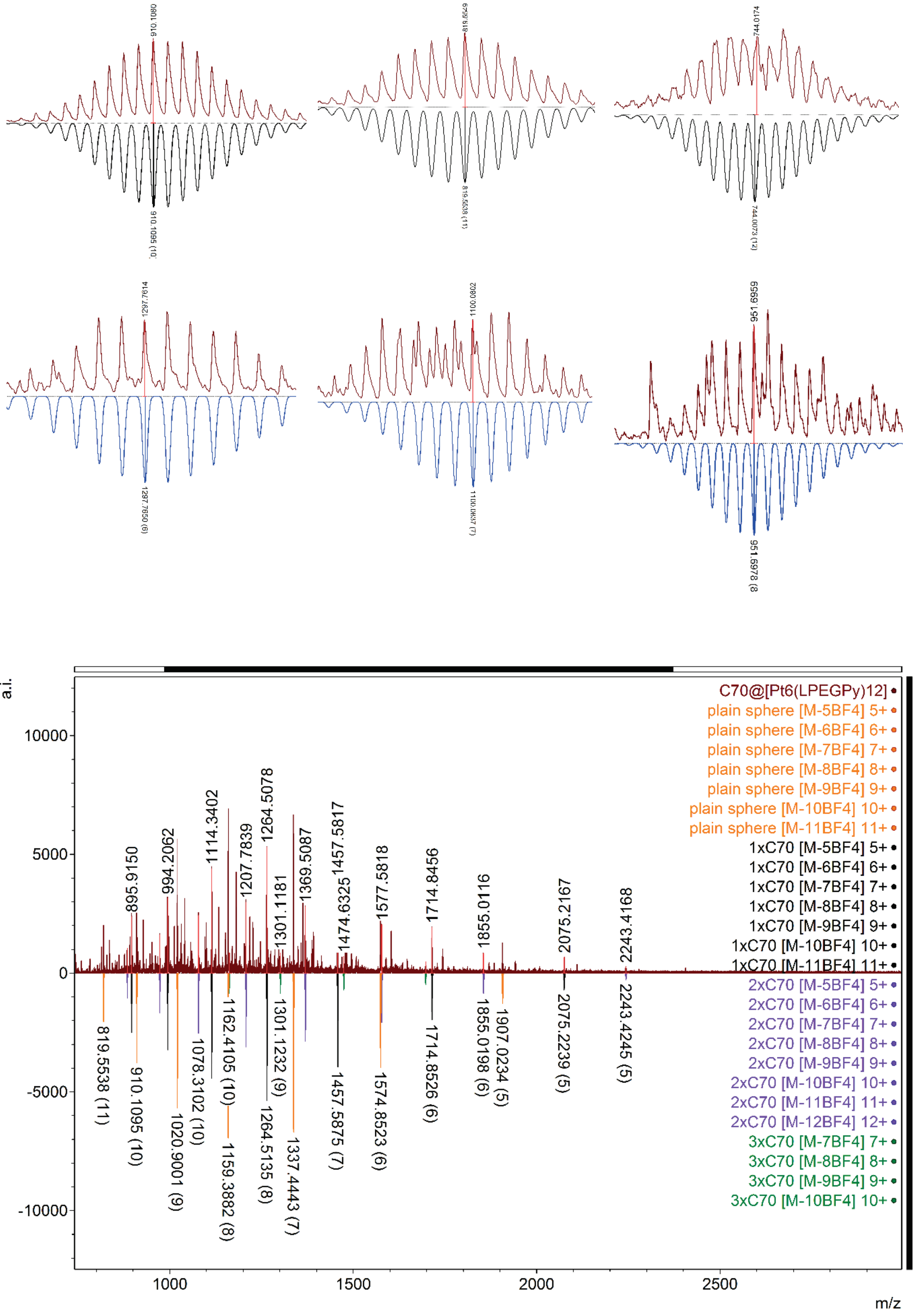

Figure S95. Full ESI-MS spectra of $C_{70}{\subset}Pt_6\mathbf{L}^{PEGPy}{}_{12}$. Below the simulated spectra, above obtained spectra.

Table S96. Zoom into ESI-MS spectra of $xC_{70}\subset Pt_6\mathbf{L}^{\mathbf{PEGPy}}_{12}$ for x=1-3 (below the simulated spectra, above obtained spectra).

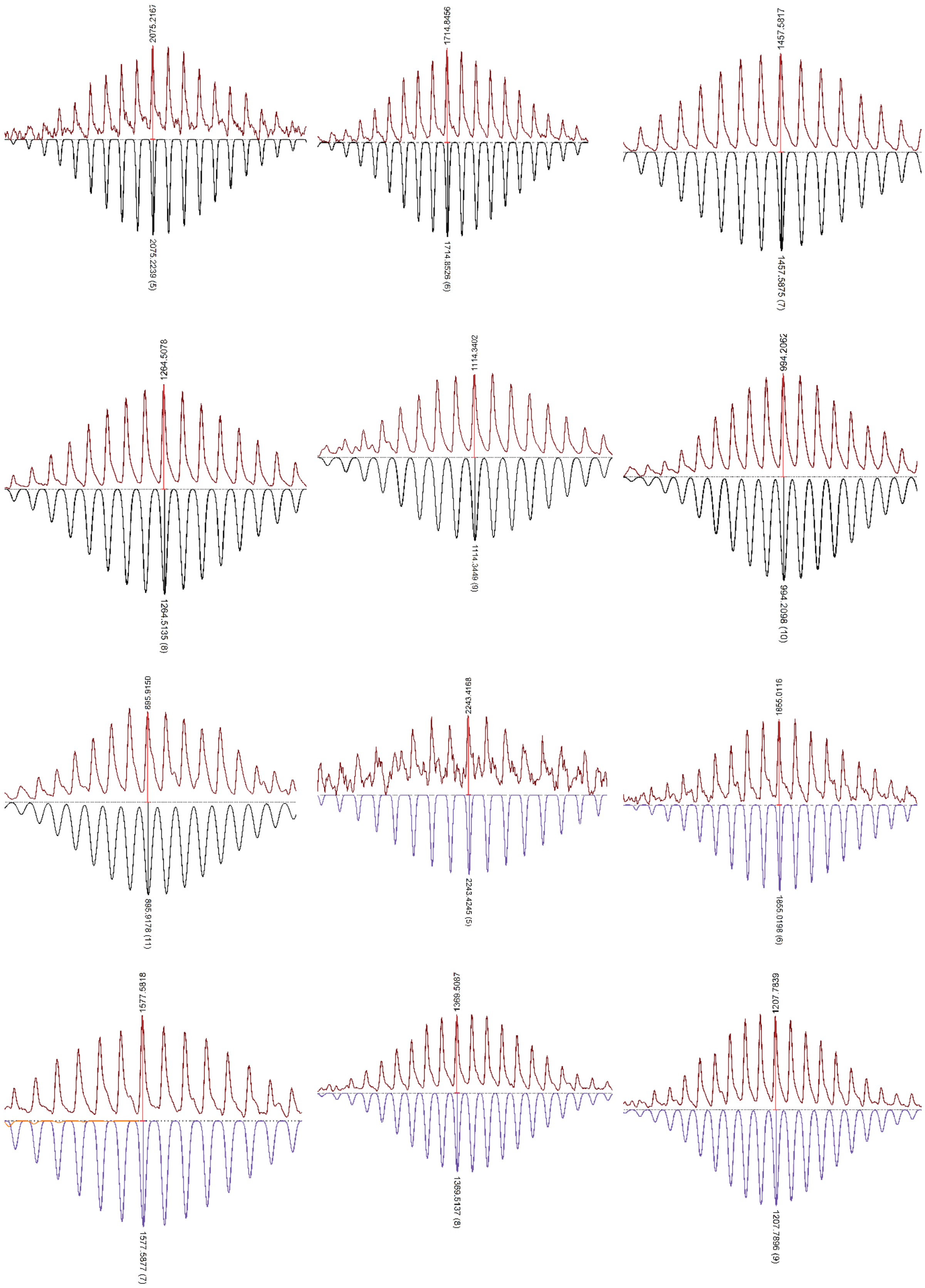

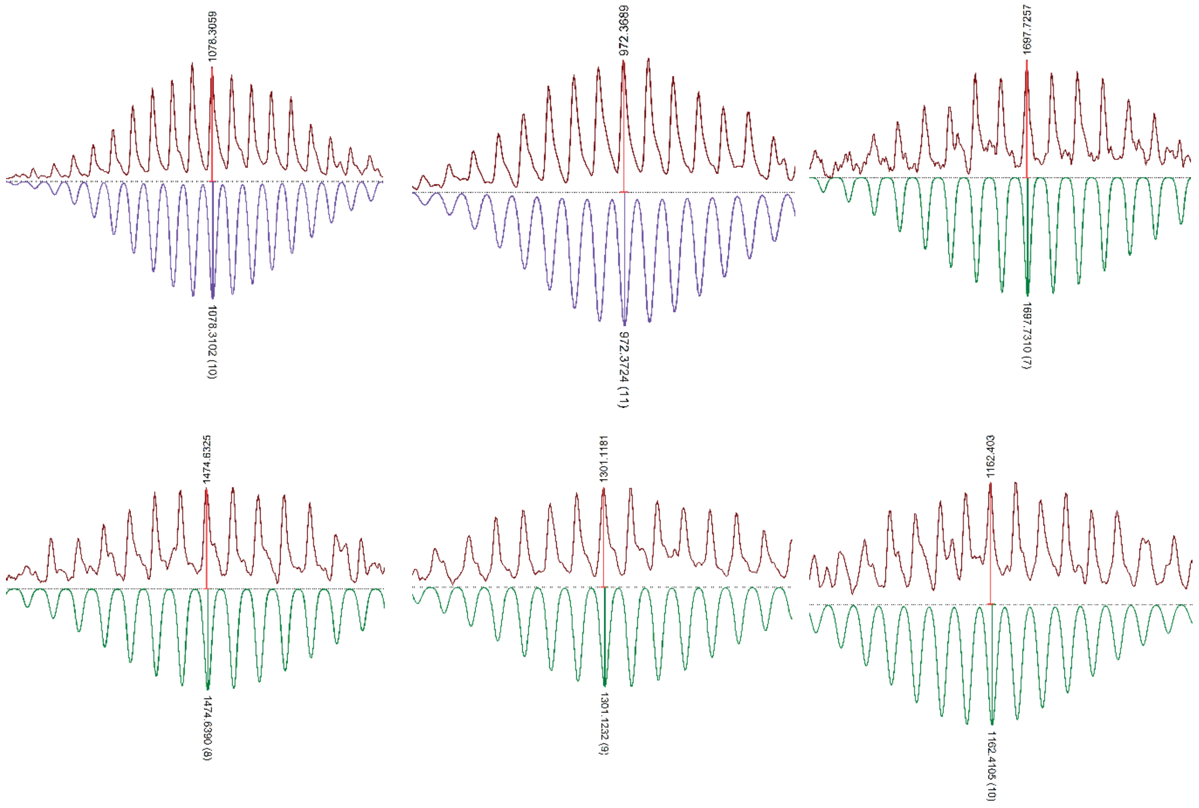

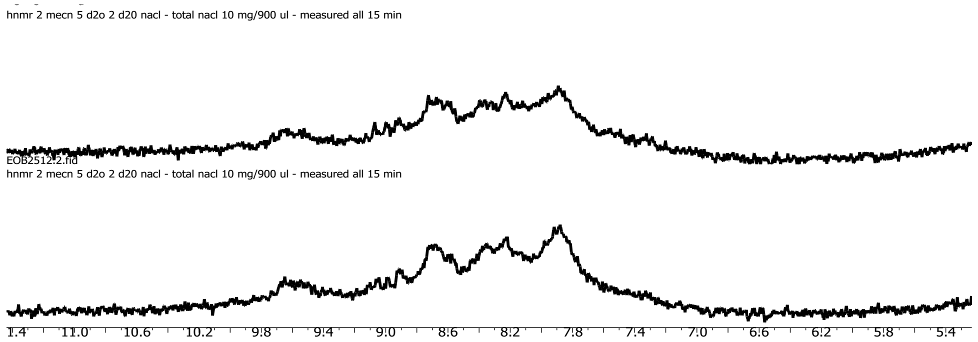

Figure S97. $C_{70}\subset[Pt_6(\mathbf{L}^{\mathbf{PEGPy}})_{12}]$ in MeCN-d3 with D2O and NaCl displaying no changes after 10h at 37°C. Broadening of the signal is anticipated due to formation of larger aggregates similar to palladium analogue (S88).

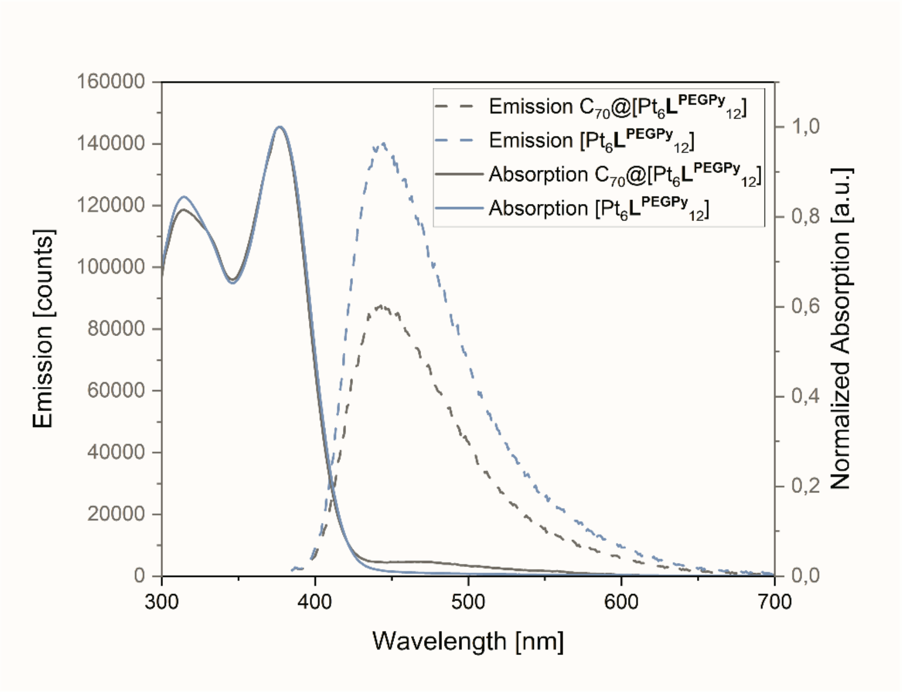

Figure S98. $[Pt_6(\mathbf{L}^{PEGPy})_{12}]$ and $C_{70}\subset[Pt_6(\mathbf{L}^{PEGPy})_{12}]$ absorption and emission spectra at equivalent sphere concentration. Recorded in 10 mM HEPES buffer.

Catalysis in Water and Buffer

General procedure: To solutions/suspensions of 10 µmol of substrate in 1 mL of the corresponding solvent, 5 µl of 0.83 mM solution of the corresponding sphere or sphere-fullerene complex in dmso-d_6 was added (4.16 nmol). The quartz tubes were placed 2 cm away from the light source and irradiated for 4 h at room temperature. Afterwards, maleic acid was added as standard and the yields of product determined by ^{1}H-NMR. The reactions were performed in (A) D_2O and (B) in 50% 1N PBD buffered H_2O and 50% D_2O.

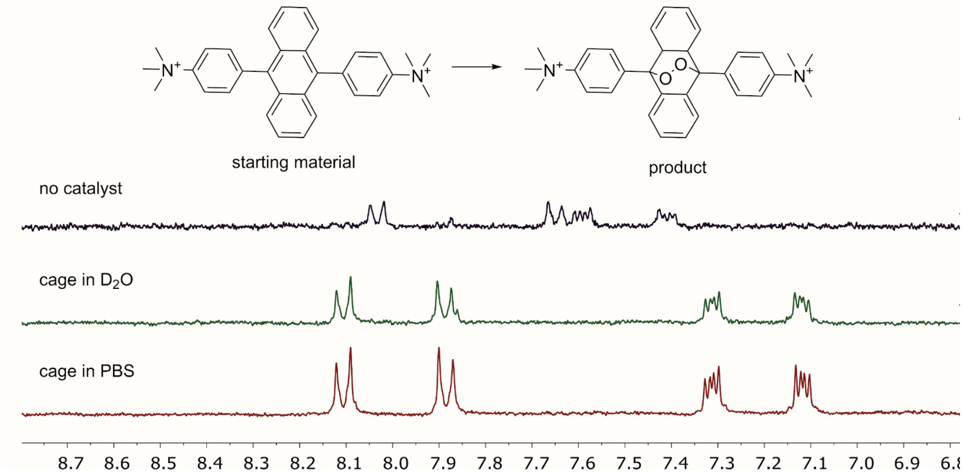

Figure S99. Selected region of the ^{1}H-NMR spectra of the photocatalytic peroxidation of substituted anthracene.

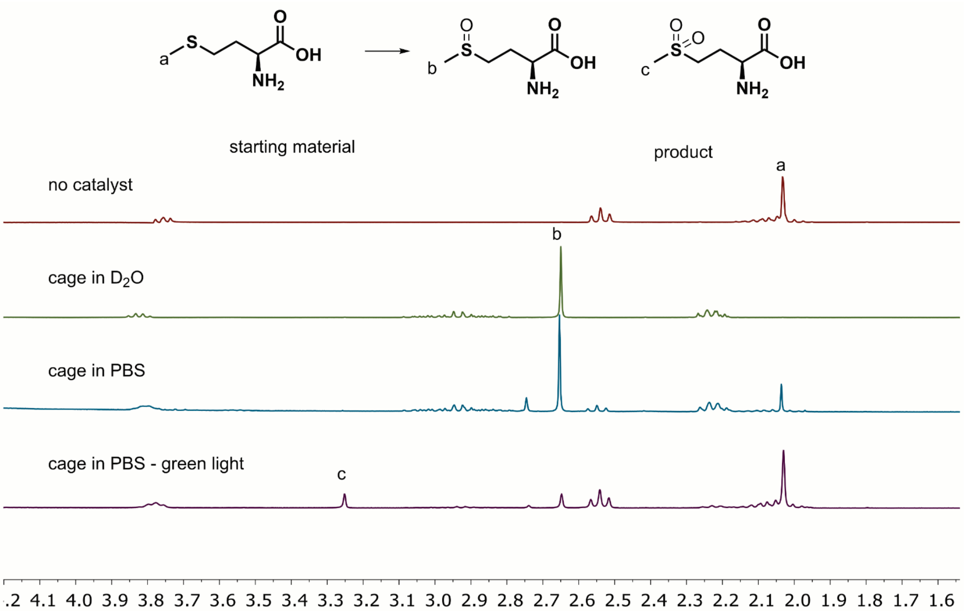

Figure S100. Selected region of the ^{1}H-NMR spectra of the photocatalytic oxidation of methionine.

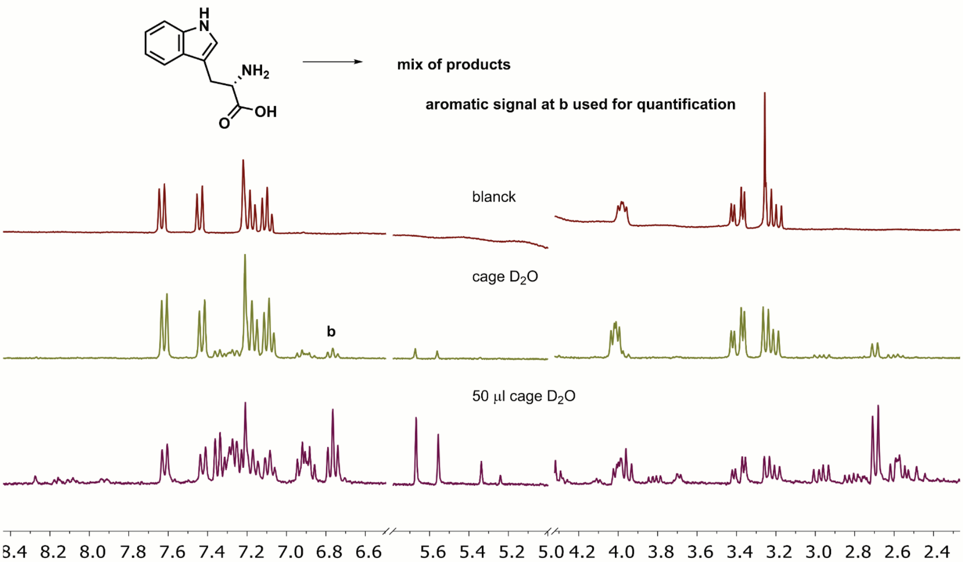

Figure S101. Selected region of the ^{1}H-NMR spectra of the photocatalytic oxidation of tryptophane.

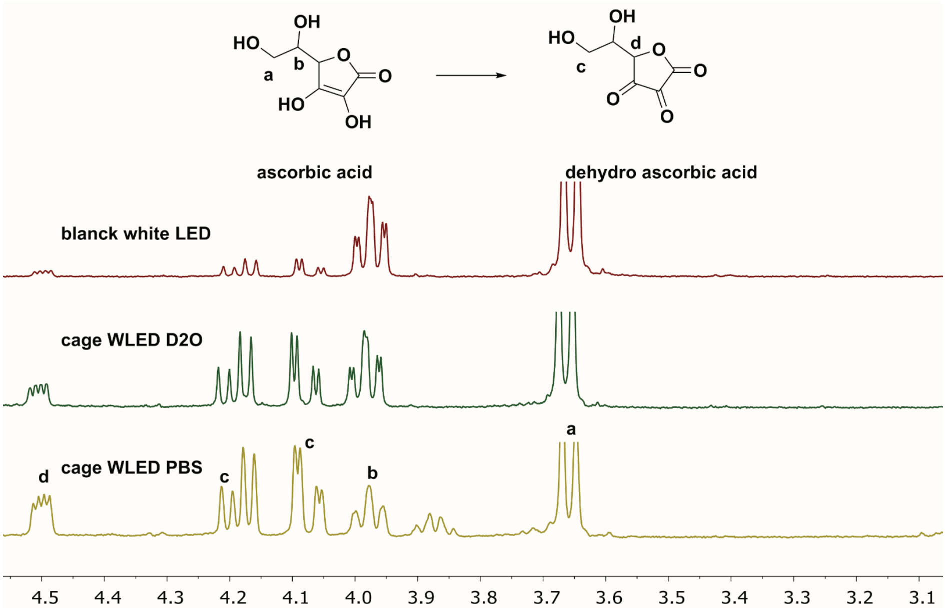

Figure S102. Selected region of the ^{1}H-NMR spectra of the photocatalytic oxidation of ascorbic acid.

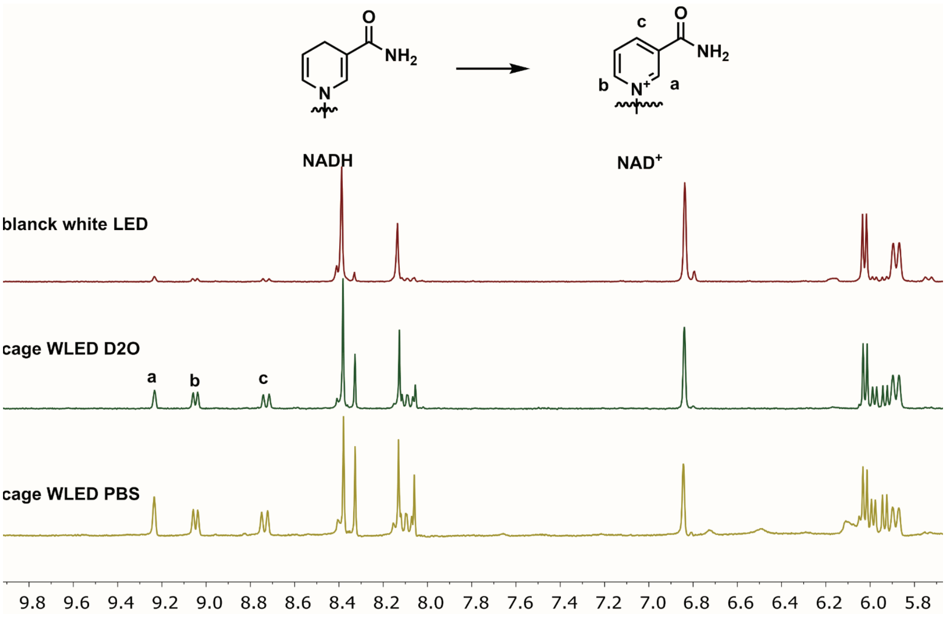

Figure S103. Selected region of the ^{1}H-NMR spectra of the photocatalytic oxidation of NADH.

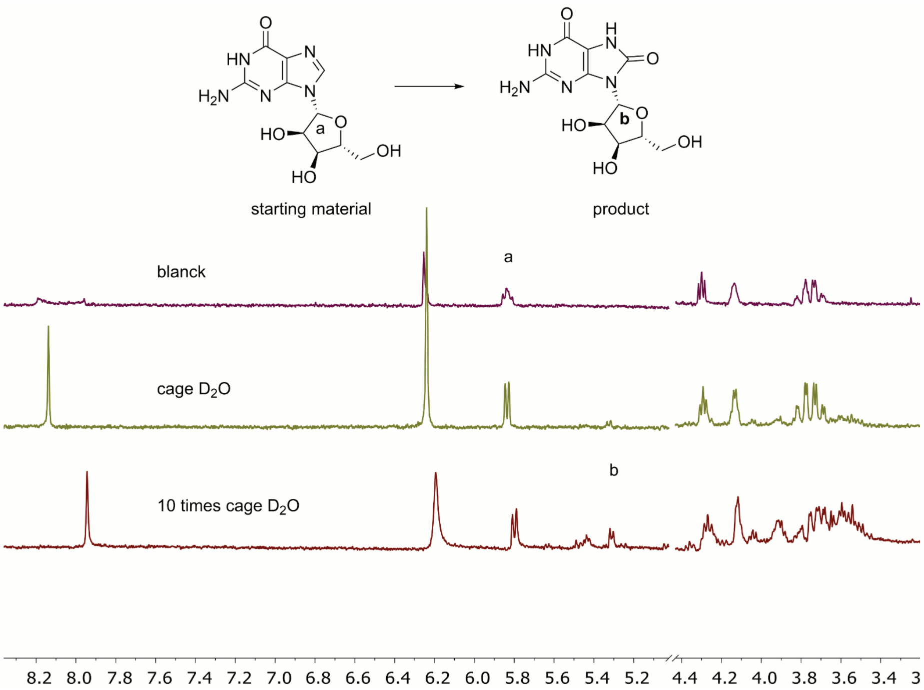

Figure S104. Selected region of the ^{1}H-NMR spectra of the photocatalytic oxidation of guanosine.

References (S13)

1. Bruggeman DF, Laporte AAH, Detz RJ, Mathew S, Reek JNH. Aqueous Biphasic Dye-Sensitized Photosynthesis Cells for TEMPO-Based Oxidation of Glycerol. Angewandte Chemie 2022; 134.
2. Bobylev EduardO, de Bruin B, Reek JNH. Catalytic Formation of Coordination-Based Self-Assemblies by Halide Impurities. Inorg Chem 2021; 60:12498–12505.
3. http://supramolecular.org/
4. Connolly ML. Solvent-Accessible Surfaces of Proteins and Nucleic Acids. Science 1983; 221:709–713.
4. Fulmer GR, Miller AJM, Sherden NH, et al. NMR Chemical Shifts of Trace Impurities: Common Laboratory Solvents, Organics, and Gases in Deuterated Solvents Relevant to the Organometallic Chemist. Organometallics 2010; 29:2176–2179.
5. Bryant RG. The NMR time scale. J Chem Educ 1983; 60:933.
6. Bobylev EO, Poole DA III, Bruin B, Reek JNH. How to Prepare Kinetically Stable Self-assembled Pt 12 L 24 Nanocages while Circumventing Kinetic Traps. Chem Eur J 2021; 27:12667–12674.
7. Tsutsui T, Kusaba S, Yamashina M, Akita M, Yoshizawa M. Open versus Closed Polyaromatic Nanocavity: Enhanced Host Abilities toward Large Dyes and Pigments. Chem Eur J 2019; 25:4320–4324.
